# Supplementary material for: Stereoselective Synthesis of 24-Fluoro-25-Hydroxyvitamin D3 Analogues and Their Stability to hCYP24A1-Dependent Catabolism
Source: Int J Mol Sci. 2021 Nov 1;22(21):11863. doi: 10.3390/ijms222111863 (PMC8584271; doi:10.3390/ijms222111863)
Supplement: Supplementary file 1 [file ijms-22-11863-s001.zip › ijms-1405068-supplementary.pdf]

Supporting Information

Stereoselective Synthesis of 24-Fluoro-25-Hydroxyvitamin D_3 Analogues and Their Stability to hCYP24A1-dependent Catabolism

Fumihiro Kawagoe,[1] Sayuri Mototani,[1] Kaori Yasuda,[2]

Hiroki Mano,[2] Toshiyuki Sakaki,[2] and Atsushi Kittaka*,[1]

1 *Faculty of Pharmaceutical Sciences, Teikyo University, 2-11-1 Kaga, Itabashi, Tokyo 173-8605, Japan*;
2 *Faculty of Engineering, Toyama Prefectural University, Imizu, Toyama 939-0398, Japan;*

* Corresponding author. Tel.: +81-3-3964-8109; Fax: +81-3-3964-8117.

E-mail address: akittaka@pharm.teikyo-u.ac.jp (A. Kittaka).

Contents

Title page S1

^{1}H and ^{13}C NMR spectra of all new compounds:
19-21, **24**, **25**, **28-31**, **9-12**, **15**, **16**, **3**, **4**, **36**, and **37**. S2-S39

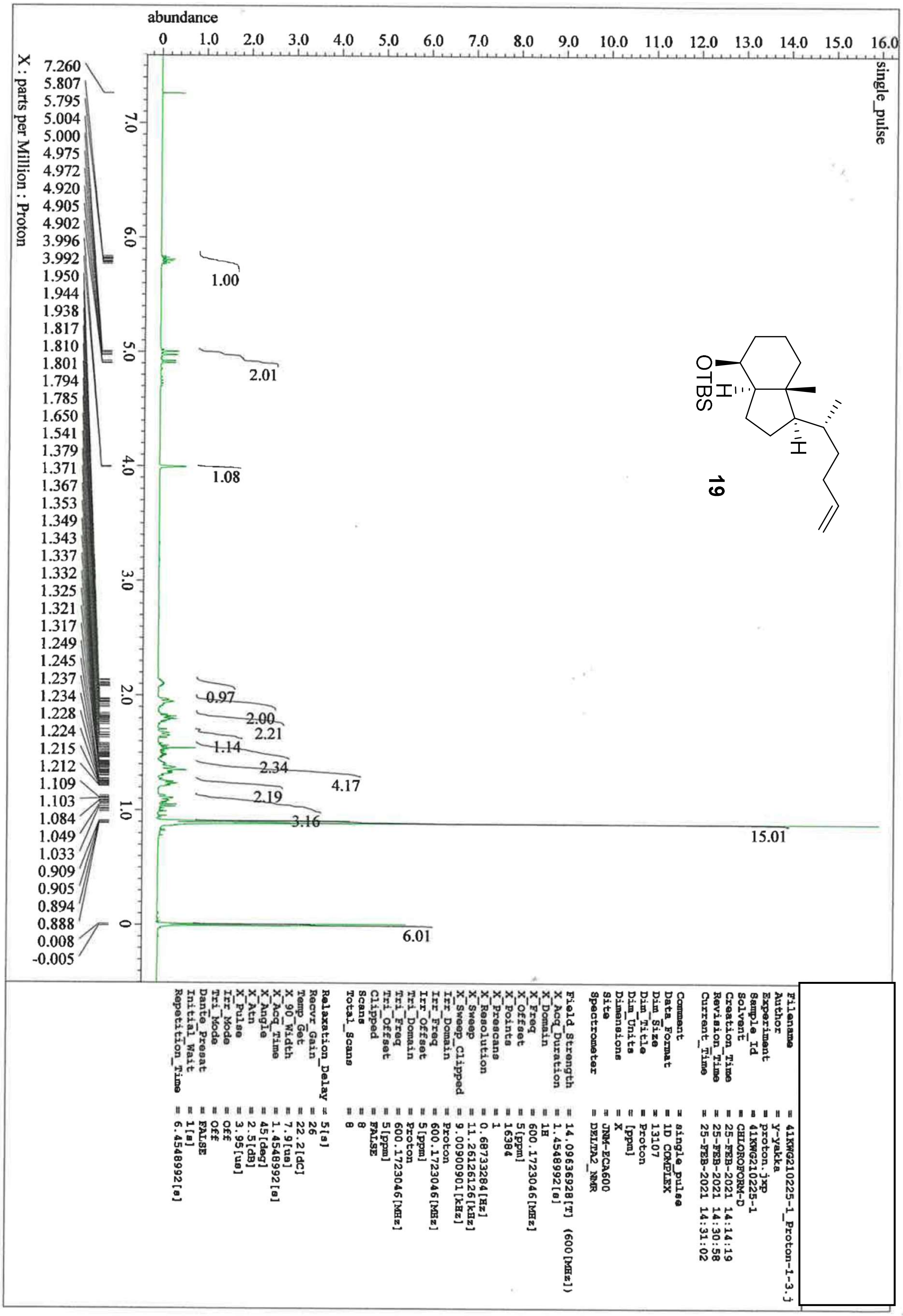
abundance
single_pulse
X : parts per Million : Proton
19
OTBS
H
1.00
2.01
1.08
0.97
2.00
2.21
1.14
2.34
4.17
2.19
3.16
15.01
6.01
Filename = 41KWG210225-1_Proton-1-3.j
Author = y-yaka
Experiment = proton.jxp
Sample_Id = 41KWG210225-1
Solvent = CHLOROFORM-D
Creation_Time = 25-FEB-2021 14:14:19
Revision_Time = 25-FEB-2021 14:30:58
Current_Time = 25-FEB-2021 14:31:02
Comment = single_pulse
Data_Format = 1D COMPLEX
Dim_Size = 13107
Dim_Title = Proton
Dim_Units = [ppm]
Dimensions = X
Site = JNM-ECA600
Spectrometer = DELTA2_NMR
Field_Strength = 14.09636928[T] (600[MHz])
X_Acq_Duration = 1.4548992[s]
X_Domain = 1H
X_Freq = 600.1723046[MHz]
X_Offset = 5[ppm]
X_Points = 16384
X_Prescans = 1
X_Resolution = 0.68733284[Hz]
X_Sweep = 11.26126126[kHz]
X_Sweep_Clipped = 9.00900901[kHz]
Irr_Domain = Proton
Irr_Freq = 600.1723046[MHz]
Irr_Offset = 5[ppm]
Tri_Domain = Proton
Tri_Freq = 600.1723046[MHz]
Tri_Offset = 5[ppm]
Clipped = FALSE
Scans = 8
Total_Scans = 8
Relaxation_Delay = 5[s]
Recvr_Gain = 26
Temp_Get = 22.2[dC]
X_90_Width = 7.9[us]
X_Acq_Time = 1.4548992[s]
X_Angle = 45[deg]
X_Atn = 2.5[dB]
X_Pulse = 3.95[us]
Irr_Mode = Off
Tri_Mode = Off
Dante_Presat = FALSE
Initial_Wait = 1[s]
Repetition_Time = 6.4548992[s]

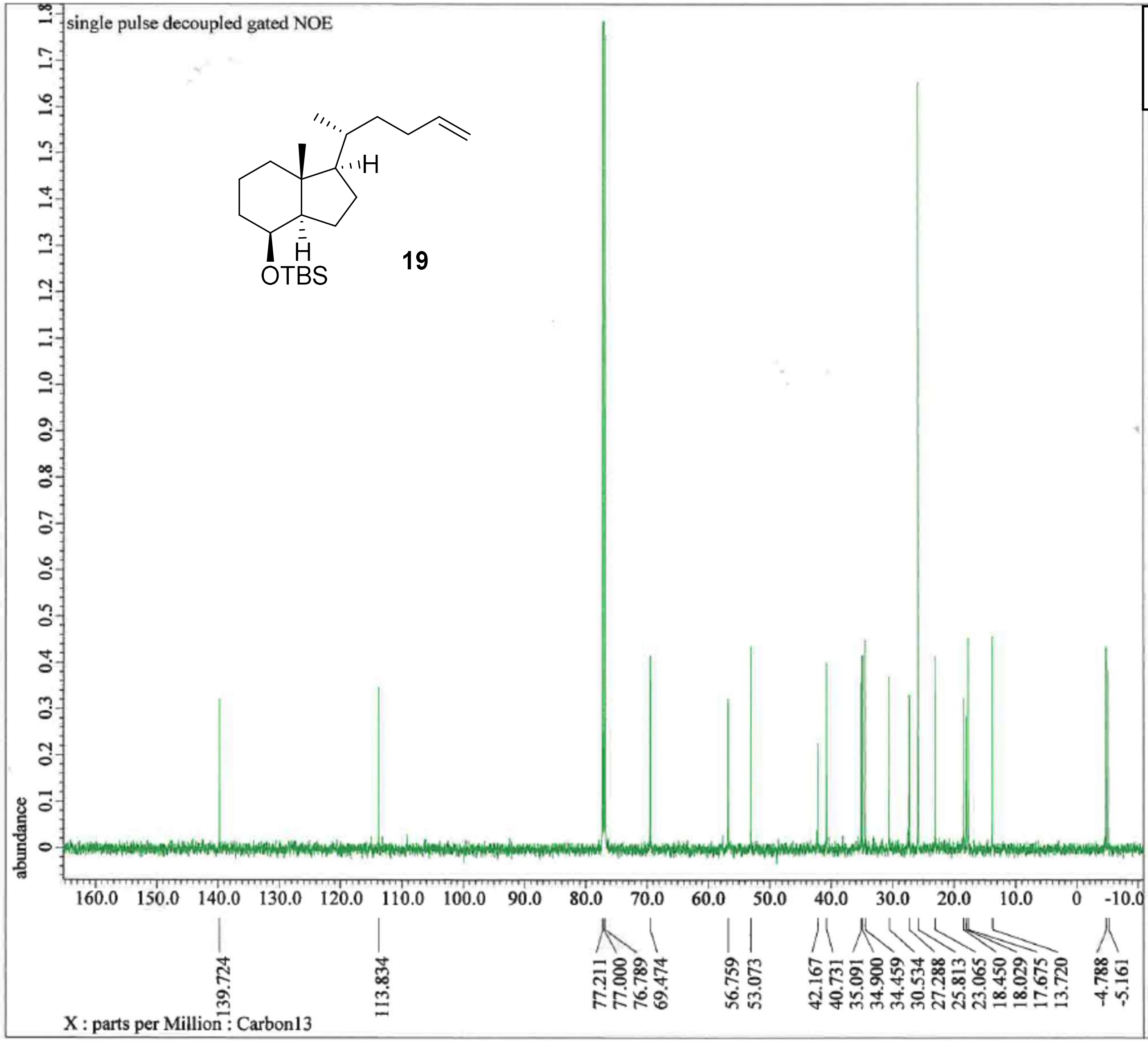

Filename	= 41KWG210225-1_Carbon_copy1
Author	= y-yakka
Experiment	= carbon.jxp
Sample_Id	= 41KWG210225-1
Solvent	= CHLOROFORM-D
Creation_Time	= 25-FEB-2021 14:18:03
Revision_Time	= 25-FEB-2021 14:26:37
Current_Time	= 25-FEB-2021 14:28:09
Comment	= single pulse decoupled gat
Data_Format	= 1D COMPLEX
Dim_Size	= 26214
Dim_Title	= Carbon13
Dim_Units	= [ppm]
Dimensions	= X
Site	= JNM-ECA600
Spectrometer	= DELTA2_NMR
Field_Strength	= 14.09636928[T] (600[MHz])
X_Acq_Duration	= 0[s]
X_Domain	= 13C
X_Freq	= 150.91343039[MHz]
X_Offset	= 100[ppm]
X_Points	= 32768
X_Prescans	= 4
X_Resolution	= 1.44496109[Hz]
X_Sweep	= 47.34848485[kHz]
X_Sweep_Clipped	= 37.87878788[kHz]
Irr_Domain	= Proton
Irr_Freq	= 600.1723046[MHz]
Irr_Offset	= 5[ppm]
Clipped	= FALSE
Incomplete_Copy	= TRUE
Scans	= 154
Total_Scans	= 154
Relaxation_Delay	= 2[s]
Recvr_Gain	= 58
Temp_Get	= 22.3[dC]
X_90_Width	= 13.7[us]
X_Acq_Time	= 0.69206016[s]
X_Angle	= 30[deg]
X_Atn	= 14[dB]
X_Pulse	= 4.56666667[us]
Irr_Atn_Dec	= 22.164[dB]
Irr_Atn_Noe	= 22.164[dB]
Irr_Noise	= WALTZ
Irr_Pwidth	= 76[us]
Decoupling	= TRUE
Initial_Wait	= 1[s]
Noe	= TRUE
Noe_Time	= 2[s]
Repetition_Time	= 2.69206016[s]

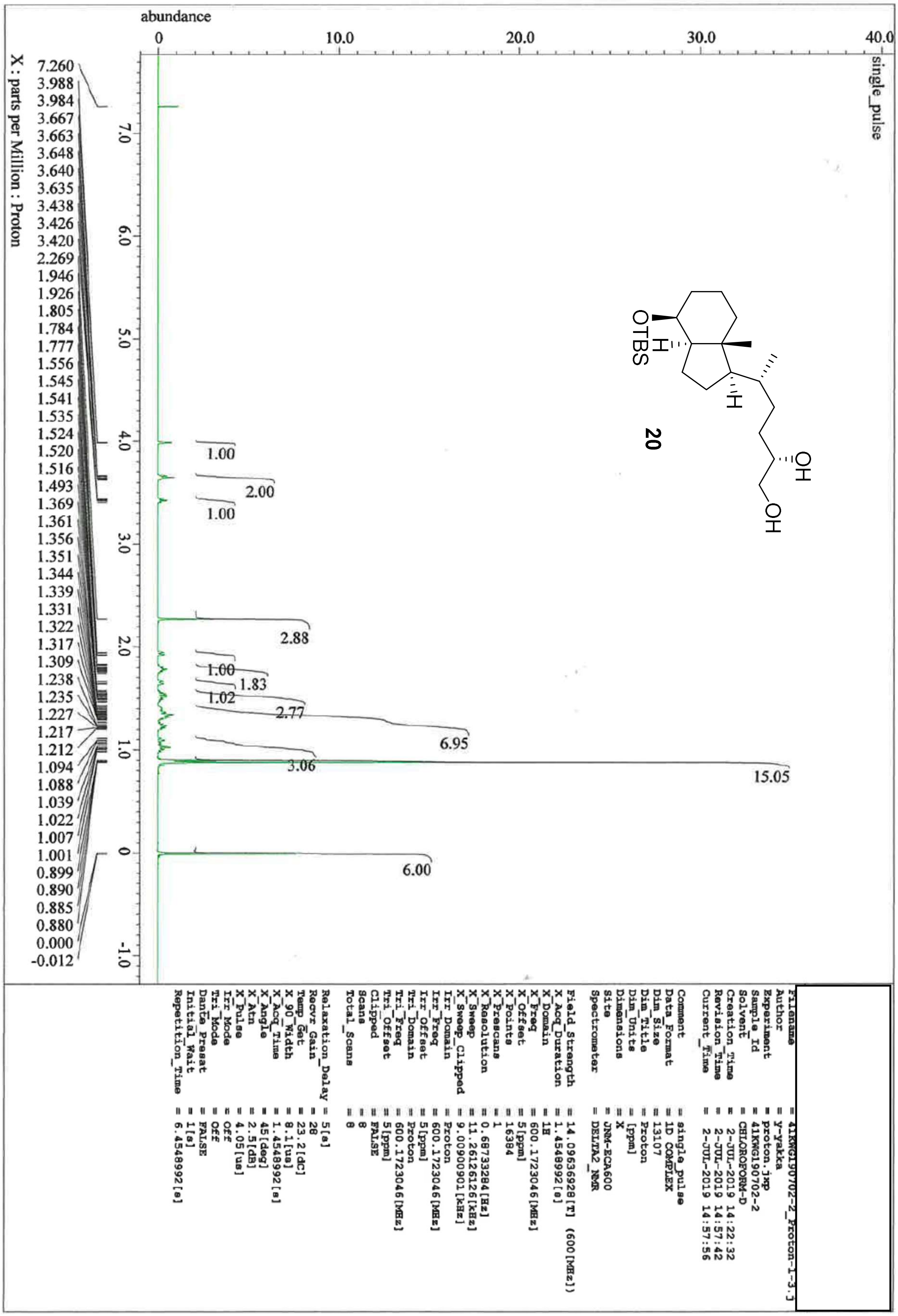
abundance
0
10.0
20.0
30.0
40.0
single_pulse
X : parts per Million : Proton
7.260 3.988 3.984 3.667 3.663 3.648 3.640 3.635 3.438 3.426 3.420 2.269 1.946 1.926 1.805 1.784 1.777 1.556 1.545 1.541 1.535 1.524 1.520 1.516 1.493 1.369 1.361 1.356 1.351 1.344 1.339 1.331 1.322 1.317 1.309 1.238 1.235 1.227 1.217 1.212 1.094 1.088 1.039 1.022 1.007 1.001 0.899 0.890 0.885 0.880 0.000 -0.012
7.0
6.0
5.0
4.0
3.0
2.0
1.0
0
-1.0
1.00
2.00
1.00
2.88
1.00
1.83
1.02
2.77
6.95
3.06
15.05
6.00
OTBS
H
H
OH
OH
20
Filename = 41KMG190702-2_Proton-1-3.j
Author = y-yakka
Experiment = proton.jxp
Sample_Id = 41KMG190702-2
Solvent = CHLOROFORM-D
Creation_Time = 2-JUL-2019 14:22:32
Revision_Time = 2-JUL-2019 14:57:42
Current_Time = 2-JUL-2019 14:57:56
Comment = single_pulse
Data_Format = 1D COMPLEX
Dim_Size = 13107
Dim_Title = Proton
Dim_Units = [ppm]
Dimensions = X
Site = JNM-ECA600
Spectrometer = DELTA2_NMR
Field_Strength = 14.09636928[T] (600[MHz])
X_Acq_Duration = 1.4548992[s]
X_Domain = 1H
X_Freq = 600.1723046[MHz]
X_Offset = 5[ppm]
X_Points = 16384
X_Prescans = 1
X_Resolution = 0.68733284[Hz]
X_Sweep = 11.26126126[kHz]
X_Sweep_Clipped = 9.00900901[kHz]
Irr_Domain = Proton
Irr_Freq = 600.1723046[MHz]
Irr_Offset = 5[ppm]
Tri_Domain = Proton
Tri_Freq = 600.1723046[MHz]
Tri_Offset = 5[ppm]
Clipped = FALSE
Scans = 8
Total_Scans = 8
Relaxation_Delay = 5[s]
Recvr_Gain = 28
Temp_Get = 23.2[dC]
X_90_Width = 8.1[us]
X_Acq_Time = 1.4548992[s]
X_Angle = 45[deg]
X_Atn = 2.5[dB]
X_Pulse = 4.05[us]
Irr_Mode = Off
Tri_Mode = Off
Dante_Presat = FALSE
Initial_Wait = 1[s]
Repetition_Time = 6.4548992[s]

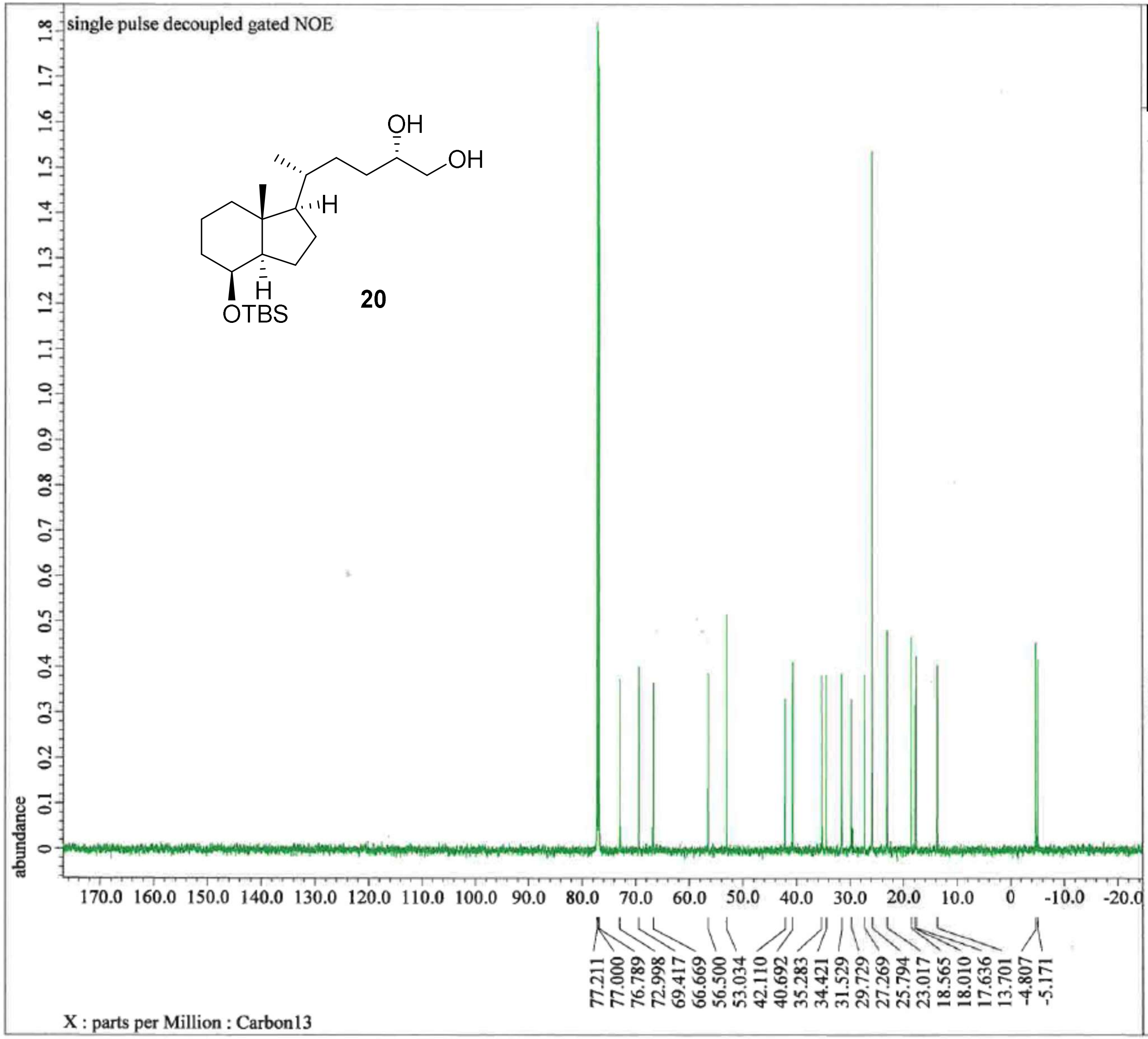

Filename	= 41KWG190702-2_Carbon-1-3.j
Author	= y-yakka
Experiment	= carbon.jxp
Sample_Id	= 41KWG190702-2
Solvent	= CHLOROFORM-D
Creation_Time	= 2-JUL-2019 14:29:53
Revision_Time	= 2-JUL-2019 15:01:29
Current_Time	= 2-JUL-2019 15:02:25
Comment	= single pulse decoupled gat
Data_Format	= 1D COMPLEX
Dim_Size	= 26214
Dim_Title	= Carbon13
Dim_Units	= [ppm]
Dimensions	= X
Site	= JNM-ECA600
Spectrometer	= DELTA2_NMR
Field_Strength	= 14.09636928[T] (600[MHz])
X_Acq_Duration	= 0.69206016[s]
X_Domain	= 13C
X_Freq	= 150.91343039[MHz]
X_Offset	= 100[ppm]
X_Points	= 32768
X_Prescans	= 4
X_Resolution	= 1.44496109[Hz]
X_Sweep	= 47.34848485[kHz]
X_Sweep_Clipped	= 37.87878788[kHz]
Irr_Domain	= Proton
Irr_Freq	= 600.1723046[MHz]
Irr_Offset	= 5[ppm]
Clipped	= FALSE
Scans	= 256
Total_Scans	= 256
Relaxation_Delay	= 2[s]
Recvr_Gain	= 58
Temp_Get	= 22.8[dC]
X_90_Width	= 11.5[us]
X_Acq_Time	= 0.69206016[s]
X_Angle	= 30[deg]
X_Atn	= 12[dB]
X_Pulse	= 3.83333333[us]
Irr_Atn_Dec	= 21.5[dB]
Irr_Atn_Noe	= 21.5[dB]
Irr_Noise	= WALTZ
Irr_Pwidth	= 76[us]
Decoupling	= TRUE
Initial_Wait	= 1[s]
Noe	= TRUE
Noe_Time	= 2[s]
Repetition_Time	= 2.69206016[s]

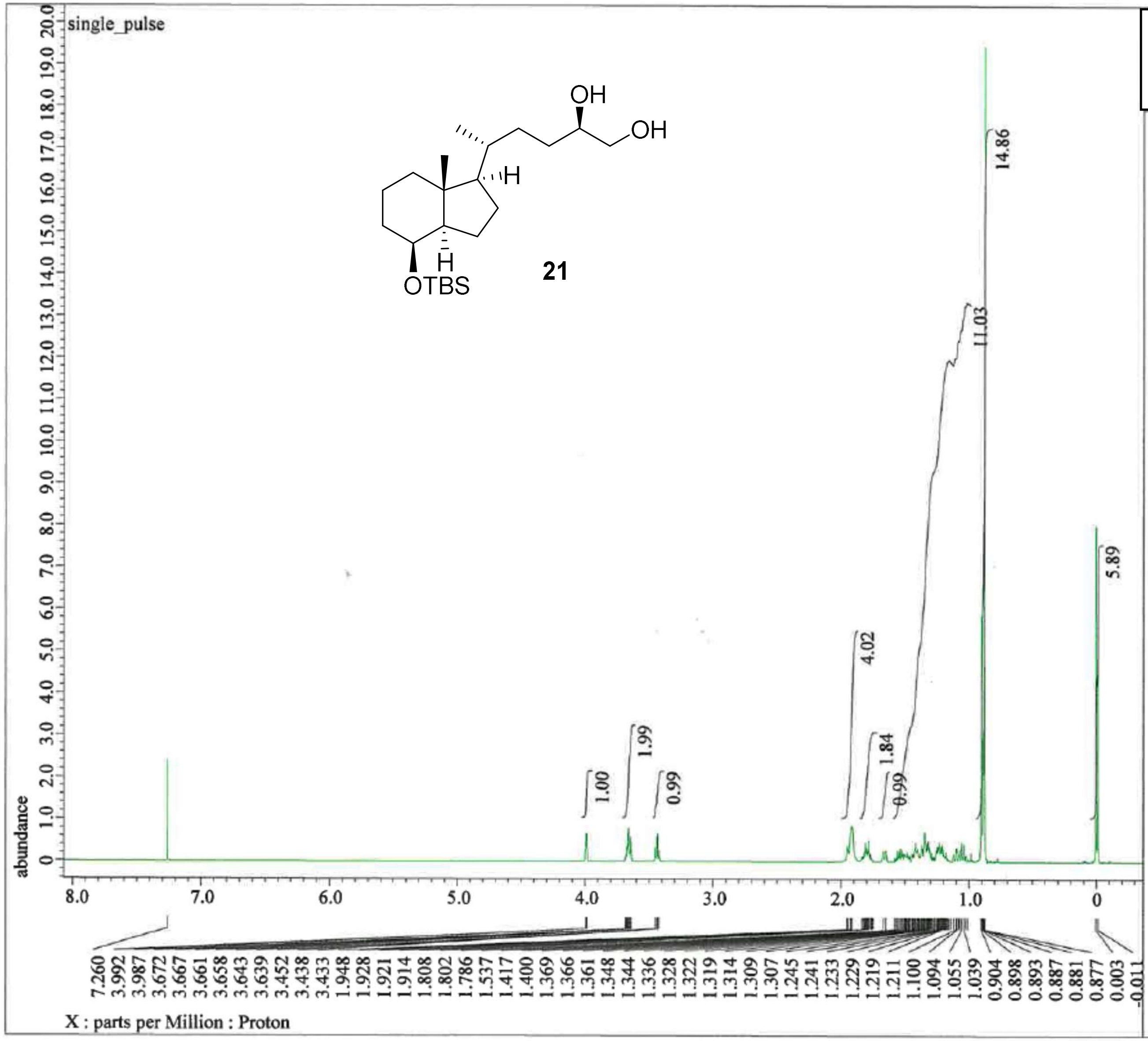

Filename = 41KWG190621-24R-diol_Proto
Author = y-yakka
Experiment = proton.jxp
Sample_Id = 41KWG190621-24R-diol
Solvent = CHLOROFORM-D
Creation_Time = 21-JUN-2019 10:30:47
Revision_Time = 21-JUN-2019 10:47:23
Current_Time = 21-JUN-2019 10:47:37

Comment = single_pulse
Data_Format = 1D COMPLEX
Dim_Size = 13107
Dim_Title = Proton
Dim_Units = [ppm]
Dimensions = X
Site = JNM-ECA600
Spectrometer = DELTA2_NMR

Field_Strength = 14.09636928[T] (600[MHz])
X_Acq_Duration = 1.4548992[s]
X_Domain = 1H
X_Freq = 600.1723046[MHz]
X_Offset = 5[ppm]
X_Points = 16384
X_Prescans = 1
X_Resolution = 0.68733284[Hz]
X_Sweep = 11.26126126[kHz]
X_Sweep_Clipped = 9.00900901[kHz]
Irr_Domain = Proton
Irr_Freq = 600.1723046[MHz]
Irr_Offset = 5[ppm]
Tri_Domain = Proton
Tri_Freq = 600.1723046[MHz]
Tri_Offset = 5[ppm]
Clipped = FALSE
Scans = 8
Total_Scans = 8

Relaxation_Delay = 5[s]
Recvr_Gain = 34
Temp_Get = 23[dC]
X_90_Width = 8.1[us]
X_Acq_Time = 1.4548992[s]
X_Angle = 45[deg]
X_Atn = 2.5[dB]
X_Pulse = 4.05[us]
Irr_Mode = Off
Tri_Mode = Off
Dante_Presat = FALSE
Initial_Wait = 1[s]
Repetition_Time = 6.4548992[s]

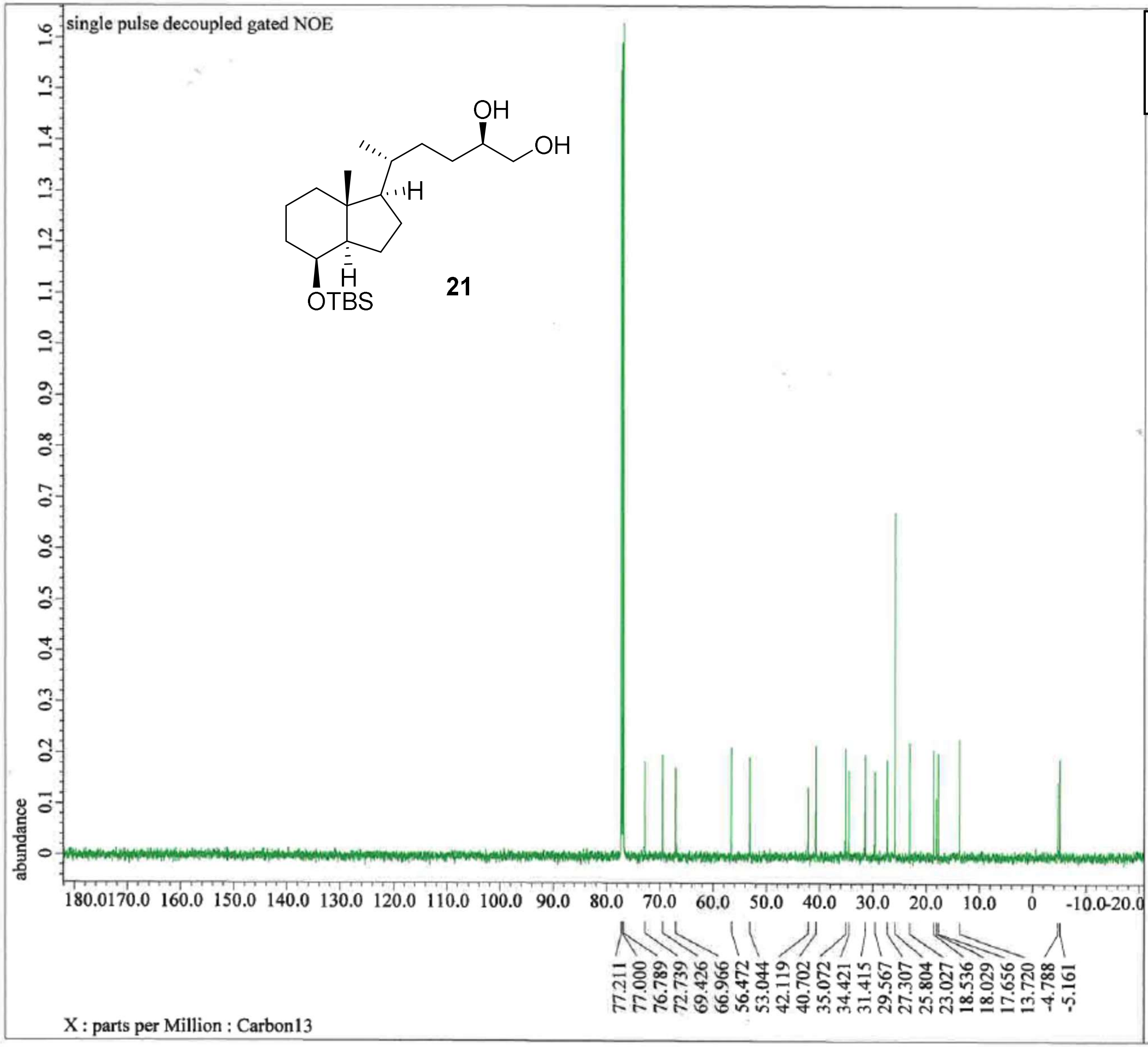

Filename = 41KWG190621-24R-diol_Carbo
Author = y-yakka
Experiment = carbon.jxp
Sample_Id = 41KWG190621-24R-diol
Solvent = CHLOROFORM-D
Creation_Time = 21-JUN-2019 10:34:02
Revision_Time = 21-JUN-2019 11:16:03
Current_Time = 21-JUN-2019 11:18:28

Comment = single pulse decoupled gat
Data_Format = 1D COMPLEX
Dim_Size = 26214
Dim_Title = Carbon13
Dim_Units = [ppm]
Dimensions = X
Site = JNM-ECA600
Spectrometer = DELTA2_NMR

Field_Strength = 14.09636928[T] (600[MHz])
X_Acq_Duration = 0.69206016[s]
X_Domain = 13C
X_Freq = 150.91343039[MHz]
X_Offset = 100[ppm]
X_Points = 32768
X_Prescans = 4
X_Resolution = 1.44496109[Hz]
X_Sweep = 47.34848485[kHz]
X_Sweep_Clipped = 37.87878788[kHz]
Irr_Domain = Proton
Irr_Freq = 600.1723046[MHz]
Irr_Offset = 5[ppm]
Clipped = FALSE
Scans = 300
Total_Scans = 300

Relaxation_Delay = 2[s]
Recvr_Gain = 58
Temp_Get = 23.3[dC]
X_90_Width = 11.5[us]
X_Acq_Time = 0.69206016[s]
X_Angle = 30[deg]
X_Atn = 12[dB]
X_Pulse = 3.83333333[us]
Irr_Atn_Dec = 21.5[dB]
Irr_Atn_Noe = 21.5[dB]
Irr_Noise = WALTZ
Irr_Pwidth = 76[us]
Decoupling = TRUE
Initial_Wait = 1[s]
Noe = TRUE
Noe_Time = 2[s]
Repetition_Time = 2.69206016[s]

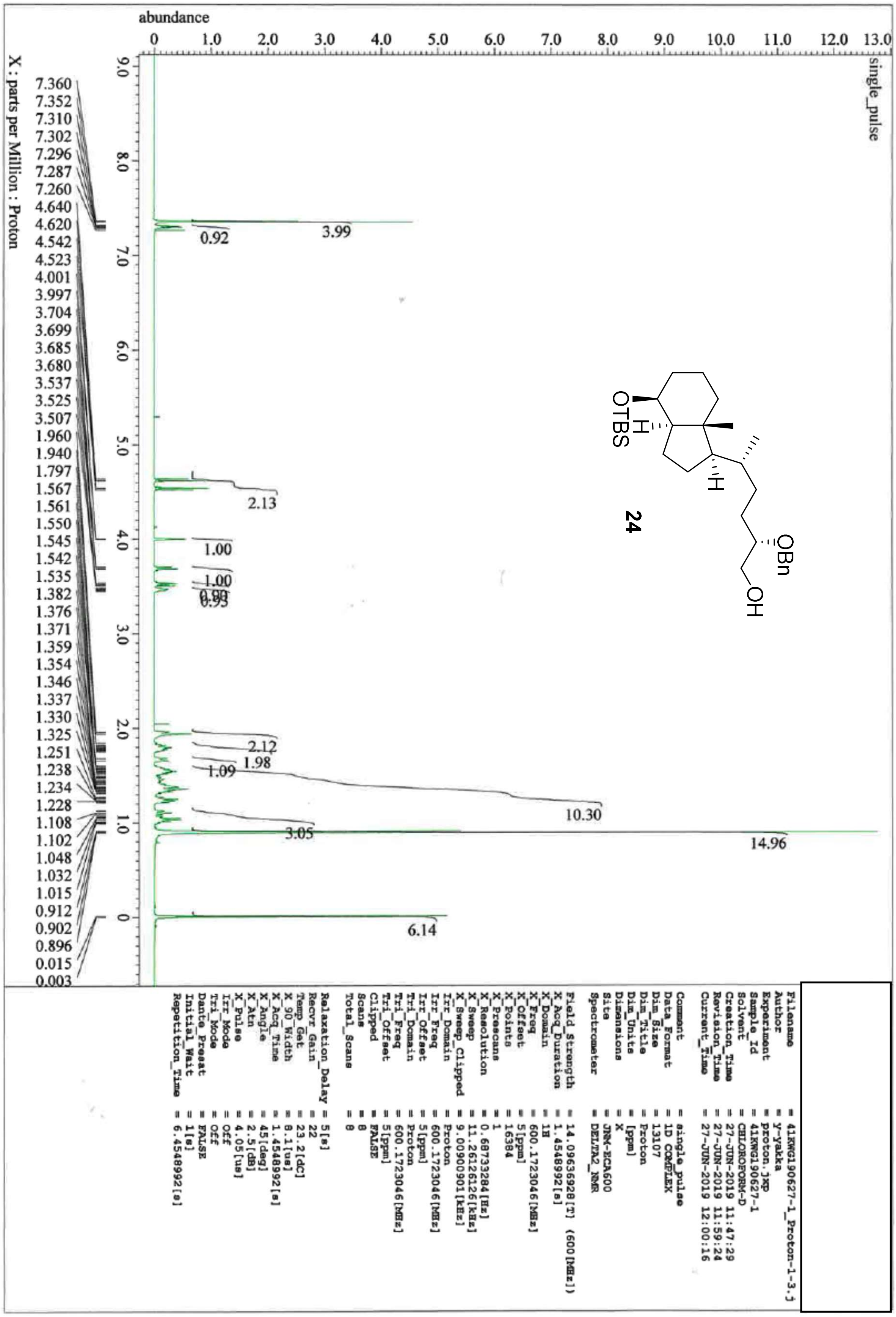
abundance
X : parts per Million : Proton
single_pulse
24
OBn
OH
OTBS
H
Filename = 41KWG190627-1_Proton-1-3.j
Author = y-yaka
Experiment = proton.jxp
Sample_Id = 41KWG190627-1
Solvent = CHLOROFORM-D
Creation_Time = 27-JUN-2019 11:47:29
Revision_Time = 27-JUN-2019 11:59:24
Current_Time = 27-JUN-2019 12:00:16
Comment = single_pulse
Data_Format = 1D COMPLEX
Dim_Size = 13107
Dim_Title = Proton
Dim_Units = [ppm]
Dimensions = X
Site = JNM-ECA600
Spectrometer = DELTA2_NMR
Field_Strength = 14.09636928[T] (600[MHz])
X_Acq_Duration = 1.4548992[s]
X_Domain = 1H
X_Freq = 600.1723046[MHz]
X_Offset = 5[ppm]
X_Points = 16384
X_Prescans = 1
X_Resolution = 0.68733284[Hz]
X_Sweep = 11.26126126[kHz]
X_Sweep_Clipped = 9.00900901[kHz]
Irr_Domain = Proton
Irr_Freq = 600.1723046[MHz]
Irr_Offset = 5[ppm]
Tri_Domain = Proton
Tri_Freq = 600.1723046[MHz]
Tri_Offset = 5[ppm]
Clipped = FALSE
Scans = 8
Total_Scans = 8
Relaxation_Delay = 5[s]
Recvr_Gain = 22
Temp_Get = 23.2[dC]
X_90_Width = 8.1[us]
X_Acq_Time = 1.4548992[s]
X_Angle = 45[deg]
X_Atn = 2.5[dB]
X_Pulse = 4.05[us]
Irr_Mode = Off
Tri_Mode = Off
Dante_Presat = FALSE
Initial_Wait = 1[s]
Repetition_Time = 6.4548992[s]

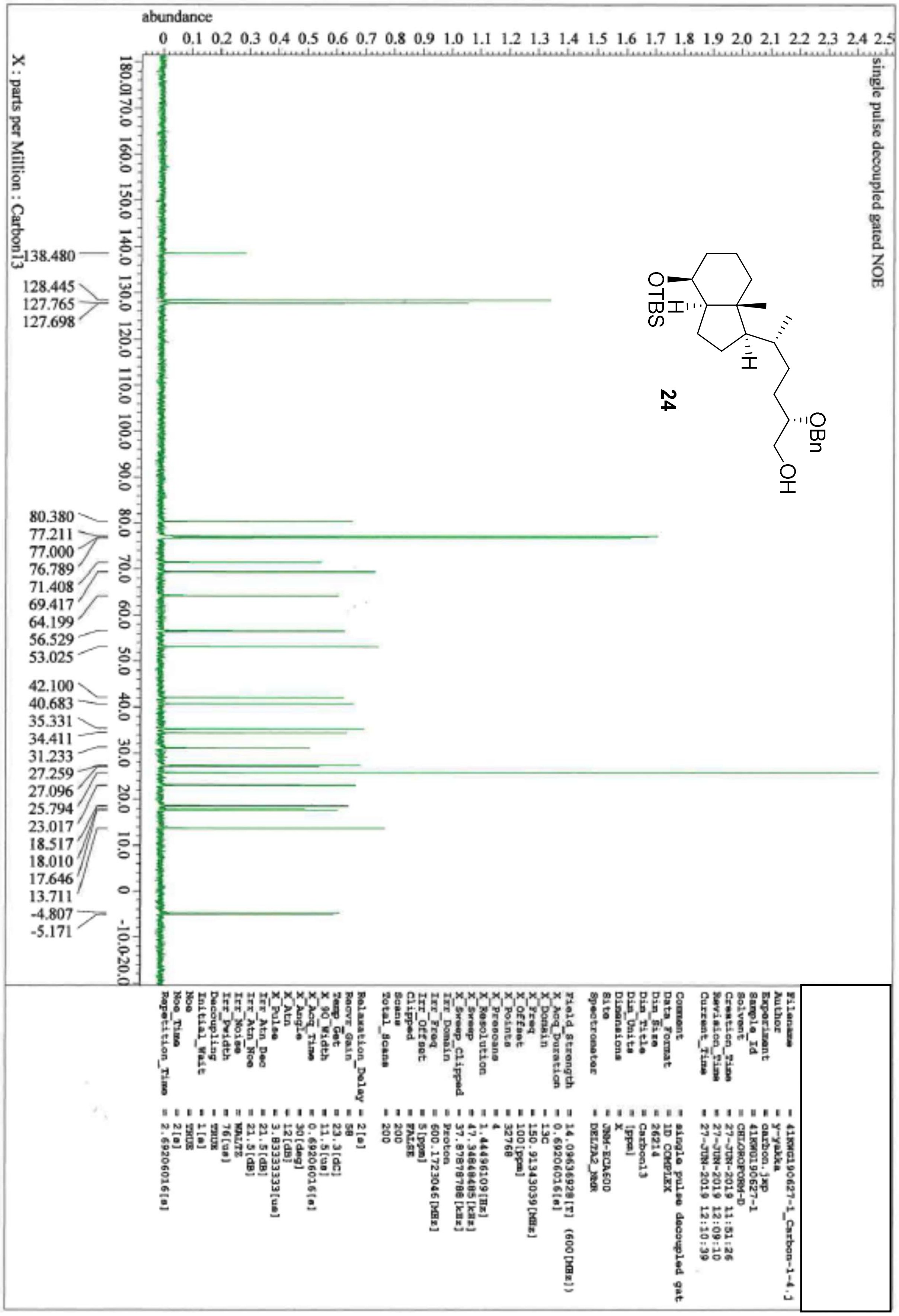
single pulse decoupled gated NOE
abundance
0 0.1 0.2 0.3 0.4 0.5 0.6 0.7 0.8 0.9 1.0 1.1 1.2 1.3 1.4 1.5 1.6 1.7 1.8 1.9 2.0 2.1 2.2 2.3 2.4 2.5
180.0 170.0 160.0 150.0 140.0 130.0 120.0 110.0 100.0 90.0 80.0 70.0 60.0 50.0 40.0 30.0 20.0 10.0 0 -10.0 -20.0
X : parts per Million : Carbon13
138.480
128.445
127.765
127.698
80.380
77.211
77.000
76.789
71.408
69.417
64.199
56.529
53.025
42.100
40.683
35.331
34.411
31.233
27.259
27.096
25.794
23.017
18.517
18.010
17.646
13.711
-4.807
-5.171
OBn
OH
H
H
OTBS
24
Filename = 41KMG190627-1_Carbon-1-4.j
Author = y-yakka
Experiment = carbon.jxp
Sample_Id = 41KMG190627-1
Solvent = CHLOROFORM-D
Creation_Time = 27-JUN-2019 11:51:26
Revision_Time = 27-JUN-2019 12:09:10
Current_Time = 27-JUN-2019 12:10:39
Comment = single pulse decoupled gat
Data_Format = 1D COMPLEX
Dim_Size = 26214
Dim_Title = Carbon13
Dim_Units = [ppm]
Dimensions = X
Site = JNM-ECA600
Spectrometer = DELTA2_NMR
Field_Strength = 14.09636928[T] (600[MHz])
X_Acq_Duration = 0.69206016[s]
X_Domain = 13C
X_Freq = 150.91343039[MHz]
X_Offset = 100[ppm]
X_Points = 32768
X_Prescans = 4
X_Resolution = 1.44496109[Hz]
X_Sweep = 47.34848485[kHz]
X_Sweep_Clipped = 37.87878788[kHz]
Irr_Domain = Proton
Irr_Freq = 600.17230046[MHz]
Irr_Offset = 5[ppm]
Clipped = FALSE
Scans = 200
Total_Scans = 200
Relaxation_Delay = 2[s]
Recvr_Gain = 58
Temp_Get = 23.3[dC]
X_90_Width = 11.5[us]
X_Acq_Time = 0.69206016[s]
X_Angle = 30[deg]
X_Atn = 12[dB]
X_Pulse = 3.83333333[us]
Irr_Atn_Dec = 21.5[dB]
Irr_Atn_Noe = 21.5[dB]
Irr_Noise = WALTZ
Irr_Pwidth = 76[us]
Decoupling = TRUE
Initial_Wait = 1[s]
Noe = TRUE
Noe_Time = 2[s]
Repetition_Time = 2.69206016[s]

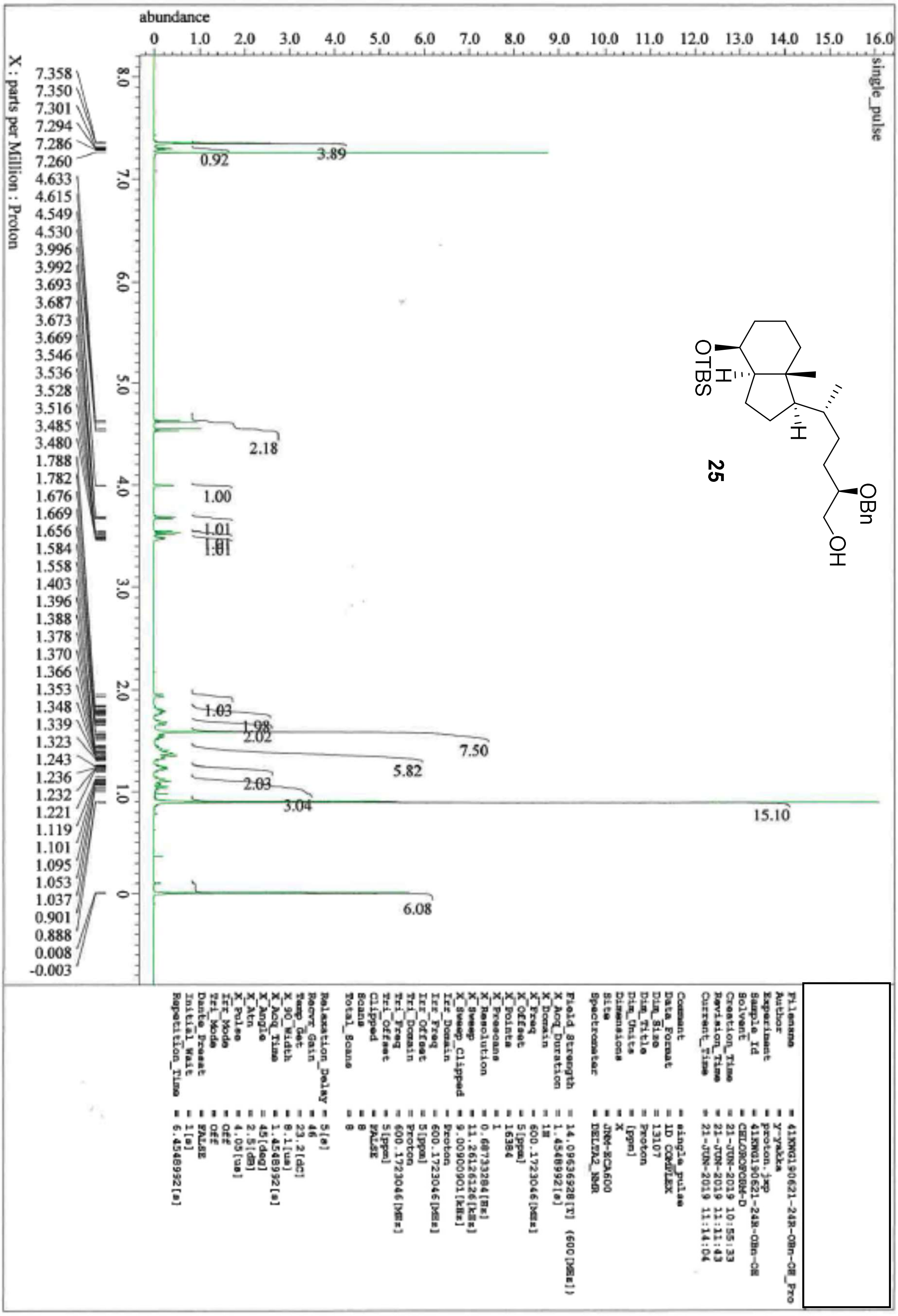
abundance
single_pulse
X : parts per Million : Proton
OTBS
OBn
OH
25

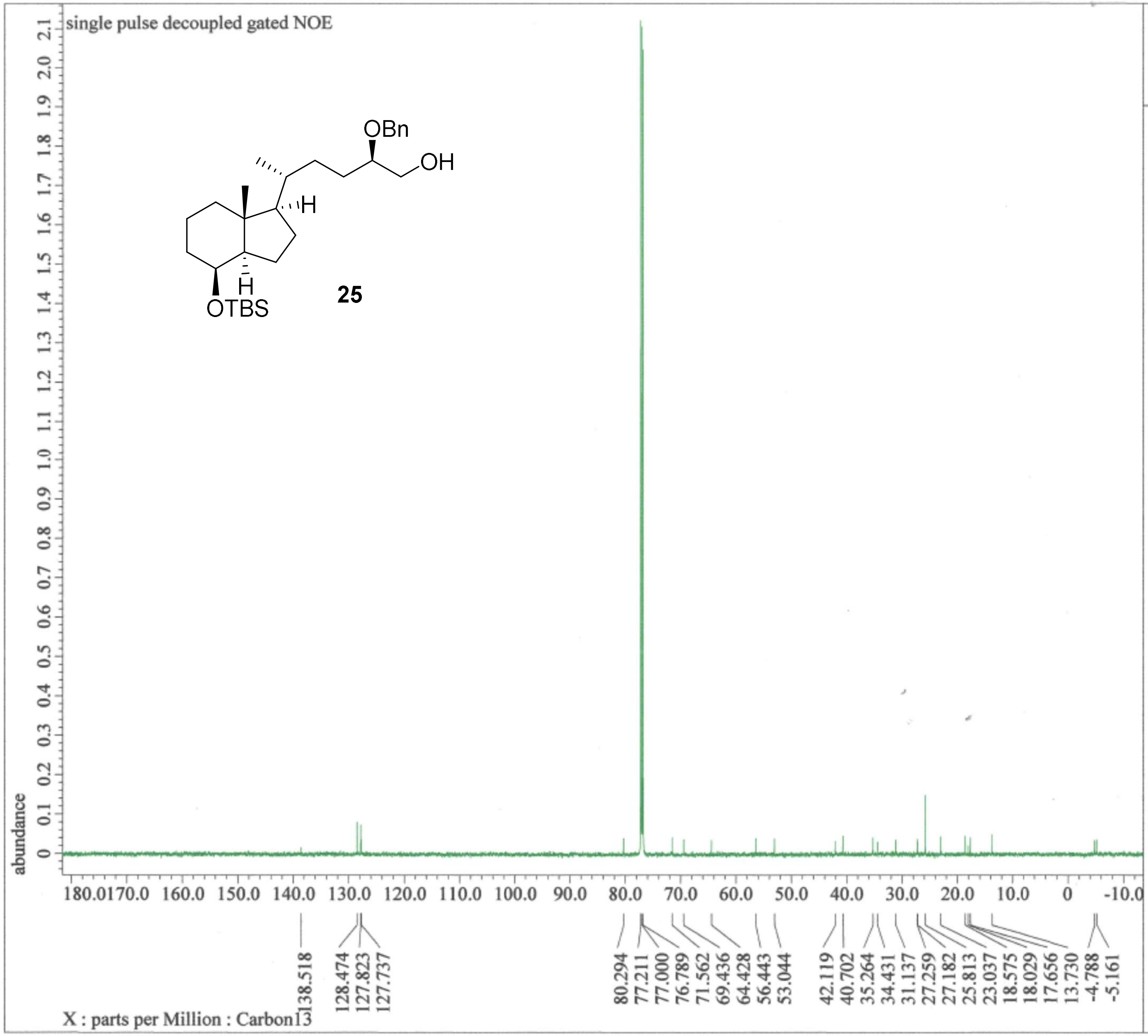

Filename	= 41KWG190621-24R-OBn-OH_Car
Author	= y-yakka
Experiment	= carbon.jxp
Sample_Id	= 41KWG190621-24R-OBn-OH
Solvent	= CHLOROFORM-D
Creation_Time	= 21-JUN-2019 11:22:13
Revision_Time	= 21-JUN-2019 12:32:11
Current_Time	= 21-JUN-2019 12:32:52
Comment	= single pulse decoupled gat
Data_Format	= 1D COMPLEX
Dim_Size	= 26214
Dim_Title	= Carbon13
Dim_Units	= [ppm]
Dimensions	= X
Site	= JNM-ECA600
Spectrometer	= DELTA2_NMR
Field_Strength	= 14.09636928[T] (600[MHz])
X_Acq_Duration	= 0.69206016[s]
X_Domain	= 13C
X_Freq	= 150.91343039[MHz]
X_Offset	= 100[ppm]
X_Points	= 32768
X_Prescans	= 4
X_Resolution	= 1.44496109[Hz]
X_Sweep	= 47.34848485[kHz]
X_Sweep_Clipped	= 37.87878788[kHz]
Irr_Domain	= Proton
Irr_Freq	= 600.1723046[MHz]
Irr_Offset	= 5[ppm]
Clipped	= FALSE
Scans	= 1500
Total_Scans	= 1500
Relaxation_Delay	= 2[s]
Recvr_Gain	= 60
Temp_Get	= 23.4[dC]
X_90_Width	= 11.5[us]
X_Acq_Time	= 0.69206016[s]
X_Angle	= 30[deg]
X_Atn	= 12[dB]
X_Pulse	= 3.83333333[us]
Irr_Atn_Dec	= 21.5[dB]
Irr_Atn_Noe	= 21.5[dB]
Irr_Noise	= WALTZ
Irr_Pwidth	= 76[us]
Decoupling	= TRUE
Initial_Wait	= 1[s]
Noe	= TRUE
Noe_Time	= 2[s]
Repetition_Time	= 2.69206016[s]

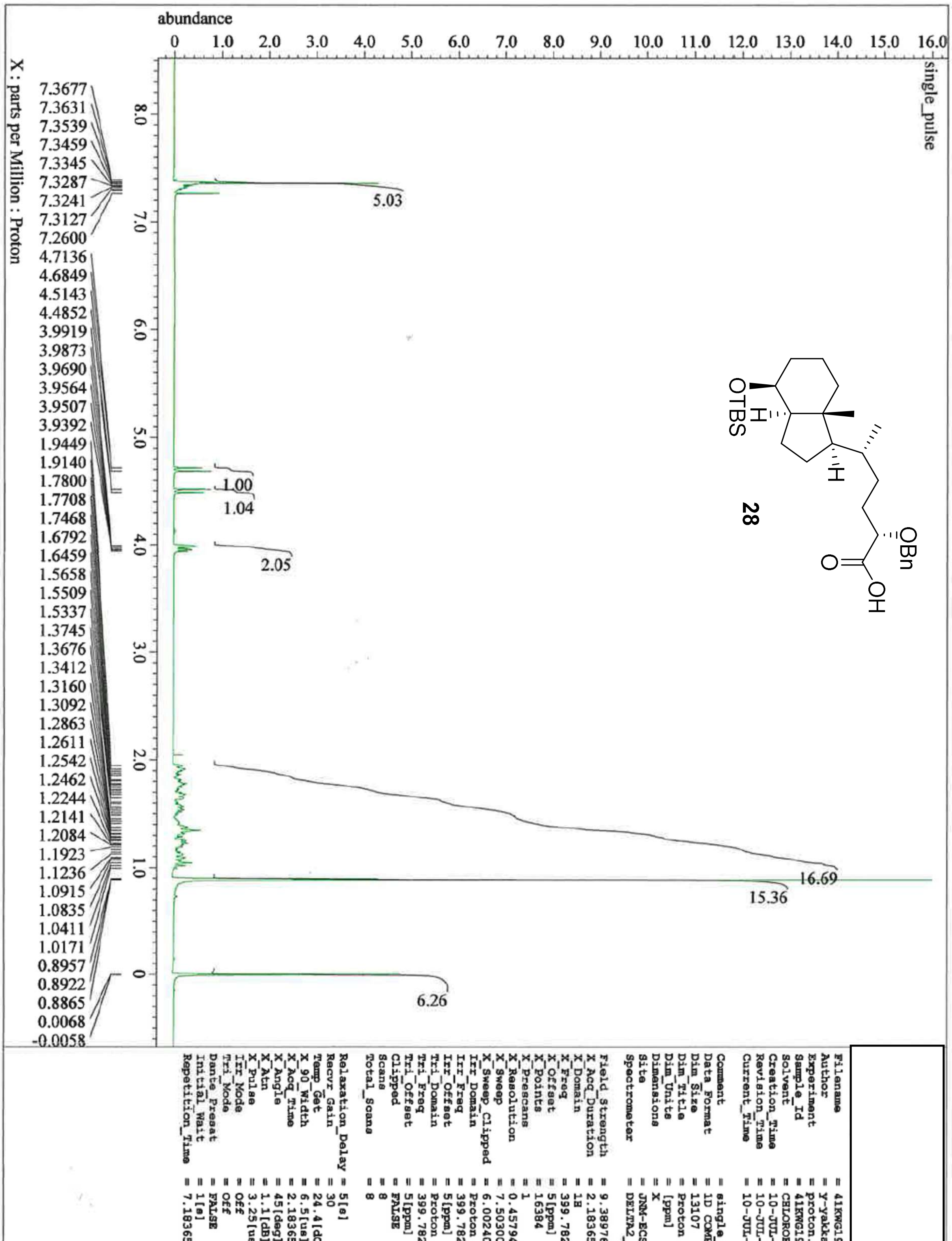
abundance
single_pulse
X : parts per Million : Proton
28
OTBS
OBn
OH
Filename = 41KWG190710-3_Proton-1-5.j
Author = y-yakka
Experiment = proton.jxp
Sample_Id = 41KWG190710-3
Solvent = CHLOROFORM-D
Creation_Time = 10-JUL-2019 14:03:05
Revision_Time = 10-JUL-2019 14:06:38
Current_Time = 10-JUL-2019 14:06:53
Comment = single_pulse
Data_Format = 1D COMPLEX
Dim_Size = 13107
Dim_Title = Proton
Dim_Units = [ppm]
Dimensions = X
Site = JNM-ECS400
Spectrometer = DELTA2_NMR
Field_Strength = 9.389766[T] (400[MHz])
X_Acq_Duration = 2.18365952[s]
X_Domain = 1H
X_Freq = 399.78219838[MHz]
X_Offset = 5[ppm]
X_Points = 16384
X_Prescans = 1
X_Resolution = 0.45794685[Hz]
X_Sweep = 7.5030012[kHz]
X_Sweep_Clipped = 6.00240096[kHz]
Irr_Domain = Proton
Irr_Freq = 399.78219838[MHz]
Irr_Offset = 5[ppm]
Tri_Domain = Proton
Tri_Freq = 399.78219838[MHz]
Tri_Offset = 5[ppm]
Clipped = FALSE
Scans = 8
Total_Scans = 8
Relaxation_Delay = 5[s]
Recvr_Gain = 30
Temp_Get = 24.4[dC]
X_90_Width = 6.5[us]
X_Acq_Time = 2.18365952[s]
X_Angle = 45[deg]
X_Atn = 1.1[dB]
X_Pulse = 3.25[us]
Irr_Mode = Off
Tri_Mode = Off
Dante_Presat = FALSE
Initial_Wait = 1[s]
Repetition_Time = 7.18365952[s]

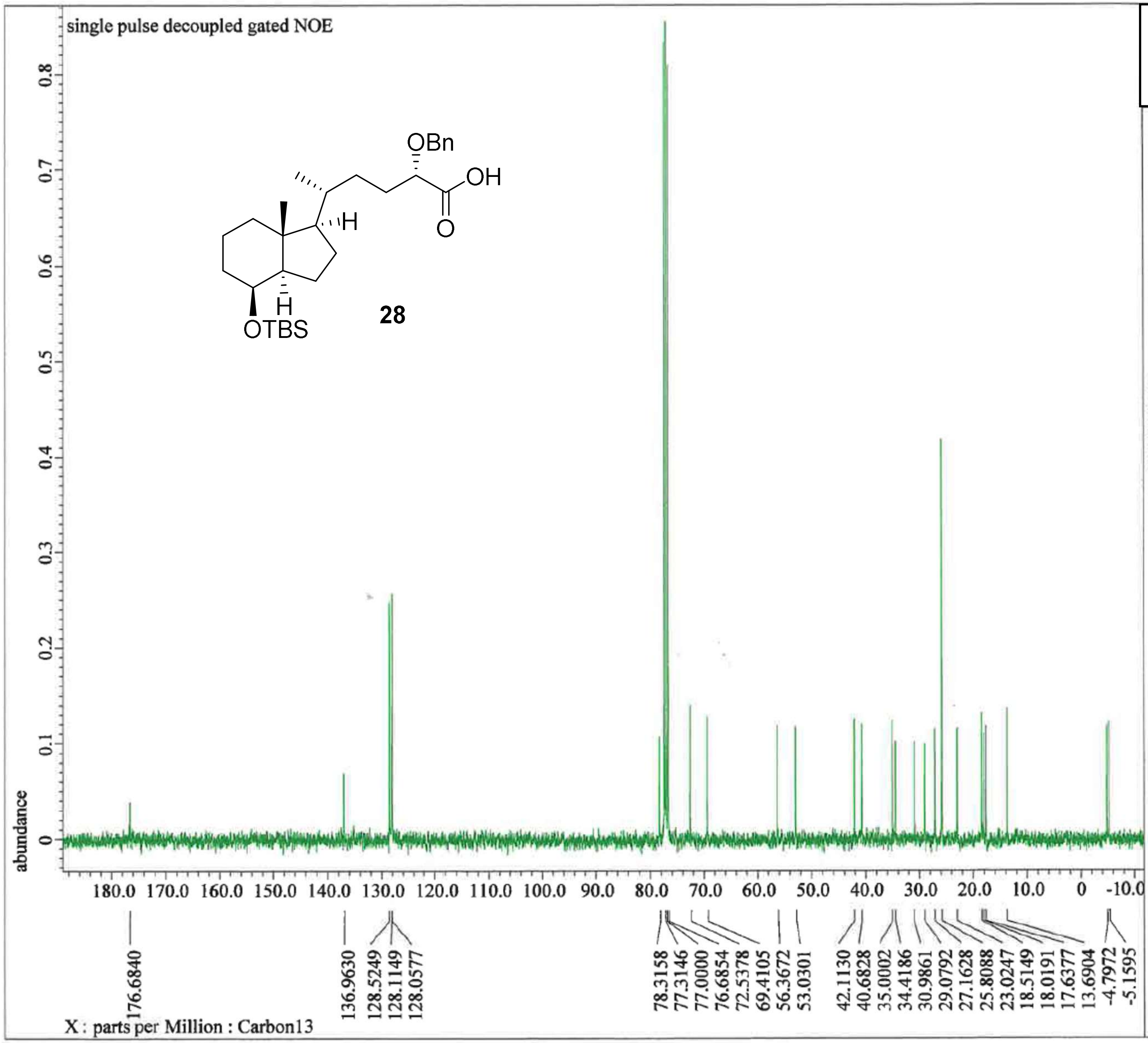

Filename	= 41KWG190710-3_Carbon_ft-1-
Author	= y-yakka
Experiment	= carbon.jxp
Sample_Id	= 41KWG190710-3
Solvent	= CHLOROFORM-D
Creation_Time	= 10-JUL-2019 14:06:52
Revision_Time	= 11-JUL-2019 19:33:48
Current_Time	= 11-JUL-2019 19:34:26
Comment	= single pulse decoupled gat
Data_Format	= 1D COMPLEX
Dim_Size	= 26214
Dim_Title	= Carbon13
Dim_Units	= [ppm]
Dimensions	= X
Site	= JNM-ECS400
Spectrometer	= DELTA2_NMR
Field_Strength	= 9.389766[T] (400[MHz])
X_Acq_Duration	= 1.04333312[s]
X_Domain	= 13C
X_Freq	= 100.52530333[MHz]
X_Offset	= 100[ppm]
X_Points	= 32768
X_Prescans	= 4
X_Resolution	= 0.95846665[Hz]
X_Sweep	= 31.40703518[kHz]
X_Sweep_Clipped	= 25.12562814[kHz]
Irr_Domain	= Proton
Irr_Freq	= 399.78219838[MHz]
Irr_Offset	= 5[ppm]
Clipped	= FALSE
Scans	= 256
Total_Scans	= 256
Relaxation_Delay	= 2[s]
Recvr_Gain	= 60
Temp_Get	= 24.4[dC]
X_90_Width	= 9.7[us]
X_Acq_Time	= 1.04333312[s]
X_Angle	= 30[deg]
X_Atn	= 3[dB]
X_Pulse	= 3.23333333[us]
Irr_Atn_Dec	= 25.6[dB]
Irr_Atn_Noe	= 25.6[dB]
Irr_Noise	= WALTZ
Irr_Pwidth	= 0.115[ms]
Decoupling	= TRUE
Initial_Wait	= 1[s]
Noe	= TRUE
Noe_Time	= 2[s]
Repetition_Time	= 3.04333312[s]

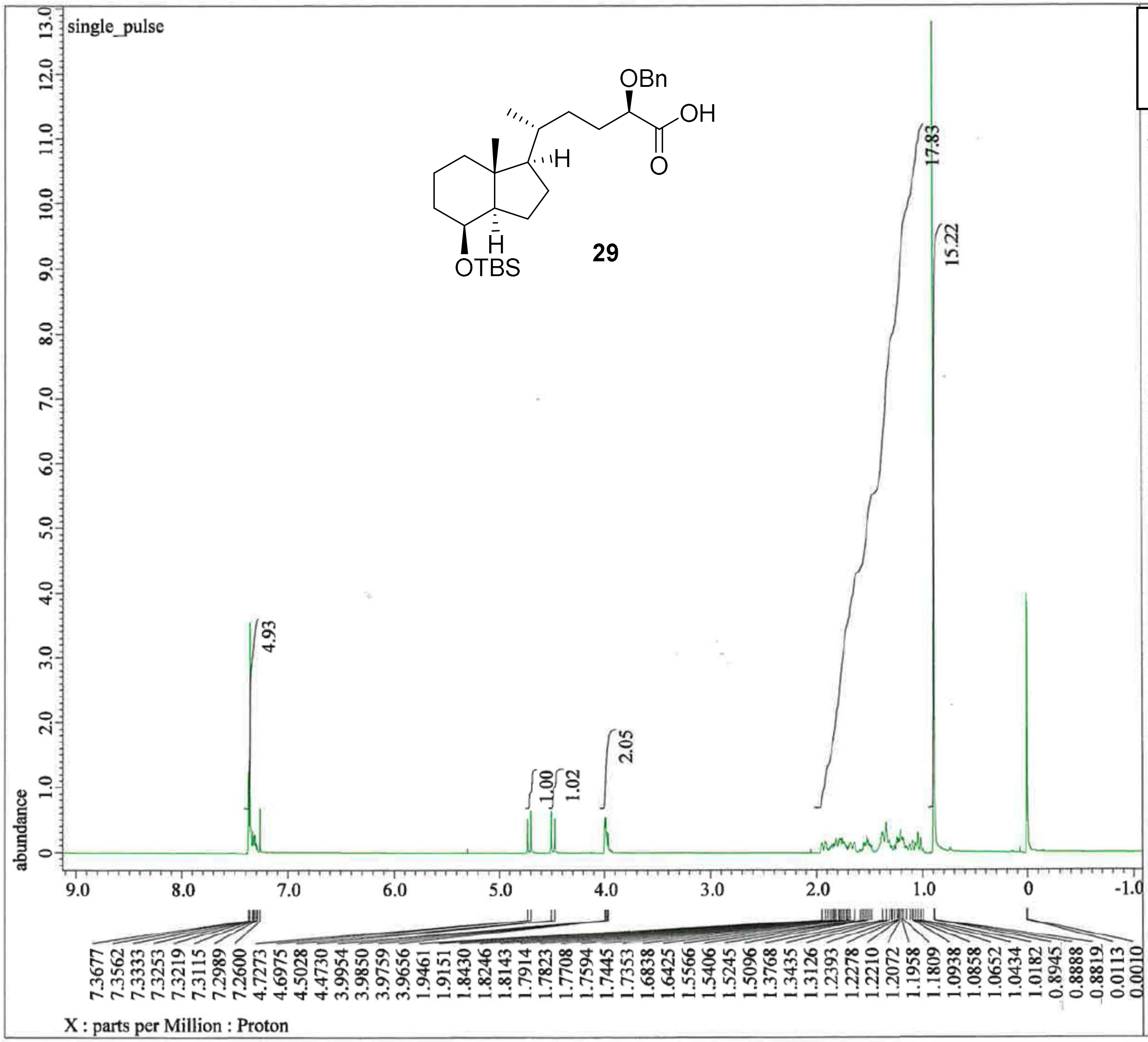


```
Filename           = 41KWG190711-3_Proton_copy1
Author             = y-yakka
Experiment         = proton.jxp
Sample_Id          = 41KWG190711-3
Solvent            = CHLOROFORM-D
Creation_Time      = 11-JUL-2019 20:43:36
Revision_Time      = 11-JUL-2019 20:46:22
Current_Time       = 11-JUL-2019 20:46:54

Comment            = single_pulse
Data_Format        = 1D COMPLEX
Dim_Size           = 13107
Dim_Title          = Proton
Dim_Units          = [ppm]
Dimensions         = X
Site               = JNM-ECS400
Spectrometer       = DELTA2_NMR

Field_Strength     = 9.389766[T] (400[MHz])
X_Acq_Duration     = 0[s]
X_Domain           = 1H
X_Freq             = 399.78219838[MHz]
X_Offset           = 5[ppm]
X_Points           = 16384
X_Prescans         = 1
X_Resolution       = 0.45794685[Hz]
X_Sweep            = 7.5030012[kHz]
X_Sweep_Clipped    = 6.00240096[kHz]
Irr_Domain         = Proton
Irr_Freq           = 399.78219838[MHz]
Irr_Offset         = 5[ppm]
Tri_Domain         = Proton
Tri_Freq           = 399.78219838[MHz]
Tri_Offset         = 5[ppm]
Clipped            = FALSE
Incomplete_Copy    = TRUE
Scans              = 2
Total_Scans        = 2

Relaxation_Delay   = 5[s]
Recvr_Gain         = 26
Temp_Get           = 24.4[dC]
X_90_Width         = 6.5[us]
X_Acq_Time         = 2.18365952[s]
X_Angle            = 45[deg]
X_Atn              = 1.1[dB]
X_Pulse            = 3.25[us]
Irr_Mode           = Off
Tri_Mode           = Off
Dante_Presat       = FALSE
Initial_Wait       = 1[s]
Repetition_Time    = 7.18365952[s]
```

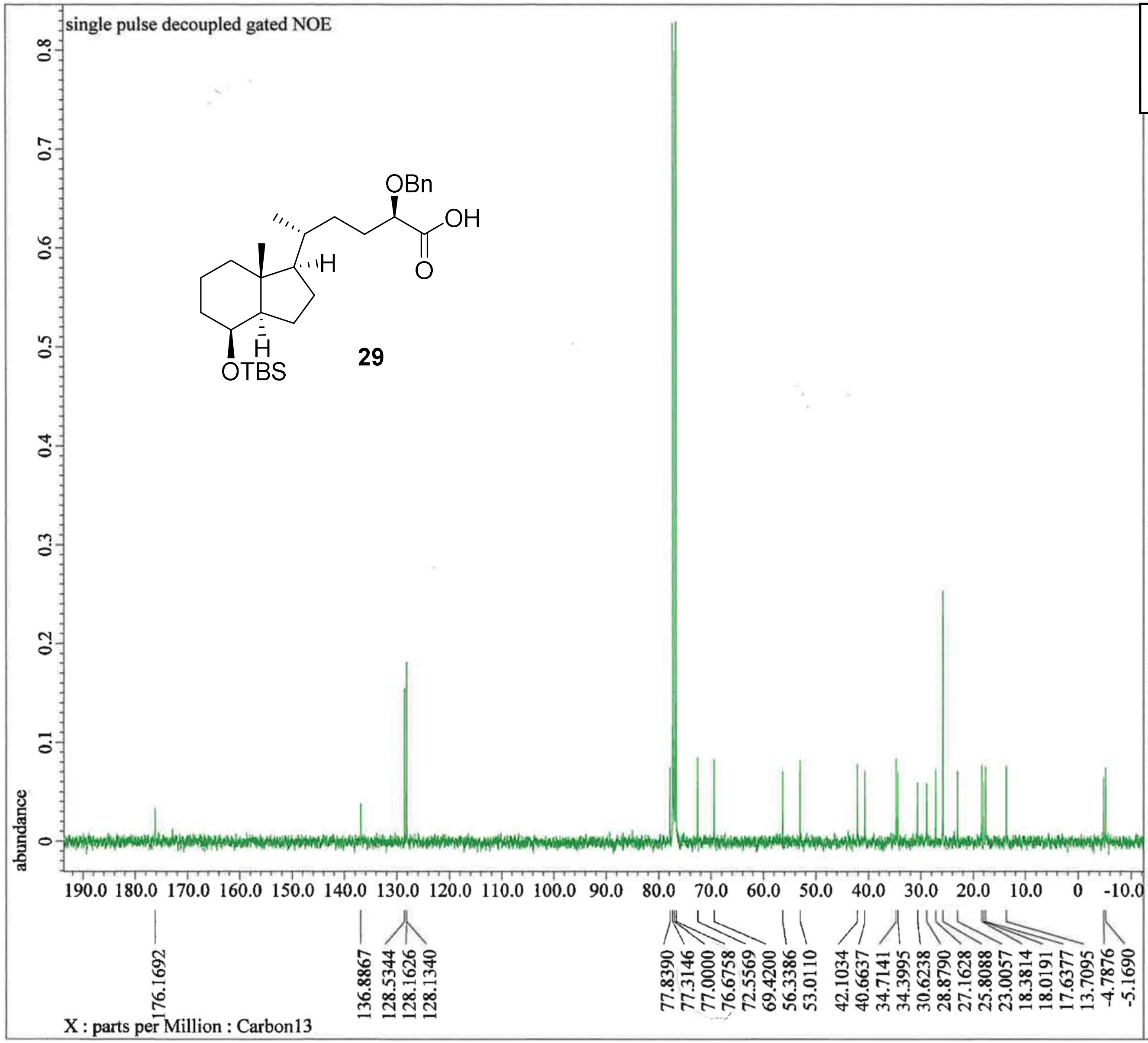

Filename	= 41KWG190711-2_Carbon_copy4
Author	= y-yakka
Experiment	= carbon.jxp
Sample_Id	= 41KWG190711-2
Solvent	= CHLOROFORM-D
Creation_Time	= 11-JUL-2019 19:15:39
Revision_Time	= 11-JUL-2019 19:36:23
Current_Time	= 11-JUL-2019 19:40:05
Comment	= single pulse decoupled gat
Data_Format	= 1D COMPLEX
Dim_Size	= 26214
Dim_Title	= Carbon13
Dim_Units	= [ppm]
Dimensions	= X
Site	= JNM-ECS400
Spectrometer	= DELTA2_NMR
Field_Strength	= 9.389766[T] (400[MHz])
X_Acq_Duration	= 0[s]
X_Domain	= 13C
X_Freq	= 100.52530333[MHz]
X_Offset	= 100[ppm]
X_Points	= 32768
X_Prescans	= 4
X_Resolution	= 0.95846665[Hz]
X_Sweep	= 31.40703518[kHz]
X_Sweep_Clipped	= 25.12562814[kHz]
Irr_Domain	= Proton
Irr_Freq	= 399.78219838[MHz]
Irr_Offset	= 5[ppm]
Clipped	= FALSE
Incomplete_Copy	= TRUE
Scans	= 381
Total_Scans	= 381
Relaxation_Delay	= 2[s]
Recvr_Gain	= 60
Temp_Get	= 24.3[dC]
X_90_Width	= 9.7[us]
X_Acq_Time	= 1.04333312[s]
X_Angle	= 30[deg]
X_Atn	= 3[dB]
X_Pulse	= 3.23333333[us]
Irr_Atn_Dec	= 25.6[dB]
Irr_Atn_Noe	= 25.6[dB]
Irr_Noise	= WALTZ
Irr_Pwidth	= 0.115[ms]
Decoupling	= TRUE
Initial_Wait	= 1[s]
Noe	= TRUE
Noe_Time	= 2[s]
Repetition_Time	= 3.04333312[s]

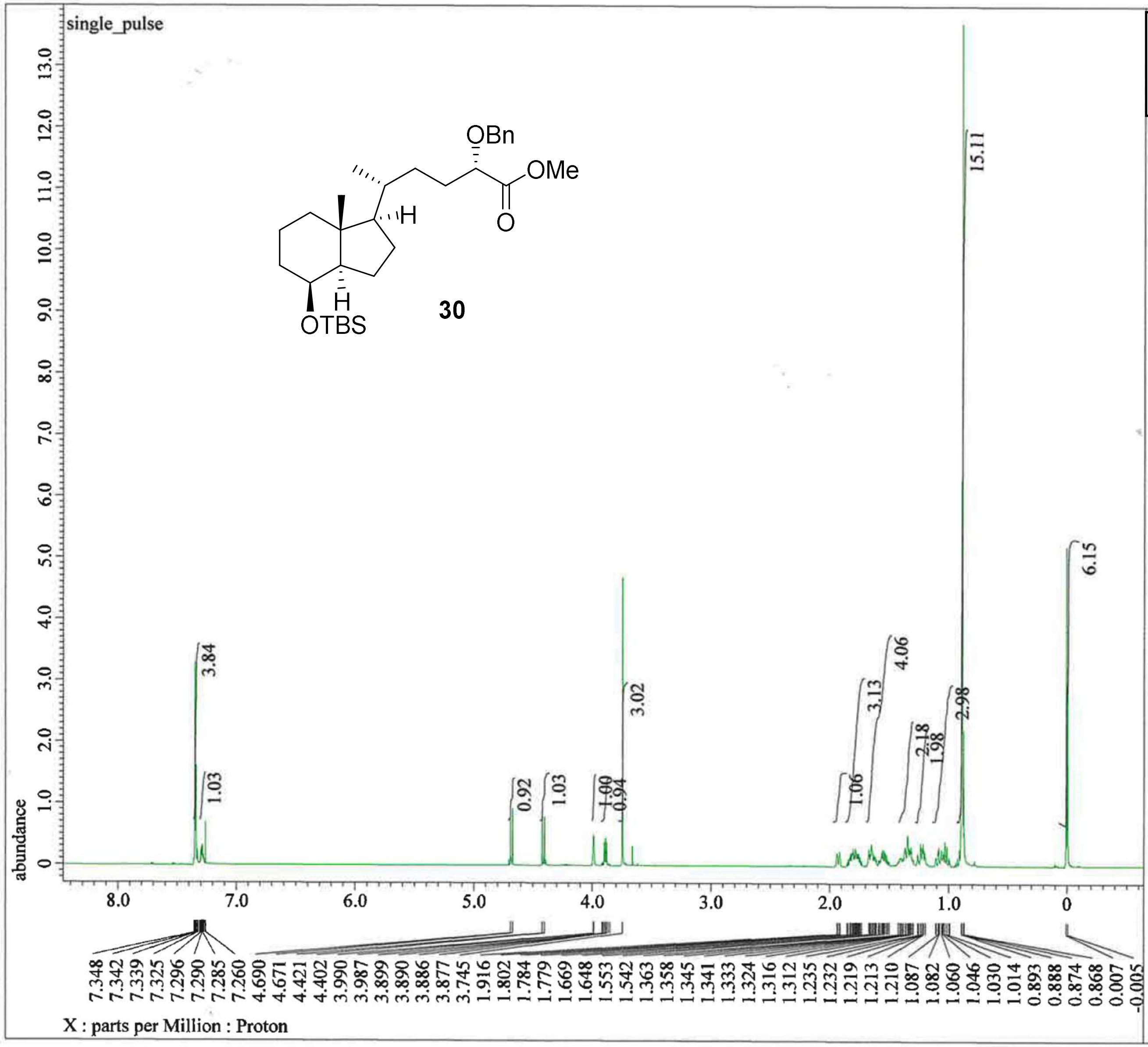

Filename	= 41KWG190624-24SOBn-COOMe_P
Author	= y-yakka
Experiment	= proton.jxp
Sample_Id	= 41KWG190624-24SOBn-COOMe
Solvent	= CHLOROFORM-D
Creation_Time	= 24-JUN-2019 17:02:40
Revision_Time	= 24-JUN-2019 17:15:27
Current_Time	= 24-JUN-2019 17:16:44
Comment	= single_pulse
Data_Format	= 1D COMPLEX
Dim_Size	= 13107
Dim_Title	= Proton
Dim_Units	= [ppm]
Dimensions	= X
Site	= JNM-ECA600
Spectrometer	= DELTA2_NMR
Field_Strength	= 14.09636928[T] (600[MHz])
X_Acq_Duration	= 1.4548992[s]
X_Domain	= 1H
X_Freq	= 600.1723046[MHz]
X_Offset	= 5[ppm]
X_Points	= 16384
X_Prescans	= 1
X_Resolution	= 0.68733284[Hz]
X_Sweep	= 11.26126126[kHz]
X_Sweep_Clipped	= 9.00900901[kHz]
Irr_Domain	= Proton
Irr_Freq	= 600.1723046[MHz]
Irr_Offset	= 5[ppm]
Tri_Domain	= Proton
Tri_Freq	= 600.1723046[MHz]
Tri_Offset	= 5[ppm]
Clipped	= FALSE
Scans	= 8
Total_Scans	= 8
Relaxation_Delay	= 5[s]
Recvr_Gain	= 22
Temp_Get	= 23.1[dC]
X_90_Width	= 8.1[us]
X_Acq_Time	= 1.4548992[s]
X_Angle	= 45[deg]
X_Atn	= 2.5[dB]
X_Pulse	= 4.05[us]
Irr_Mode	= Off
Tri_Mode	= Off
Dante_Presat	= FALSE
Initial_Wait	= 1[s]
Repetition_Time	= 6.4548992[s]

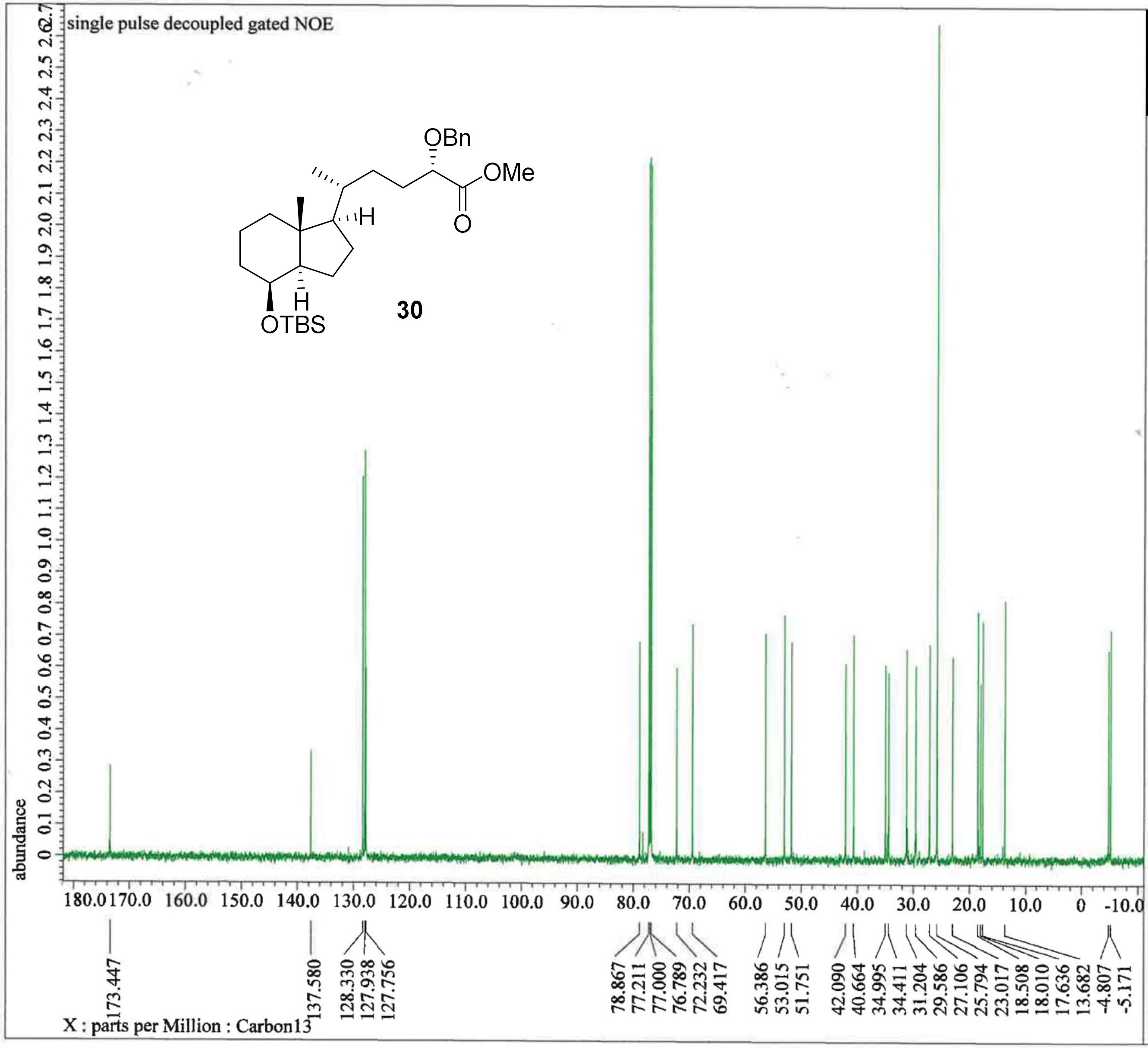

Filename	= 41KWG190624-24SOBn-COOMe_C
Author	= y-yakka
Experiment	= carbon.jxp
Sample_Id	= 41KWG190624-24SOBn-COOMe
Solvent	= CHLOROFORM-D
Creation_Time	= 24-JUN-2019 17:06:32
Revision_Time	= 23-JAN-2020 17:25:28
Current_Time	= 23-JAN-2020 17:28:03
Comment	= single pulse decoupled gat
Data_Format	= 1D COMPLEX
Dim_Size	= 26214
Dim_Title	= Carbon13
Dim_Units	= [ppm]
Dimensions	= X
Site	= JNM-ECA600
Spectrometer	= DELTA2_NMR
Field_Strength	= 14.09636928[T] (600[MHz])
X_Acq_Duration	= 0.69206016[s]
X_Domain	= 13C
X_Freq	= 150.91343039[MHz]
X_Offset	= 100[ppm]
X_Points	= 32768
X_Prescans	= 4
X_Resolution	= 1.44496109[Hz]
X_Sweep	= 47.34848485[kHz]
X_Sweep_Clipped	= 37.87878788[kHz]
Irr_Domain	= Proton
Irr_Freq	= 600.1723046[MHz]
Irr_Offset	= 5[ppm]
Clipped	= FALSE
Scans	= 256
Total_Scans	= 256
Relaxation_Delay	= 2[s]
Recvr_Gain	= 60
Temp_Get	= 23.3[dC]
X_90_Width	= 11.5[us]
X_Acq_Time	= 0.69206016[s]
X_Angle	= 30[deg]
X_Atn	= 12[dB]
X_Pulse	= 3.83333333[us]
Irr_Atn_Dec	= 21.5[dB]
Irr_Atn_Noe	= 21.5[dB]
Irr_Noise	= WALTZ
Irr_Pwidth	= 76[us]
Decoupling	= TRUE
Initial_Wait	= 1[s]
Noe	= TRUE
Noe_Time	= 2[s]
Repetition_Time	= 2.69206016[s]

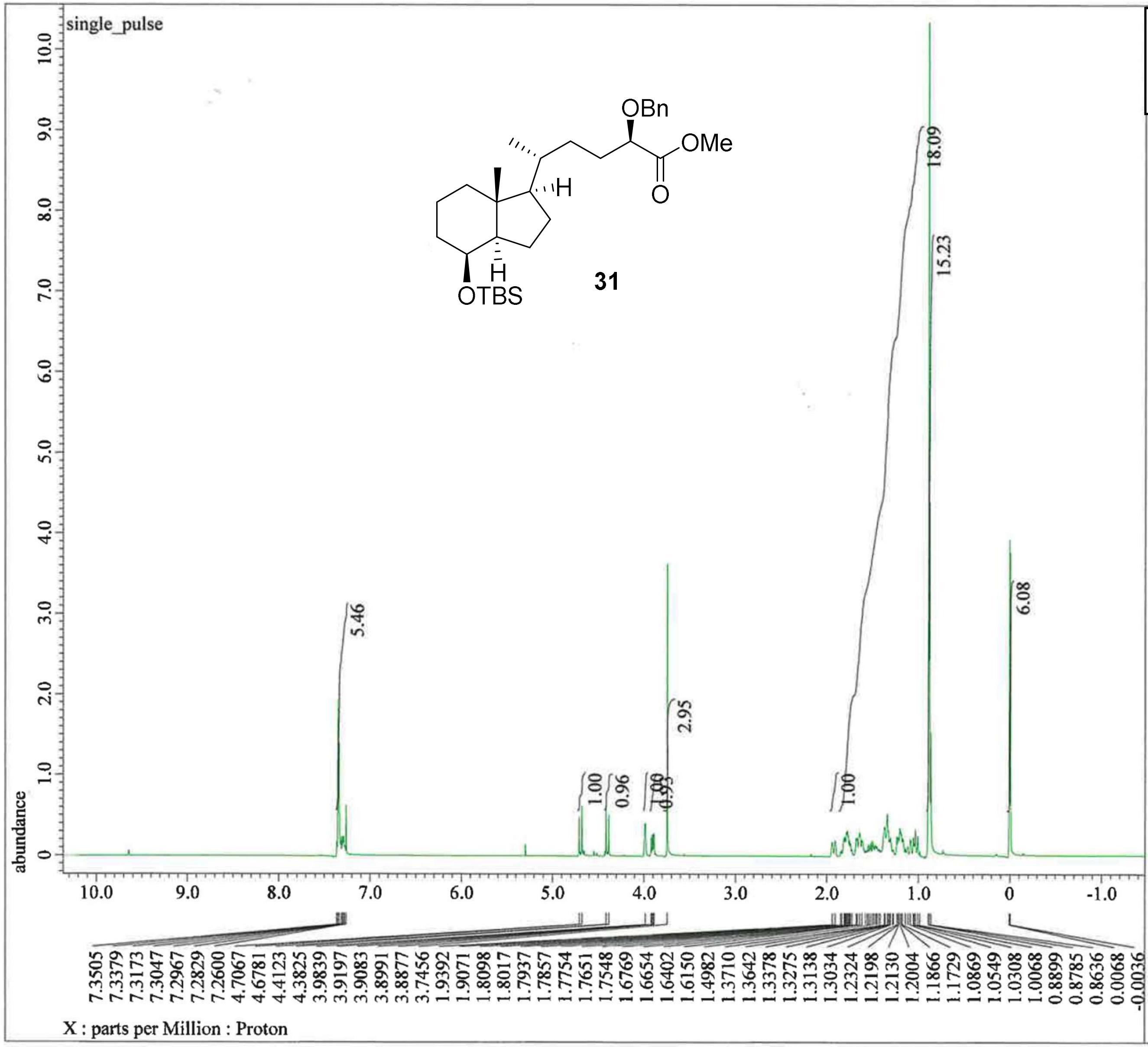


```
Filename          = 41KWG201208-3_Proton-2-3.j
Author            = y-yakka
Experiment        = proton.jxp
Sample_Id         = 41KWG201208-3
Solvent           = CHLOROFORM-D
Creation_Time     =  8-DEC-2020 16:01:19
Revision_Time     =  8-DEC-2020 16:16:00
Current_Time      =  8-DEC-2020 16:16:10

Comment           = single_pulse
Data_Format       = 1D COMPLEX
Dim_Size          = 13107
Dim_Title         = Proton
Dim_Units         = [ppm]
Dimensions        = X
Site              = JNM-ECS400
Spectrometer      = DELTA2_NMR

Field_Strength    = 9.389766[T] (400[MHz])
X_Acq_Duration    = 2.18365952[s]
X_Domain          = 1H
X_Freq            = 399.78219838[MHz]
X_Offset          = 5[ppm]
X_Points          = 16384
X_Prescans        = 1
X_Resolution      = 0.45794685[Hz]
X_Sweep           = 7.5030012[kHz]
X_Sweep_Clipped   = 6.00240096[kHz]
Irr_Domain        = Proton
Irr_Freq          = 399.78219838[MHz]
Irr_Offset        = 5[ppm]
Tri_Domain        = Proton
Tri_Freq          = 399.78219838[MHz]
Tri_Offset        = 5[ppm]
Clipped           = FALSE
Scans             = 8
Total_Scans       = 8

Relaxation_Delay  = 5[s]
Recvr_Gain        = 26
Temp_Get          = 23.9[dC]
X_90_Width        = 6.7[us]
X_Acq_Time        = 2.18365952[s]
X_Angle           = 45[deg]
X_Atn             = 1.1[dB]
X_Pulse           = 3.35[us]
Irr_Mode          = Off
Tri_Mode          = Off
Dante_Presat      = FALSE
Initial_Wait      = 1[s]
Repetition_Time   = 7.18365952[s]
```

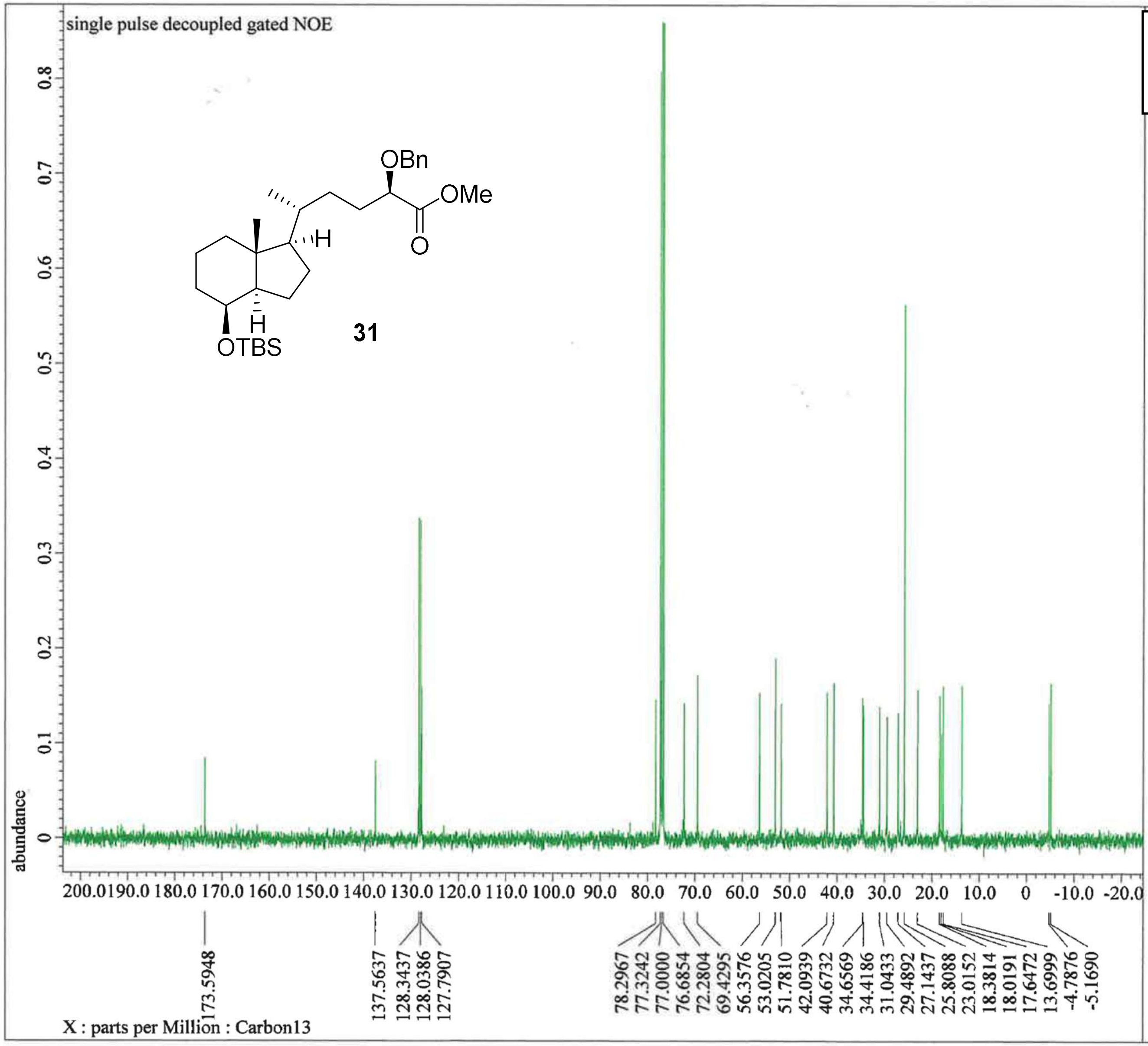

Filename	= 41KWG201208-3_Carbon-1-3.j
Author	= y-yakka
Experiment	= carbon.jxp
Sample_Id	= 41KWG201208-3
Solvent	= CHLOROFORM-D
Creation_Time	= 8-DEC-2020 15:46:07
Revision_Time	= 8-DEC-2020 16:00:09
Current_Time	= 8-DEC-2020 16:03:57
Comment	= single pulse decoupled gat
Data_Format	= 1D COMPLEX
Dim_Size	= 26214
Dim_Title	= Carbon13
Dim_Units	= [ppm]
Dimensions	= X
Site	= JNM-ECS400
Spectrometer	= DELTA2_NMR
Field_Strength	= 9.389766[T] (400[MHz])
X_Acq_Duration	= 1.04333312[s]
X_Domain	= 13C
X_Freq	= 100.52530333[MHz]
X_Offset	= 100[ppm]
X_Points	= 32768
X_Prescans	= 4
X_Resolution	= 0.95846665[Hz]
X_Sweep	= 31.40703518[kHz]
X_Sweep_Clipped	= 25.12562814[kHz]
Irr_Domain	= Proton
Irr_Freq	= 399.78219838[MHz]
Irr_Offset	= 5[ppm]
Clipped	= FALSE
Scans	= 256
Total_Scans	= 256
Relaxation_Delay	= 2[s]
Recvr_Gain	= 60
Temp_Get	= 23.9[dC]
X_90_Width	= 9.9[us]
X_Acq_Time	= 1.04333312[s]
X_Angle	= 30[deg]
X_Atn	= 3[dB]
X_Pulse	= 3.3[us]
Irr_Atn_Dec	= 25.6[dB]
Irr_Atn_Noe	= 25.6[dB]
Irr_Noise	= WALTZ
Irr_Pwidth	= 0.115[ms]
Decoupling	= TRUE
Initial_Wait	= 1[s]
Noe	= TRUE
Noe_Time	= 2[s]
Repetition_Time	= 3.04333312[s]

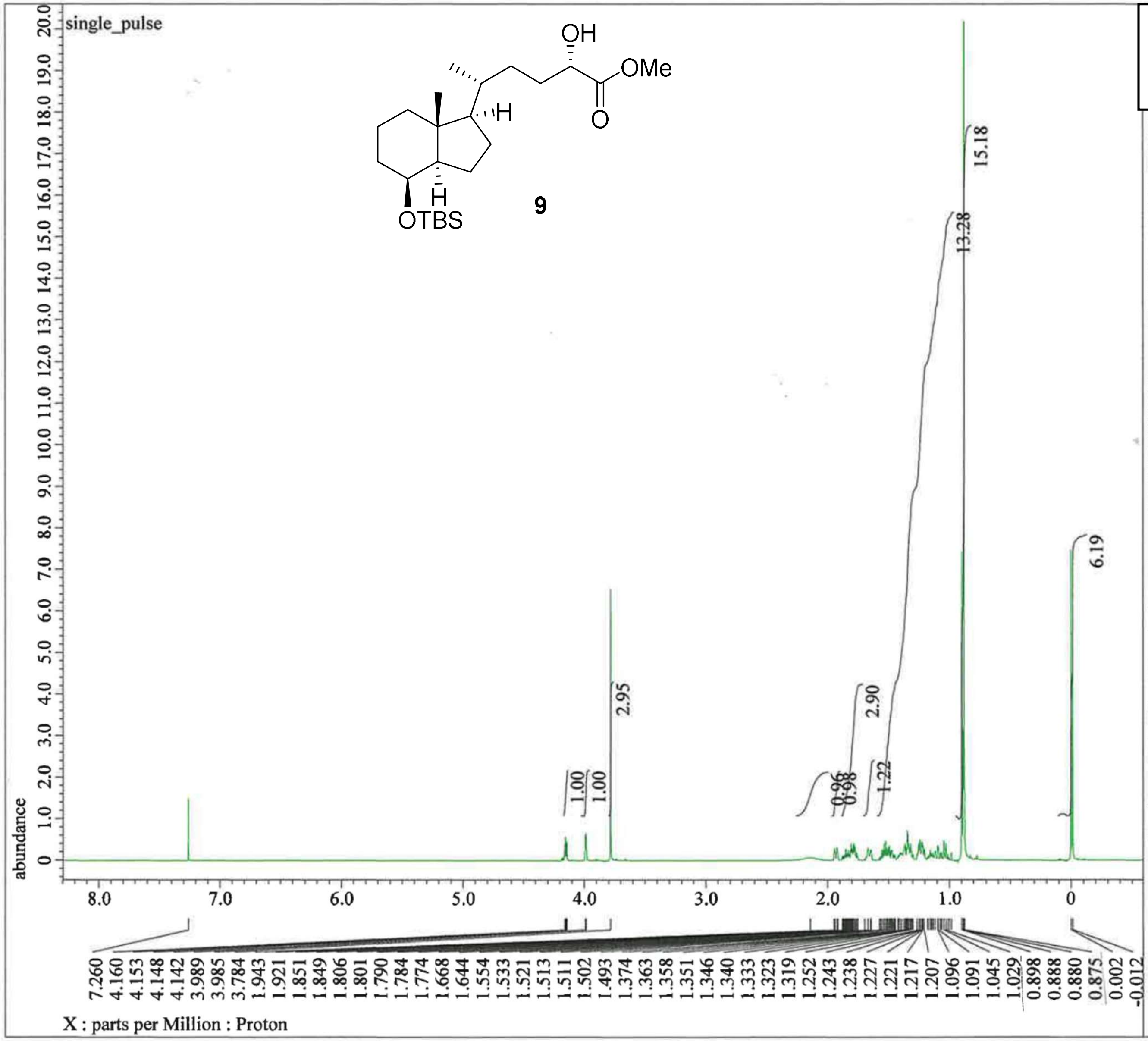


```
Filename          = 41KWG190624-24SOH-COOMe_Pr
Author            = y-yakka
Experiment        = proton.jxp
Sample_Id         = 41KWG190624-24SOH-COOMe
Solvent           = CHLOROFORM-D
Creation_Time     = 24-JUN-2019 17:28:12
Revision_Time     = 24-JUN-2019 17:35:31
Current_Time      = 24-JUN-2019 17:38:43

Comment           = single_pulse
Data_Format       = 1D COMPLEX
Dim_Size          = 13107
Dim_Title         = Proton
Dim_Units         = [ppm]
Dimensions        = X
Site              = JNM-ECA600
Spectrometer      = DELTA2_NMR

Field_Strength    = 14.09636928[T] (600[MHz])
X_Acq_Duration    = 1.4548992[s]
X_Domain          = 1H
X_Freq            = 600.1723046[MHz]
X_Offset          = 5[ppm]
X_Points          = 16384
X_Prescans        = 1
X_Resolution      = 0.68733284[Hz]
X_Sweep           = 11.26126126[kHz]
X_Sweep_Clipped   = 9.00900901[kHz]
Irr_Domain        = Proton
Irr_Freq          = 600.1723046[MHz]
Irr_Offset        = 5[ppm]
Tri_Domain        = Proton
Tri_Freq          = 600.1723046[MHz]
Tri_Offset        = 5[ppm]
Clipped           = FALSE
Scans             = 8
Total_Scans       = 8

Relaxation_Delay  = 5[s]
Recvr_Gain        = 30
Temp_Get          = 23.3[dC]
X_90_Width        = 8.1[us]
X_Acq_Time        = 1.4548992[s]
X_Angle           = 45[deg]
X_Atn             = 2.5[dB]
X_Pulse           = 4.05[us]
Irr_Mode          = Off
Tri_Mode          = Off
Dante_Presat      = FALSE
Initial_Wait      = 1[s]
Repetition_Time   = 6.4548992[s]
```

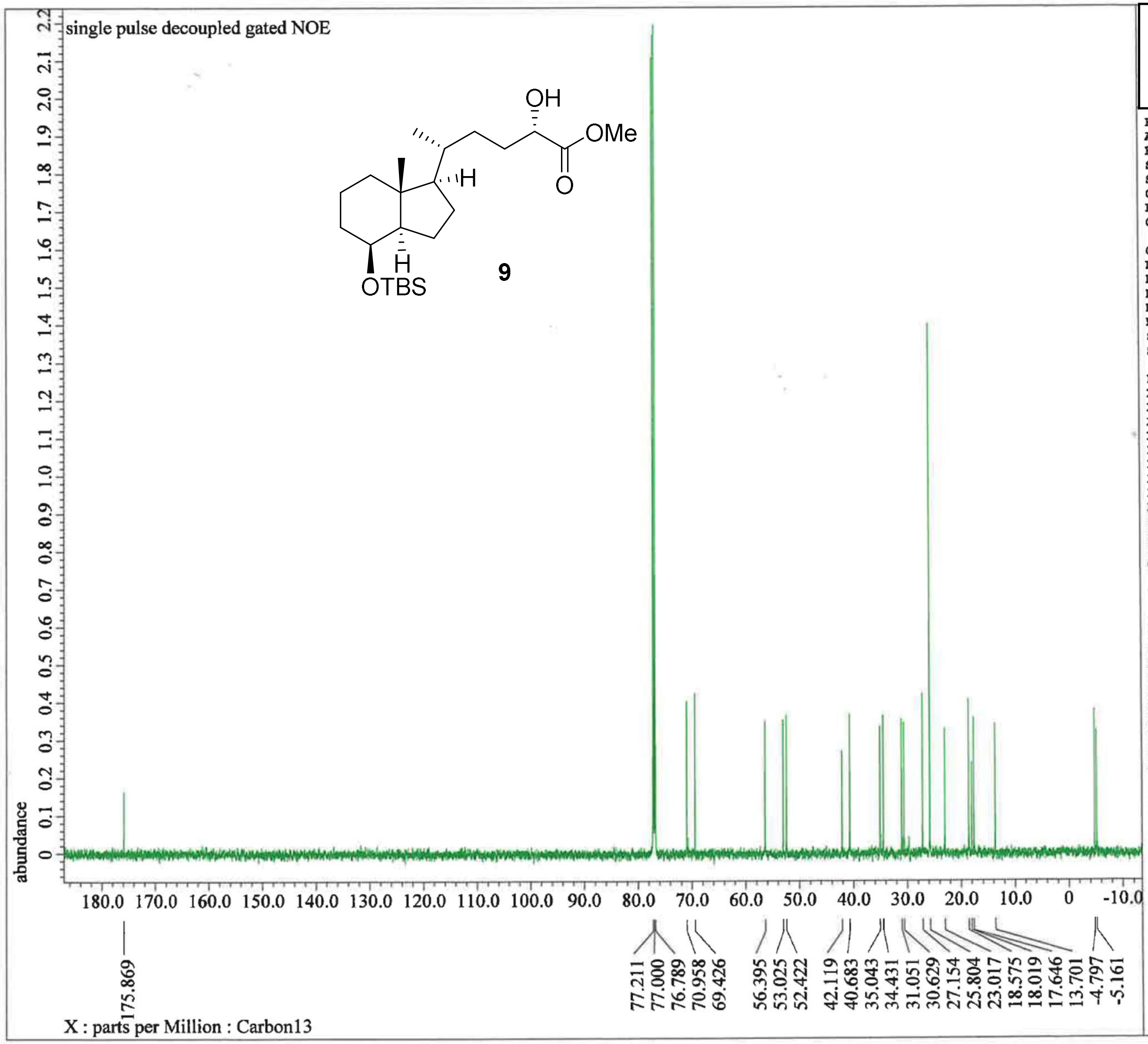

Filename	= 41KWG190624-24SOH-COOMe_Ca
Author	= y-yakka
Experiment	= carbon.jxp
Sample_Id	= 41KWG190624-24SOH-COOMe
Solvent	= CHLOROFORM-D
Creation_Time	= 24-JUN-2019 17:31:24
Revision_Time	= 24-JUN-2019 17:44:49
Current_Time	= 24-JUN-2019 17:45:11
Comment	= single pulse decoupled gat
Data_Format	= 1D COMPLEX
Dim_Size	= 26214
Dim_Title	= Carbon13
Dim_Units	= [ppm]
Dimensions	= X
Site	= JNM-ECA600
Spectrometer	= DELTA2_NMR
Field_Strength	= 14.09636928[T] (600[MHz])
X_Acq_Duration	= 0.69206016[s]
X_Domain	= 13C
X_Freq	= 150.91343039[MHz]
X_Offset	= 100[ppm]
X_Points	= 32768
X_Prescans	= 4
X_Resolution	= 1.44496109[Hz]
X_Sweep	= 47.34848485[kHz]
X_Sweep_Clipped	= 37.87878788[kHz]
Irr_Domain	= Proton
Irr_Freq	= 600.1723046[MHz]
Irr_Offset	= 5[ppm]
Clipped	= FALSE
Scans	= 256
Total_Scans	= 256
Relaxation_Delay	= 2[s]
Recvr_Gain	= 60
Temp_Get	= 23.3[dC]
X_90_Width	= 11.5[us]
X_Acq_Time	= 0.69206016[s]
X_Angle	= 30[deg]
X_Atn	= 12[dB]
X_Pulse	= 3.83333333[us]
Irr_Atn_Dec	= 21.5[dB]
Irr_Atn_Noe	= 21.5[dB]
Irr_Noise	= WALTZ
Irr_Pwidth	= 76[us]
Decoupling	= TRUE
Initial_Wait	= 1[s]
Noe	= TRUE
Noe_Time	= 2[s]
Repetition_Time	= 2.69206016[s]

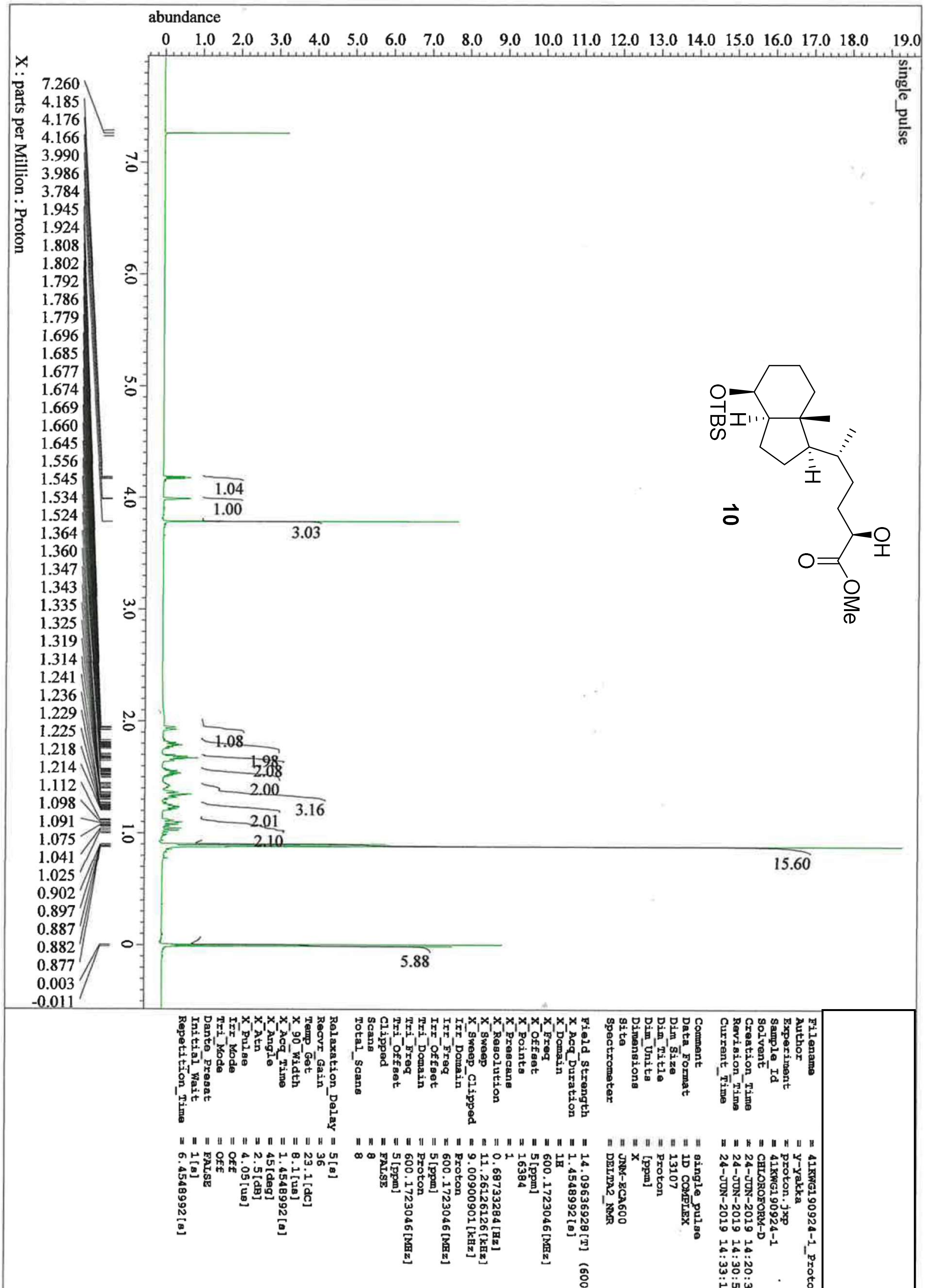
abundance
0 1.0 2.0 3.0 4.0 5.0 6.0 7.0 8.0 9.0 10.0 11.0 12.0 13.0 14.0 15.0 16.0 17.0 18.0 19.0
single_pulse
X : parts per Million : Proton
7.260
4.185
4.176
4.166
3.990
3.986
3.784
1.945
1.924
1.808
1.802
1.792
1.786
1.779
1.696
1.685
1.677
1.674
1.669
1.660
1.645
1.556
1.545
1.534
1.524
1.364
1.360
1.347
1.343
1.335
1.325
1.319
1.314
1.241
1.236
1.229
1.225
1.218
1.214
1.112
1.098
1.091
1.075
1.041
1.025
0.902
0.897
0.887
0.882
0.877
0.003
-0.011
1.04
1.00
3.03
1.08
1.98
2.08
2.00
3.16
2.01
2.10
15.60
5.88
OTBS
H
H
OH
O
OMe
10
Filename = 41KWG190924-1_Proton-1-7.j
Author = Y-yakka
Experiment = proton.jxp
Sample_Id = 41KWG190924-1
Solvent = CHLOROFORM-D
Creation_Time = 24-JUN-2019 14:20:38
Revision_Time = 24-JUN-2019 14:30:53
Current_Time = 24-JUN-2019 14:33:16
Comment = single_pulse
Data_Format = 1D COMPLEX
Dim_Size = 13107
Dim_Title = Proton
Dim_Units = [ppm]
Dimensions = X
Site = JNM-ECA600
Spectrometer = DELTA2_NMR
Field_Strength = 14.09636928[T] (600[MHz])
X_Acq_Duration = 1.4548992[s]
X_Domain = 1H
X_Freq = 600.17230462[MHz]
X_Offset = 5[ppm]
X_Points = 16384
X_Prescans = 1
X_Resolution = 0.68733284[Hz]
X_Sweep = 11.26126126[kHz]
X_Sweep_Clipped = 9.00900901[kHz]
Irr_Domain = Proton
Irr_Freq = 600.17230462[MHz]
Irr_Offset = 5[ppm]
Tri_Domain = Proton
Tri_Freq = 600.17230462[MHz]
Tri_Offset = 5[ppm]
Clipped = FALSE
Scans = 8
Total_Scans = 8
Relaxation_Delay = 5[s]
Recvr_Gain = 36
Temp_Get = 23.1[dC]
X_90_Width = 8.1[us]
X_Acq_Time = 1.4548992[s]
X_Angle = 45[deg]
X_Atn = 2.5[dB]
X_Pulse = 4.05[us]
Irr_Mode = Off
Tri_Mode = Off
Dante_Presat = FALSE
Initial_Wait = 1[s]
Repetition_Time = 6.4548992[s]

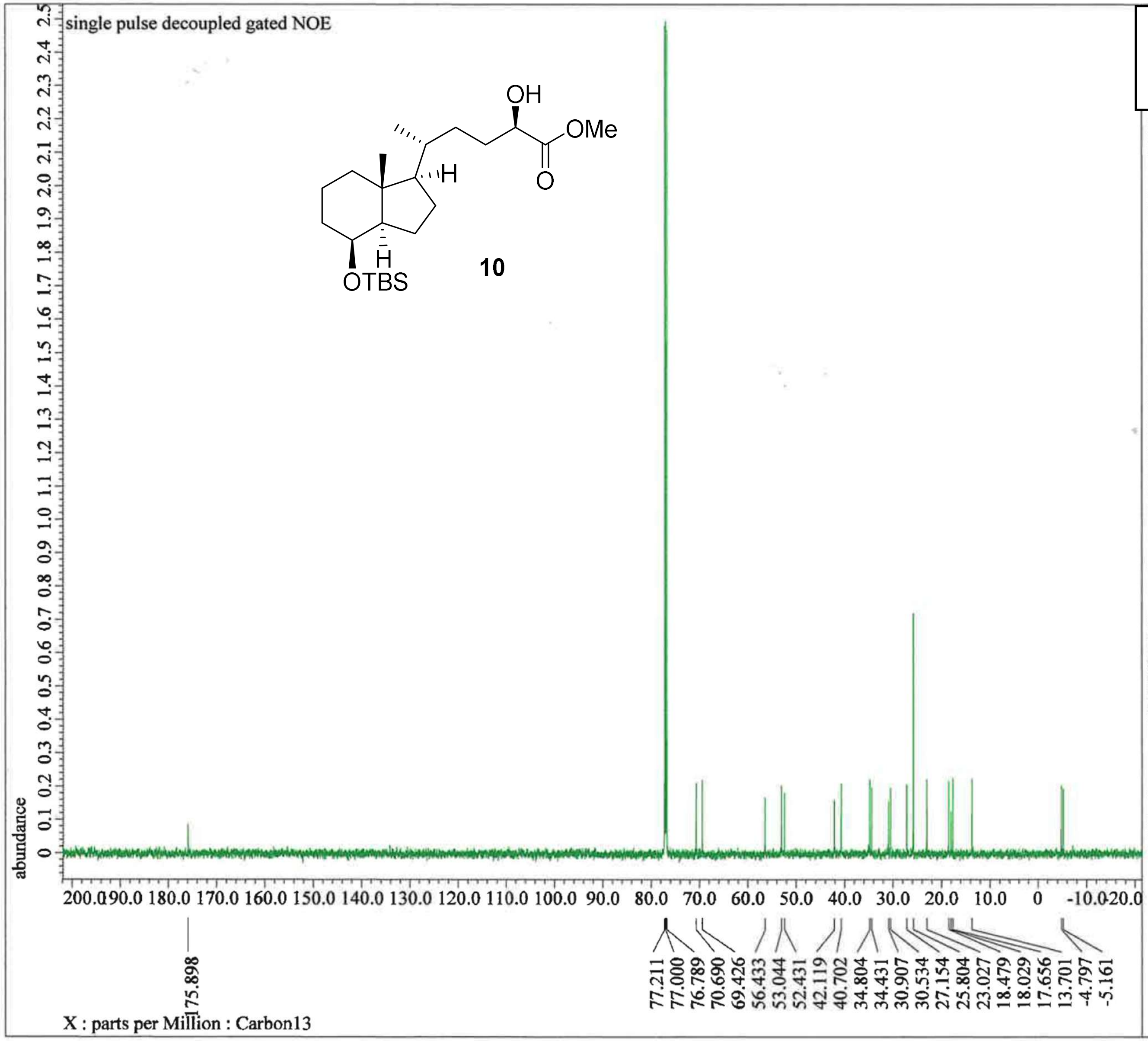

Filename	= 41KWG190924-1_Carbon-1-3.j
Author	= y-yakka
Experiment	= carbon.jxp
Sample_Id	= 41KWG190924-1
Solvent	= CHLOROFORM-D
Creation_Time	= 24-JUN-2019 14:23:52
Revision_Time	= 24-JUN-2019 14:37:27
Current_Time	= 24-JUN-2019 14:38:18
Comment	= single pulse decoupled gat
Data_Format	= 1D COMPLEX
Dim_Size	= 26214
Dim_Title	= Carbon13
Dim_Units	= [ppm]
Dimensions	= X
Site	= JNM-ECA600
Spectrometer	= DELTA2_NMR
Field_Strength	= 14.09636928[T] (600[MHz])
X_Acq_Duration	= 0.69206016[s]
X_Domain	= 13C
X_Freq	= 150.91343039[MHz]
X_Offset	= 100[ppm]
X_Points	= 32768
X_Prescans	= 4
X_Resolution	= 1.44496109[Hz]
X_Sweep	= 47.34848485[kHz]
X_Sweep_Clipped	= 37.87878788[kHz]
Irr_Domain	= Proton
Irr_Freq	= 600.1723046[MHz]
Irr_Offset	= 5[ppm]
Clipped	= FALSE
Scans	= 256
Total_Scans	= 256
Relaxation_Delay	= 2[s]
Recvr_Gain	= 60
Temp_Get	= 23.3[dC]
X_90_Width	= 11.5[us]
X_Acq_Time	= 0.69206016[s]
X_Angle	= 30[deg]
X_Atn	= 12[dB]
X_Pulse	= 3.83333333[us]
Irr_Atn_Dec	= 21.5[dB]
Irr_Atn_Noe	= 21.5[dB]
Irr_Noise	= WALTZ
Irr_Pwidth	= 76[us]
Decoupling	= TRUE
Initial_Wait	= 1[s]
Noe	= TRUE
Noe_Time	= 2[s]
Repetition_Time	= 2.69206016[s]

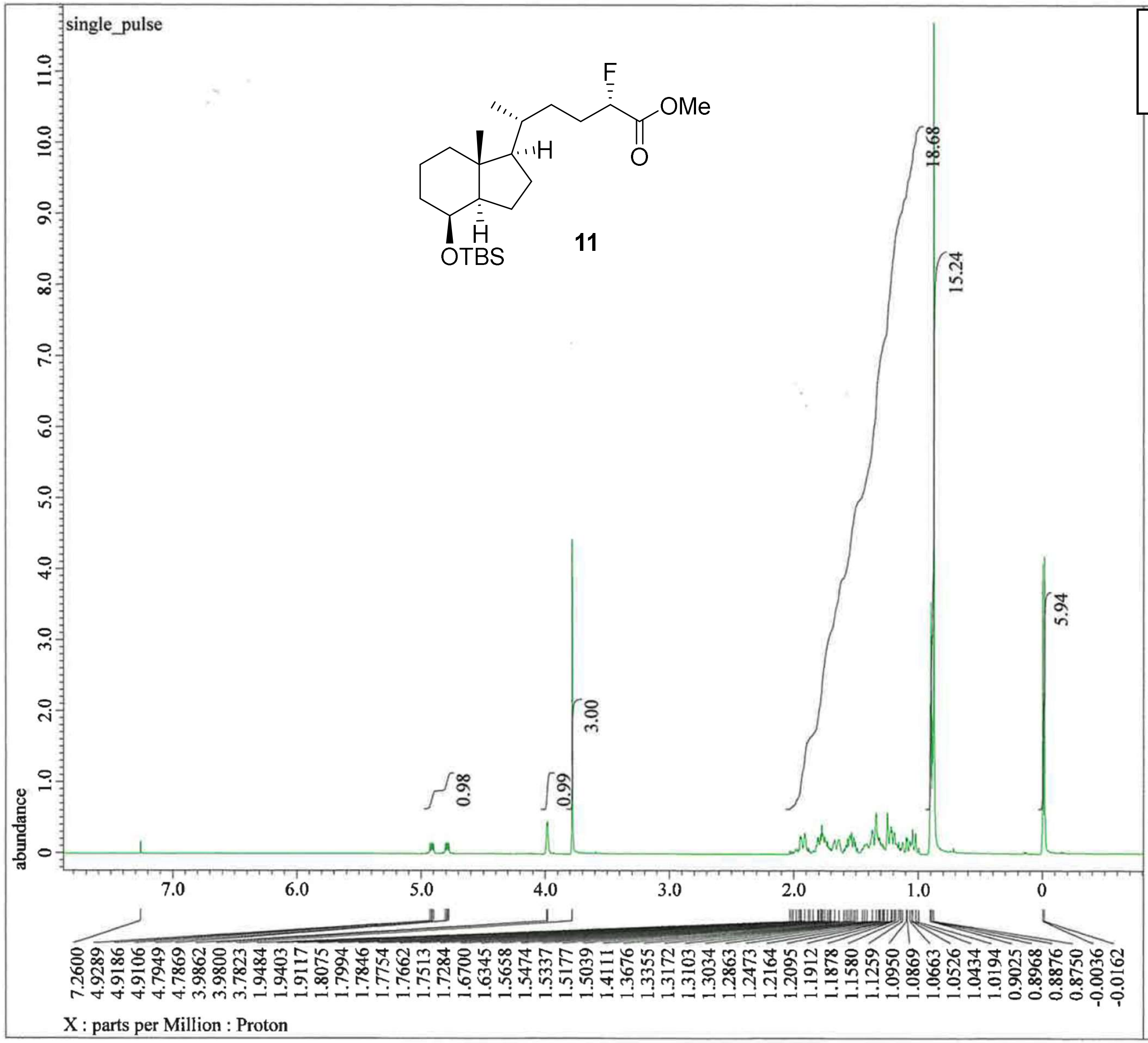

Filename	= 41KWG190625-1_Proton_copy1
Author	= y-yakka
Experiment	= proton.jxp
Sample_Id	= 41KWG190625-1
Solvent	= CHLOROFORM-D
Creation_Time	= 25-JUN-2019 16:38:23
Revision_Time	= 25-JUN-2019 16:42:10
Current_Time	= 25-JUN-2019 16:42:23
Comment	= single_pulse
Data_Format	= 1D COMPLEX
Dim_Size	= 13107
Dim_Title	= Proton
Dim_Units	= [ppm]
Dimensions	= X
Site	= JNM-ECS400
Spectrometer	= DELTA2_NMR
Field_Strength	= 9.389766[T] (400[MHz])
X_Acq_Duration	= 0[s]
X_Domain	= 1H
X_Freq	= 399.78219838[MHz]
X_Offset	= 5[ppm]
X_Points	= 16384
X_Prescans	= 1
X_Resolution	= 0.45794685[Hz]
X_Sweep	= 7.5030012[kHz]
X_Sweep_Clipped	= 6.00240096[kHz]
Irr_Domain	= Proton
Irr_Freq	= 399.78219838[MHz]
Irr_Offset	= 5[ppm]
Tri_Domain	= Proton
Tri_Freq	= 399.78219838[MHz]
Tri_Offset	= 5[ppm]
Clipped	= FALSE
Incomplete_Copy	= TRUE
Scans	= 6
Total_Scans	= 6
Relaxation_Delay	= 5[s]
Recvr_Gain	= 18
Temp_Get	= 24.5[dC]
X_90_Width	= 6.5[us]
X_Acq_Time	= 2.18365952[s]
X_Angle	= 45[deg]
X_Atn	= 1.1[dB]
X_Pulse	= 3.25[us]
Irr_Mode	= Off
Tri_Mode	= Off
Dante_Presat	= FALSE
Initial_Wait	= 1[s]
Repetition_Time	= 7.18365952[s]

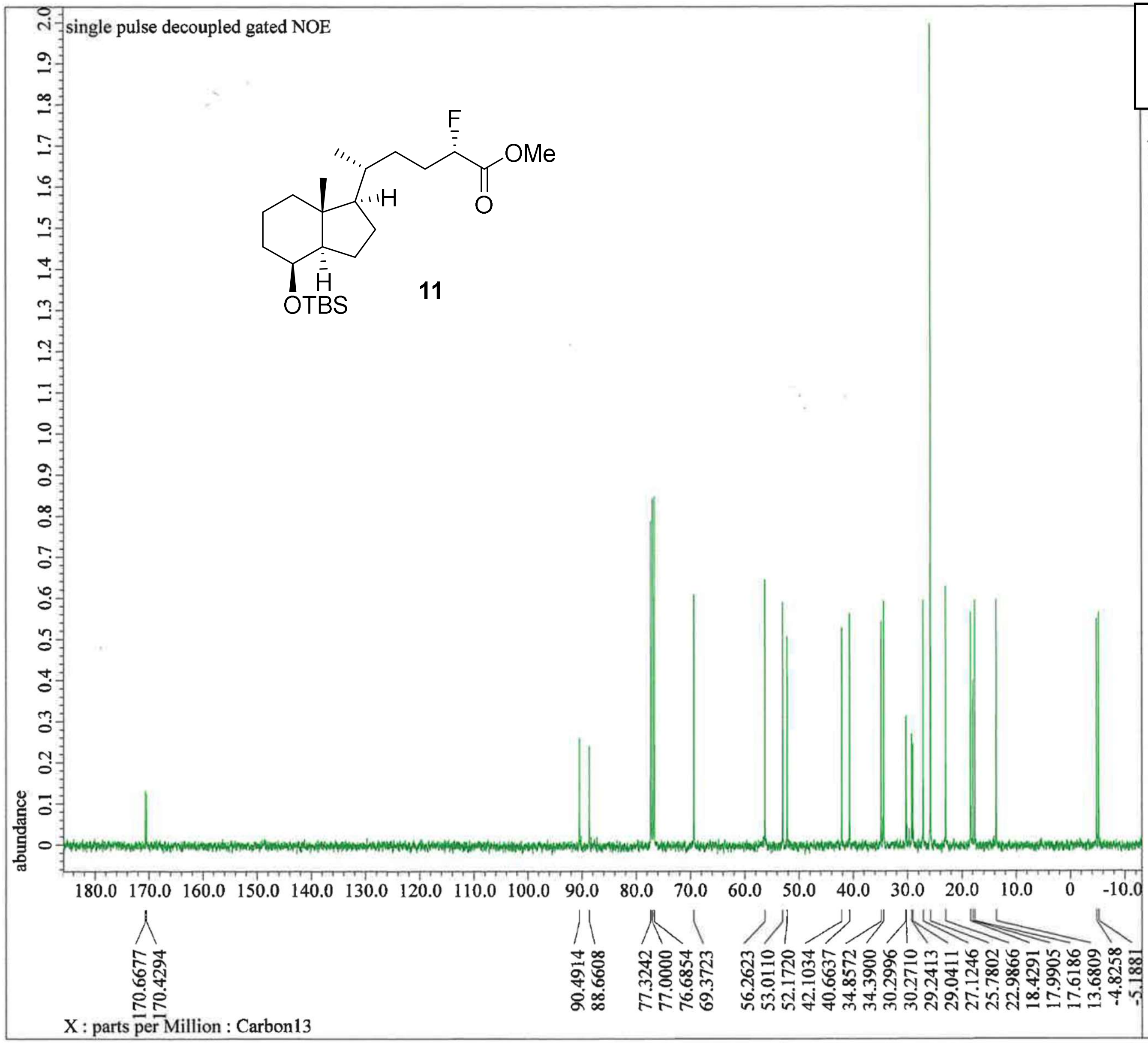


```
Filename          = 41KWG190625-1_Carbon_copy2
Author            = y-yakka
Experiment        = carbon.jxp
Sample_Id         = 41KWG190625-1
Solvent           = CHLOROFORM-D
Creation_Time     = 25-JUN-2019 16:42:20
Revision_Time     = 25-JUN-2019 16:49:40
Current_Time      = 25-JUN-2019 16:49:50

Comment           = single pulse decoupled gat
Data_Format       = 1D COMPLEX
Dim_Size          = 26214
Dim_Title         = Carbon13
Dim_Units         = [ppm]
Dimensions        = X
Site              = JNM-ECS400
Spectrometer      = DELTA2_NMR

Field_Strength    = 9.389766[T] (400[MHz])
X_Acq_Duration    = 0[s]
X_Domain          = 13C
X_Freq            = 100.52530333[MHz]
X_Offset          = 100[ppm]
X_Points          = 32768
X_Prescans        = 4
X_Resolution      = 0.95846665[Hz]
X_Sweep           = 31.40703518[kHz]
X_Sweep_Clipped   = 25.12562814[kHz]
Irr_Domain        = Proton
Irr_Freq          = 399.78219838[MHz]
Irr_Offset        = 5[ppm]
Clipped           = FALSE
Incomplete_Copy   = TRUE
Scans             = 116.0
Total_Scans       = 116.0

Relaxation_Delay  = 2[s]
Recvr_Gain        = 60
Temp_Get          = 24.5[dC]
X_90_Width        = 9.7[us]
X_Acq_Time        = 1.04333312[s]
X_Angle           = 30[deg]
X_Atn             = 3[dB]
X_Pulse           = 3.23333333[us]
Irr_Atn_Dec       = 25.6[dB]
Irr_Atn_Noe       = 25.6[dB]
Irr_Noise         = WALTZ
Irr_Pwidth        = 0.115[ms]
Decoupling        = TRUE
Initial_Wait      = 1[s]
Noe               = TRUE
Noe_Time          = 2[s]
Repetition_Time   = 3.04333312[s]
```

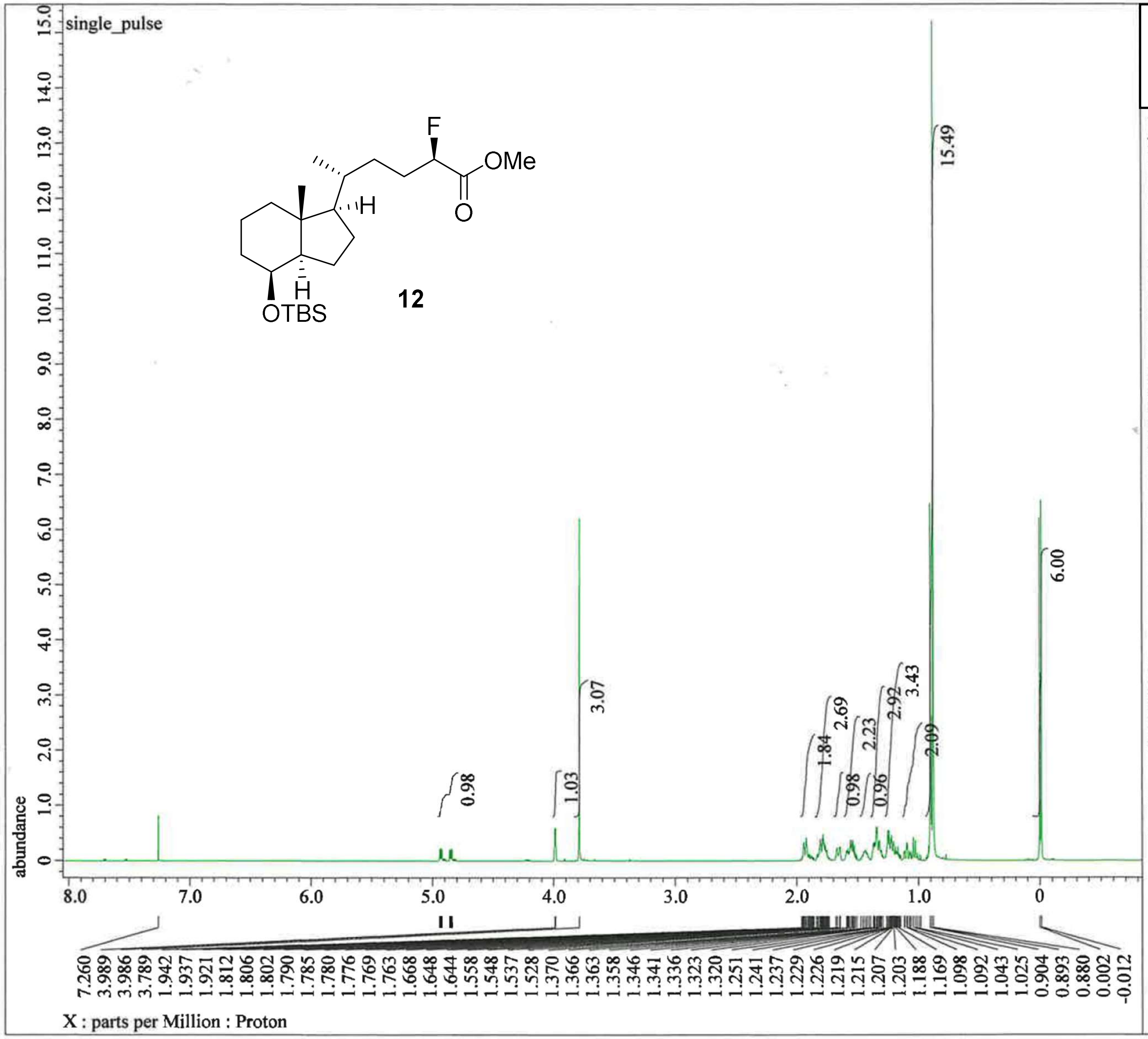

Filename	= 41KWG190627-2_Proton-1-7.j
Author	= y-yakka
Experiment	= proton.jxp
Sample_Id	= 41KWG190627-2
Solvent	= CHLOROFORM-D
Creation_Time	= 27-JUN-2019 15:17:04
Revision_Time	= 27-JUN-2019 15:33:58
Current_Time	= 27-JUN-2019 15:34:06
Comment	= single_pulse
Data_Format	= 1D COMPLEX
Dim_Size	= 13107
Dim_Title	= Proton
Dim_Units	= [ppm]
Dimensions	= X
Site	= JNM-ECA600
Spectrometer	= DELTA2_NMR
Field_Strength	= 14.09636928[T] (600[MHz])
X_Acq_Duration	= 1.4548992[s]
X_Domain	= 1H
X_Freq	= 600.1723046[MHz]
X_Offset	= 5[ppm]
X_Points	= 16384
X_Prescans	= 1
X_Resolution	= 0.68733284[Hz]
X_Sweep	= 11.26126126[kHz]
X_Sweep_Clipped	= 9.00900901[kHz]
Irr_Domain	= Proton
Irr_Freq	= 600.1723046[MHz]
Irr_Offset	= 5[ppm]
Tri_Domain	= Proton
Tri_Freq	= 600.1723046[MHz]
Tri_Offset	= 5[ppm]
Clipped	= FALSE
Scans	= 8
Total_Scans	= 8
Relaxation_Delay	= 5[s]
Recvr_Gain	= 24
Temp_Get	= 22.5[dC]
X_90_Width	= 8.1[us]
X_Acq_Time	= 1.4548992[s]
X_Angle	= 45[deg]
X_Atn	= 2.5[dB]
X_Pulse	= 4.05[us]
Irr_Mode	= Off
Tri_Mode	= Off
Dante_Presat	= FALSE
Initial_Wait	= 1[s]
Repetition_Time	= 6.4548992[s]

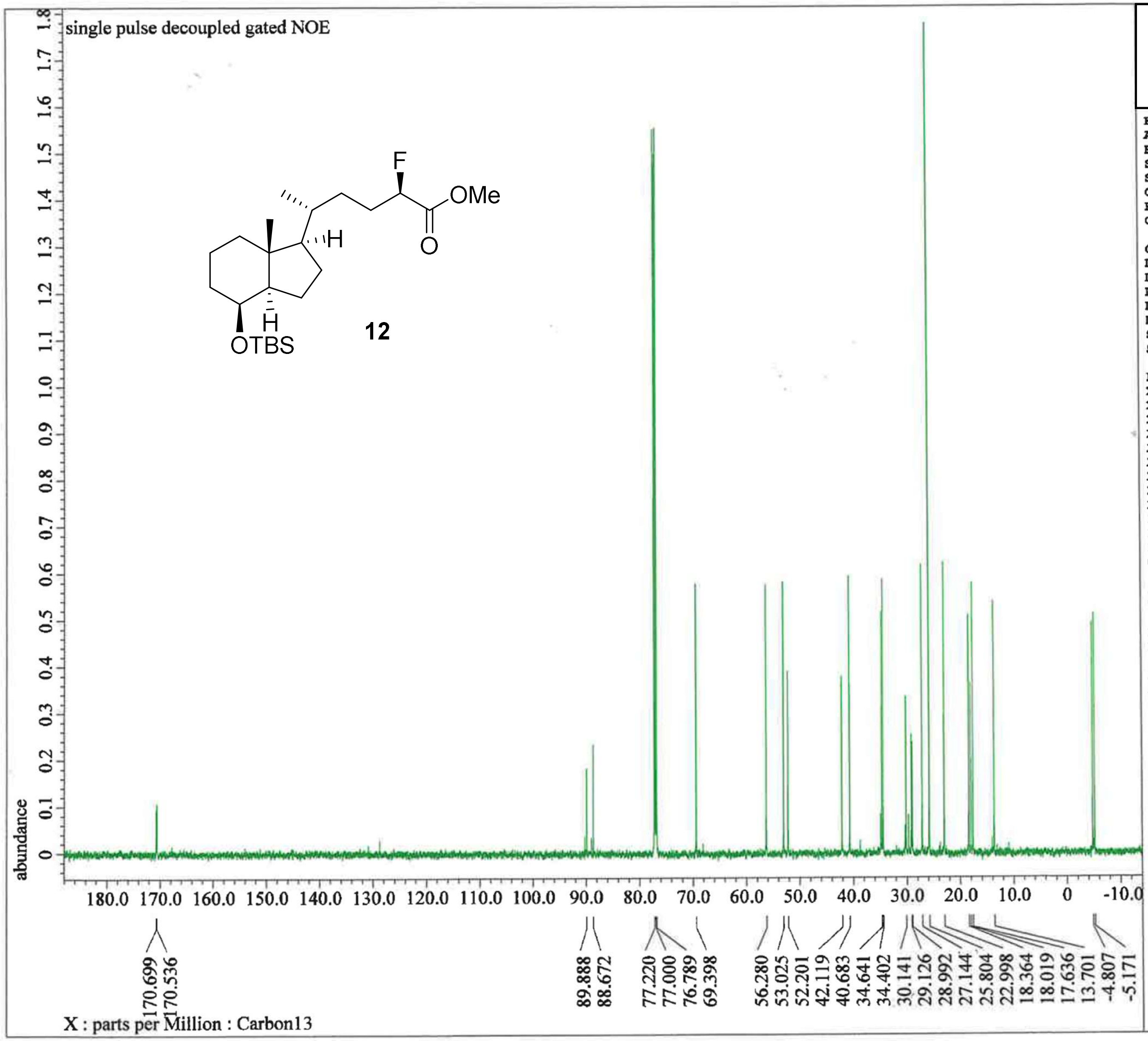

Filename	= 41KWG190627-2_Carbon_copy1
Author	= y-yakka
Experiment	= carbon.jxp
Sample_Id	= 41KWG190627-2
Solvent	= CHLOROFORM-D
Creation_Time	= 27-JUN-2019 15:20:22
Revision_Time	= 27-JUN-2019 15:41:54
Current_Time	= 27-JUN-2019 15:42:24
Comment	= single pulse decoupled gat
Data_Format	= 1D COMPLEX
Dim_Size	= 26214
Dim_Title	= Carbon13
Dim_Units	= [ppm]
Dimensions	= X
Site	= JNM-ECA600
Spectrometer	= DELTA2_NMR
Field_Strength	= 14.09636928[T] (600[MHz])
X_Acq_Duration	= 0[s]
X_Domain	= 13C
X_Freq	= 150.91343039[MHz]
X_Offset	= 100[ppm]
X_Points	= 32768
X_Prescans	= 4
X_Resolution	= 1.44496109[Hz]
X_Sweep	= 47.34848485[kHz]
X_Sweep_Clipped	= 37.87878788[kHz]
Irr_Domain	= Proton
Irr_Freq	= 600.1723046[MHz]
Irr_Offset	= 5[ppm]
Clipped	= FALSE
Incomplete_Copy	= TRUE
Scans	= 419
Total_Scans	= 419
Relaxation_Delay	= 2[s]
Recvr_Gain	= 58
Temp_Get	= 23.2[dC]
X_90_Width	= 11.5[us]
X_Acq_Time	= 0.69206016[s]
X_Angle	= 30[deg]
X_Atn	= 12[dB]
X_Pulse	= 3.83333333[us]
Irr_Atn_Dec	= 21.5[dB]
Irr_Atn_Noe	= 21.5[dB]
Irr_Noise	= WALTZ
Irr_Pwidth	= 76[us]
Decoupling	= TRUE
Initial_Wait	= 1[s]
Noe	= TRUE
Noe_Time	= 2[s]
Repetition_Time	= 2.69206016[s]

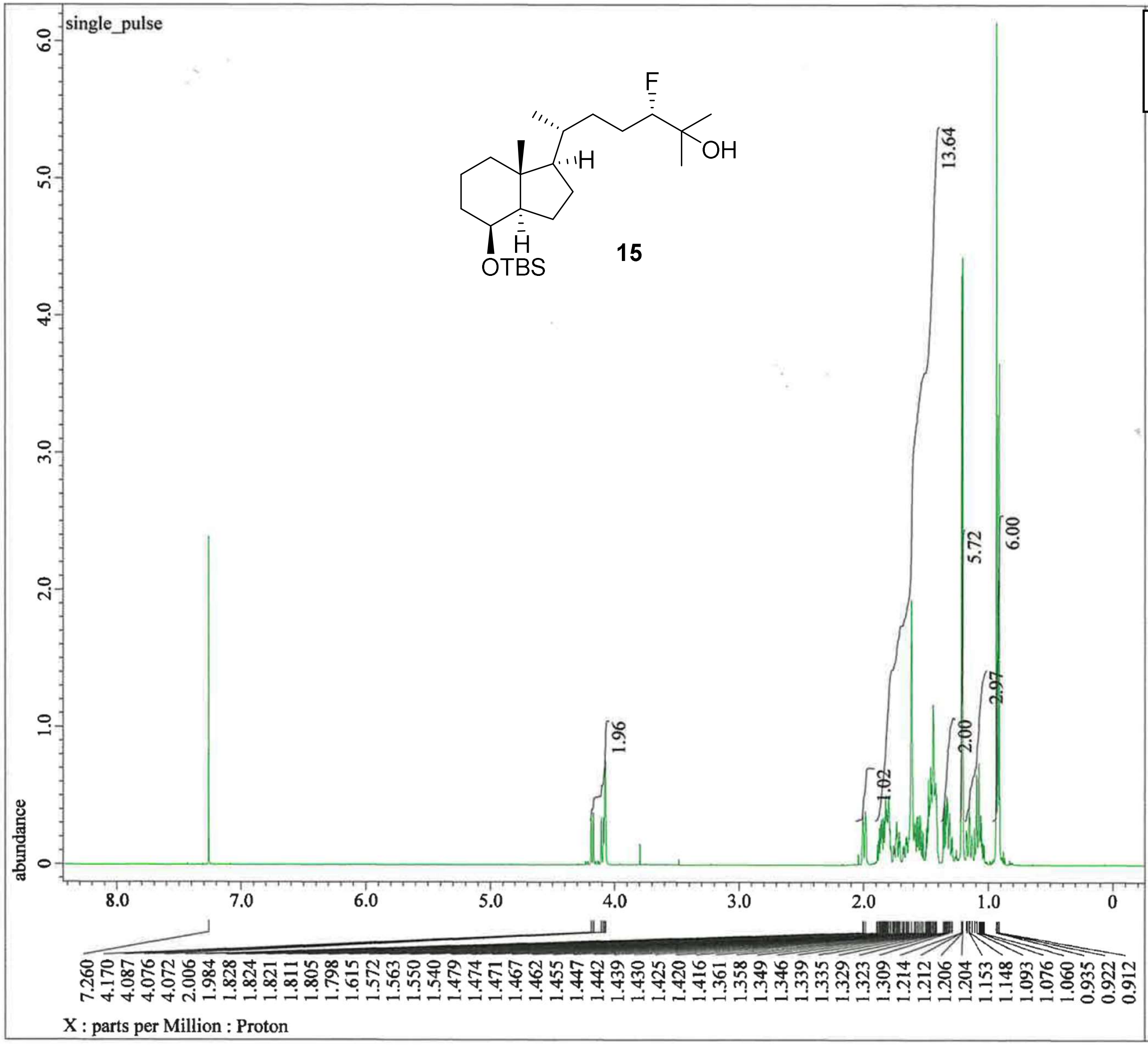


```
Filename          = 41KWG190628-2_Proton-1-4.j
Author            = y-yakka
Experiment        = proton.jxp
Sample_Id         = 41KWG190628-2
Solvent           = CHLOROFORM-D
Creation_Time     = 28-JUN-2019 14:06:00
Revision_Time     = 28-JUN-2019 14:31:59
Current_Time      = 28-JUN-2019 14:32:04

Comment           = single_pulse
Data_Format       = 1D COMPLEX
Dim_Size          = 13107
Dim_Title         = Proton
Dim_Units         = [ppm]
Dimensions        = X
Site              = JNM-ECA600
Spectrometer      = DELTA2_NMR

Field_Strength    = 14.09636928[T] (600[MHz])
X_Acq_Duration    = 1.4548992[s]
X_Domain          = 1H
X_Freq            = 600.1723046[MHz]
X_Offset          = 5[ppm]
X_Points          = 16384
X_Prescans        = 1
X_Resolution      = 0.68733284[Hz]
X_Sweep           = 11.26126126[kHz]
X_Sweep_Clipped   = 9.00900901[kHz]
Irr_Domain        = Proton
Irr_Freq          = 600.1723046[MHz]
Irr_Offset        = 5[ppm]
Tri_Domain        = Proton
Tri_Freq          = 600.1723046[MHz]
Tri_Offset        = 5[ppm]
Clipped           = FALSE
Scans             = 8
Total_Scans       = 8

Relaxation_Delay  = 5[s]
Recvr_Gain        = 34
Temp_Get          = 23.4[dC]
X_90_Width        = 8.1[us]
X_Acq_Time        = 1.4548992[s]
X_Angle           = 45[deg]
X_Atn             = 2.5[dB]
X_Pulse           = 4.05[us]
Irr_Mode          = Off
Tri_Mode          = Off
Dante_Presat      = FALSE
Initial_Wait      = 1[s]
Repetition_Time   = 6.4548992[s]
```

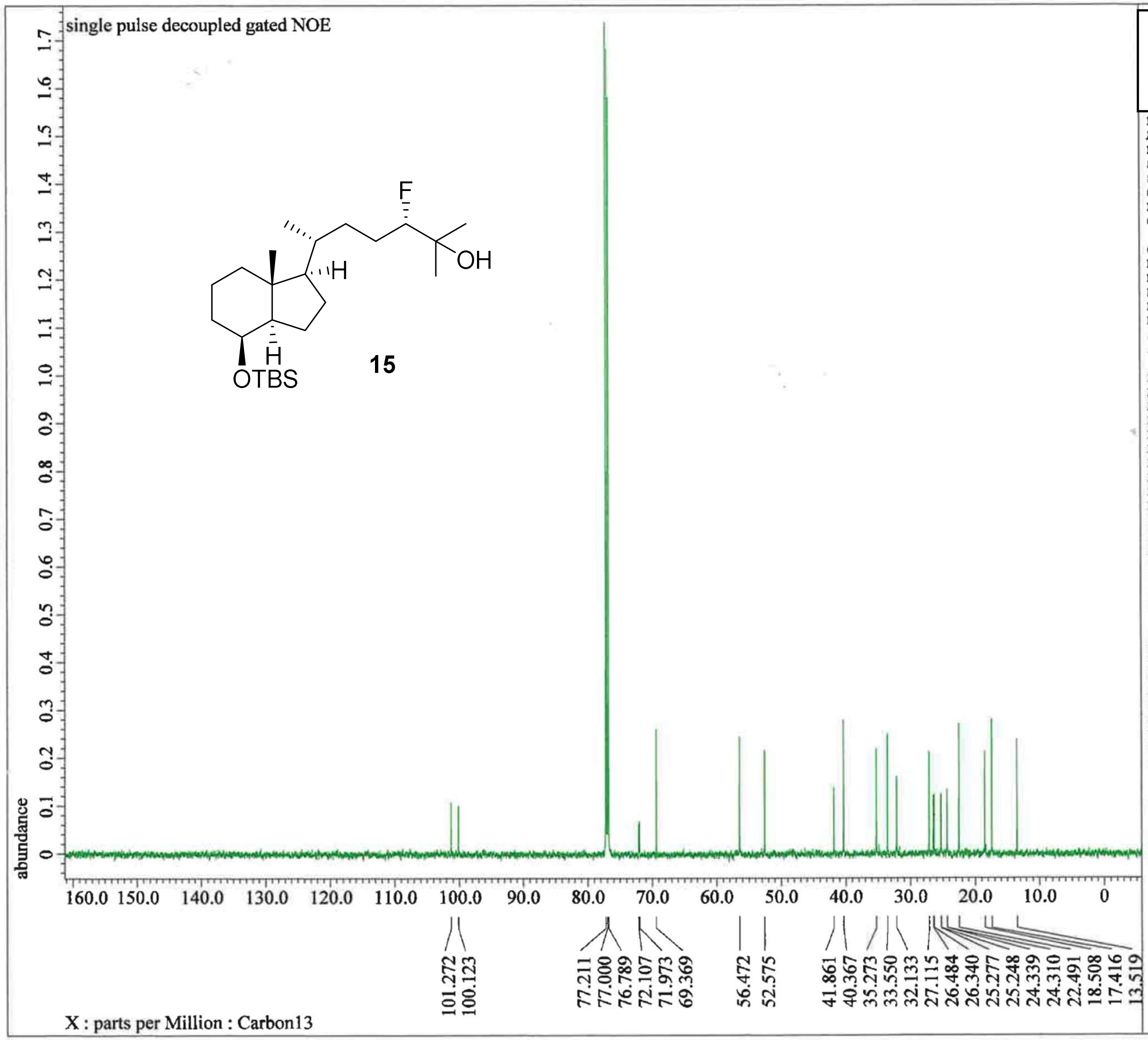

Filename	= 41KWG190628-4_Carbon_copy1
Author	= y-yakka
Experiment	= carbon.jxp
Sample_Id	= 41KWG190628-4
Solvent	= CHLOROFORM-D
Creation_Time	= 28-JUN-2019 14:34:21
Revision_Time	= 28-JUN-2019 14:57:38
Current_Time	= 28-JUN-2019 14:57:48
Comment	= single pulse decoupled gat
Data_Format	= 1D COMPLEX
Dim_Size	= 26214
Dim_Title	= Carbon13
Dim_Units	= [ppm]
Dimensions	= X
Site	= JNM-ECA600
Spectrometer	= DELTA2_NMR
Field_Strength	= 14.09636928[T] (600[MHz])
X_Acq_Duration	= 0[s]
X_Domain	= 13C
X_Freq	= 150.91343039[MHz]
X_Offset	= 100[ppm]
X_Points	= 32768
X_Prescans	= 4
X_Resolution	= 1.44496109[Hz]
X_Sweep	= 47.34848485[kHz]
X_Sweep_Clipped	= 37.87878788[kHz]
Irr_Domain	= Proton
Irr_Freq	= 600.1723046[MHz]
Irr_Offset	= 5[ppm]
Clipped	= FALSE
Incomplete_Copy	= TRUE
Scans	= 497
Total_Scans	= 497
Relaxation_Delay	= 2[s]
Recvr_Gain	= 58
Temp_Get	= 23.2[dC]
X_90_Width	= 11.5[us]
X_Acq_Time	= 0.69206016[s]
X_Angle	= 30[deg]
X_Atn	= 12[dB]
X_Pulse	= 3.83333333[us]
Irr_Atn_Dec	= 21.5[dB]
Irr_Atn_Noe	= 21.5[dB]
Irr_Noise	= WALTZ
Irr_Pwidth	= 76[us]
Decoupling	= TRUE
Initial_Wait	= 1[s]
Noe	= TRUE
Noe_Time	= 2[s]
Repetition_Time	= 2.69206016[s]

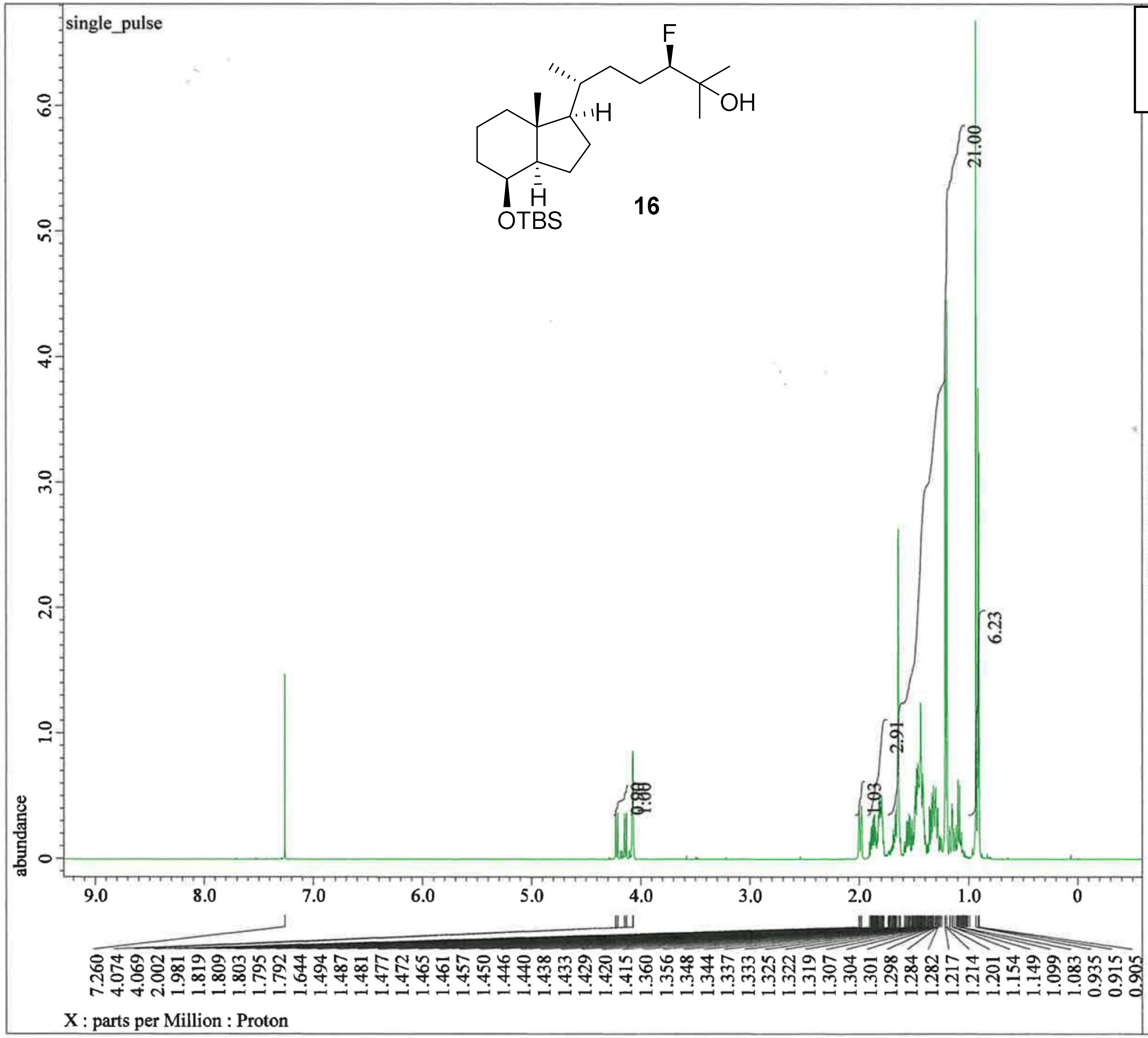

Filename = 41KWG190703-1_Proton-1-5.j
Author = y-yakka
Experiment = proton.jxp
Sample_Id = 41KWG190703-1
Solvent = CHLOROFORM-D
Creation_Time = 3-JUL-2019 10:29:50
Revision_Time = 3-JUL-2019 10:37:38
Current_Time = 3-JUL-2019 10:37:43

Comment = single_pulse
Data_Format = 1D COMPLEX
Dim_Size = 13107
Dim_Title = Proton
Dim_Units = [ppm]
Dimensions = X
Site = JNM-ECA600
Spectrometer = DELTA2_NMR

Field_Strength = 14.09636928[T] (600[MHz])
X_Acq_Duration = 1.4548992[s]
X_Domain = 1H
X_Freq = 600.1723046[MHz]
X_Offset = 5[ppm]
X_Points = 16384
X_Prescans = 1
X_Resolution = 0.68733284[Hz]
X_Sweep = 11.26126126[kHz]
X_Sweep_Clipped = 9.00900901[kHz]
Irr_Domain = Proton
Irr_Freq = 600.1723046[MHz]
Irr_Offset = 5[ppm]
Tri_Domain = Proton
Tri_Freq = 600.1723046[MHz]
Tri_Offset = 5[ppm]
Clipped = FALSE
Scans = 8
Total_Scans = 8

Relaxation_Delay = 5[s]
Recvr_Gain = 30
Temp_Get = 23.1[dC]
X_90_Width = 8.1[us]
X_Acq_Time = 1.4548992[s]
X_Angle = 45[deg]
X_Atn = 2.5[dB]
X_Pulse = 4.05[us]
Irr_Mode = Off
Tri_Mode = Off
Dante_Presat = FALSE
Initial_Wait = 1[s]
Repetition_Time = 6.4548992[s]

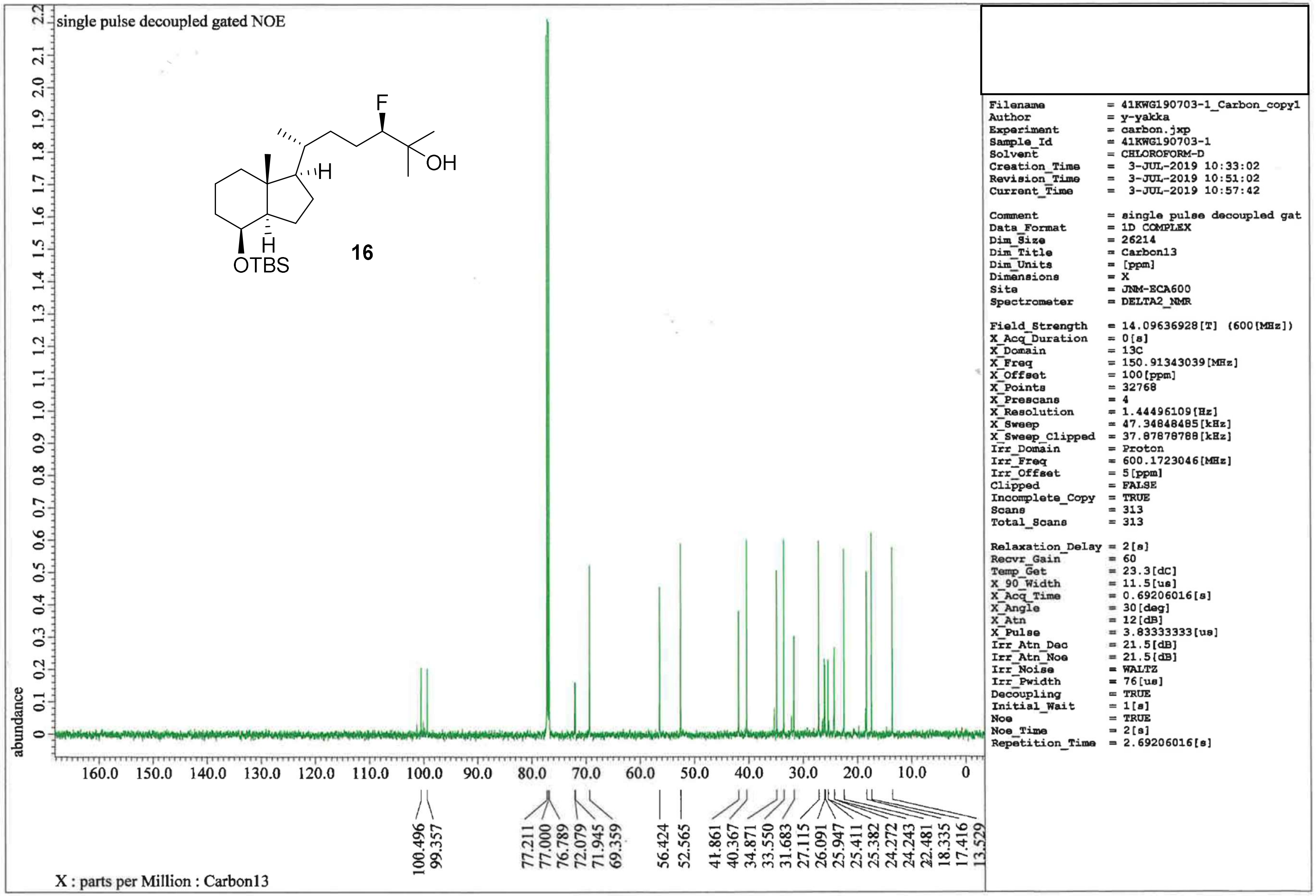
single pulse decoupled gated NOE
F
OH
H
H
OTBS
16
abundance
0 0.1 0.2 0.3 0.4 0.5 0.6 0.7 0.8 0.9 1.0 1.1 1.2 1.3 1.4 1.5 1.6 1.7 1.8 1.9 2.0 2.1 2.2
160.0 150.0 140.0 130.0 120.0 110.0 100.0 90.0 80.0 70.0 60.0 50.0 40.0 30.0 20.0 10.0 0
100.496
99.357
77.211
77.000
76.789
72.079
71.945
69.359
56.424
52.565
41.861
40.367
34.871
33.550
31.683
27.115
26.091
25.947
25.411
25.382
24.272
24.243
22.481
18.335
17.416
13.529
X : parts per Million : Carbon13
Filename = 41KWG190703-1_Carbon_copy1
Author = y-yakka
Experiment = carbon.jxp
Sample_Id = 41KWG190703-1
Solvent = CHLOROFORM-D
Creation_Time = 3-JUL-2019 10:33:02
Revision_Time = 3-JUL-2019 10:51:02
Current_Time = 3-JUL-2019 10:57:42
Comment = single pulse decoupled gat
Data_Format = 1D COMPLEX
Dim_Size = 26214
Dim_Title = Carbon13
Dim_Units = [ppm]
Dimensions = X
Site = JNM-ECA600
Spectrometer = DELTA2_NMR
Field_Strength = 14.09636928[T] (600[MHz])
X_Acq_Duration = 0[s]
X_Domain = 13C
X_Freq = 150.91343039[MHz]
X_Offset = 100[ppm]
X_Points = 32768
X_Prescans = 4
X_Resolution = 1.44496109[Hz]
X_Sweep = 47.34848485[kHz]
X_Sweep_Clipped = 37.87878788[kHz]
Irr_Domain = Proton
Irr_Freq = 600.1723046[MHz]
Irr_Offset = 5[ppm]
Clipped = FALSE
Incomplete_Copy = TRUE
Scans = 313
Total_Scans = 313
Relaxation_Delay = 2[s]
Recvr_Gain = 60
Temp_Get = 23.3[dC]
X_90_Width = 11.5[us]
X_Acq_Time = 0.69206016[s]
X_Angle = 30[deg]
X_Atn = 12[dB]
X_Pulse = 3.83333333[us]
Irr_Atn_Dec = 21.5[dB]
Irr_Atn_Noe = 21.5[dB]
Irr_Noise = WALTZ
Irr_Pwidth = 76[us]
Decoupling = TRUE
Initial_Wait = 1[s]
Noe = TRUE
Noe_Time = 2[s]
Repetition_Time = 2.69206016[s]

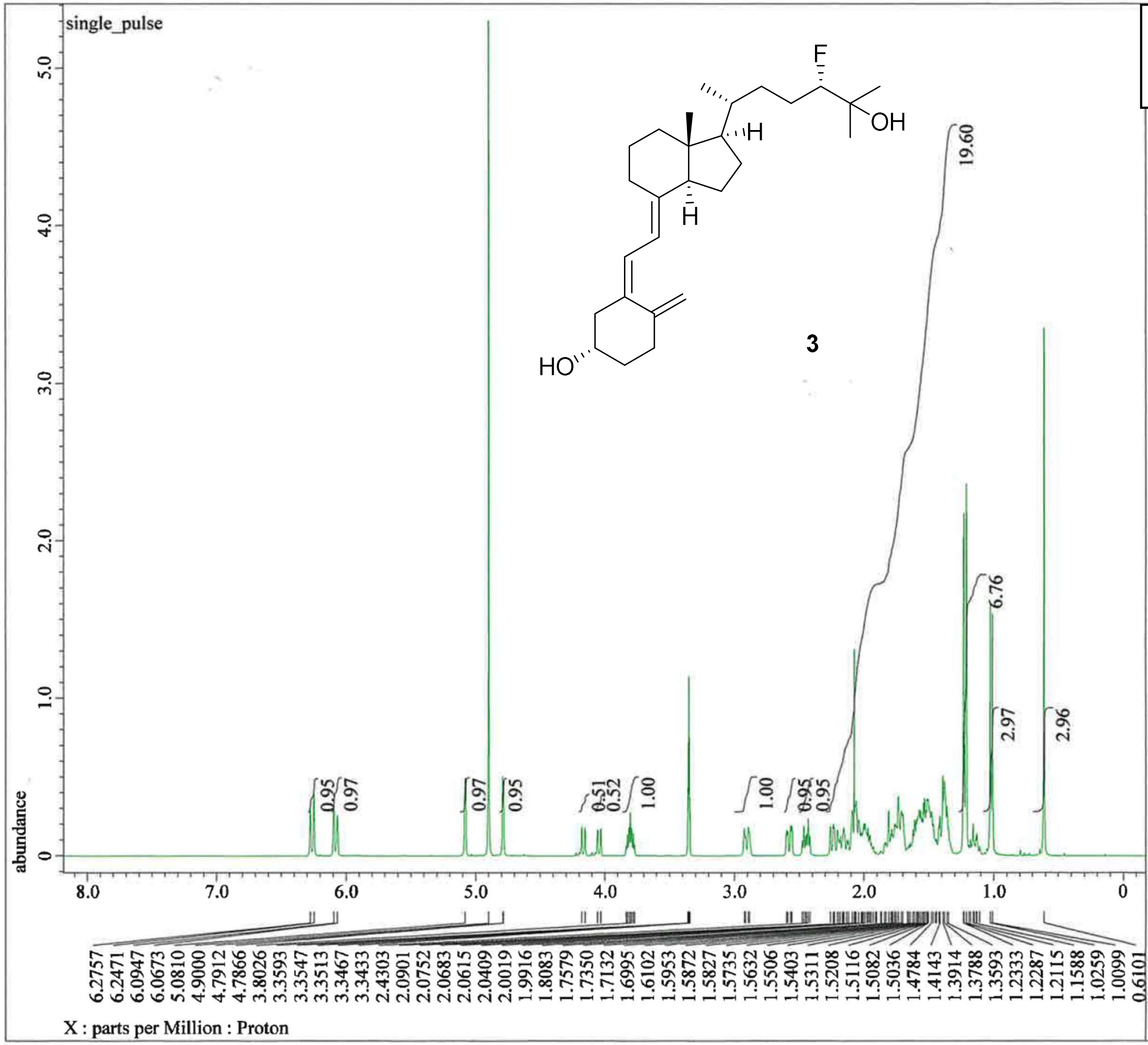


```
Filename          = 41KWG200116-2_Proton-1-4.j
Author            = y-yakka
Experiment        = proton.jxp
Sample_Id         = 41KWG200116-2
Solvent           = METHANOL-D4
Creation_Time     = 16-JAN-2020 13:01:33
Revision_Time     = 16-JAN-2020 13:55:58
Current_Time      = 16-JAN-2020 13:56:12

Comment           = single_pulse
Data_Format       = 1D COMPLEX
Dim_Size          = 13107
Dim_Title         = Proton
Dim_Units         = [ppm]
Dimensions        = X
Site              = JNM-ECS400
Spectrometer      = DELTA2_NMR

Field_Strength    = 9.389766[T] (400[MHz])
X_Acq_Duration    = 2.18365952[s]
X_Domain          = 1H
X_Freq            = 399.78219838[MHz]
X_Offset          = 5[ppm]
X_Points          = 16384
X_Prescans        = 1
X_Resolution      = 0.45794685[Hz]
X_Sweep           = 7.5030012[kHz]
X_Sweep_Clipped   = 6.00240096[kHz]
Irr_Domain        = Proton
Irr_Freq          = 399.78219838[MHz]
Irr_Offset        = 5[ppm]
Tri_Domain        = Proton
Tri_Freq          = 399.78219838[MHz]
Tri_Offset        = 5[ppm]
Clipped           = FALSE
Scans             = 8
Total_Scans       = 8

Relaxation_Delay  = 5[s]
Recvr_Gain        = 30
Temp_Get          = 23.8[dC]
X_90_Width        = 6.5[us]
X_Acq_Time        = 2.18365952[s]
X_Angle           = 45[deg]
X_Atn             = 1.1[dB]
X_Pulse           = 3.25[us]
Irr_Mode          = Off
Tri_Mode          = Off
Dante_Presat      = FALSE
Initial_Wait      = 1[s]
Repetition_Time   = 7.18365952[s]
```

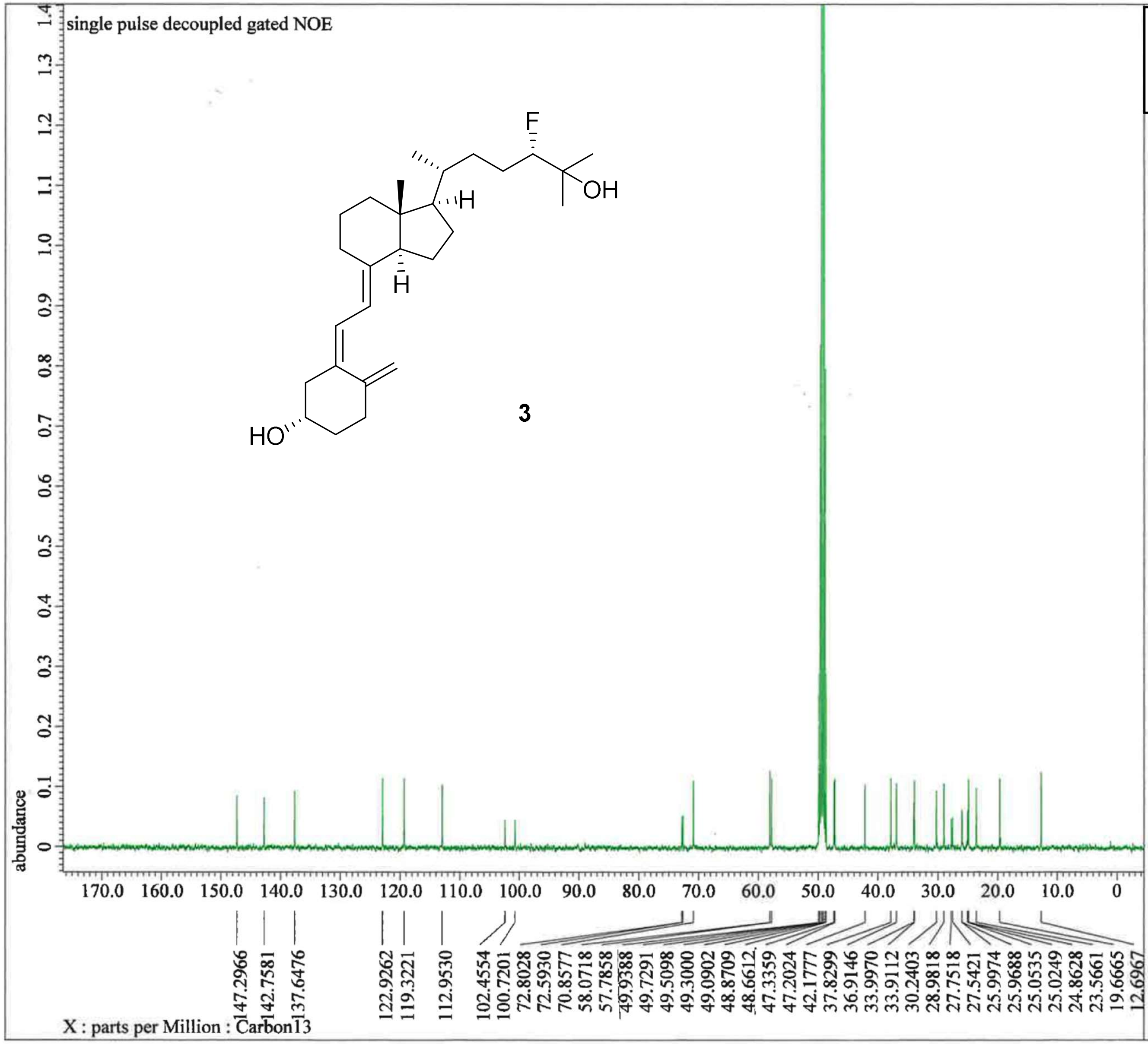

Filename	= 41KWG200116-2_Carbon_copy3
Author	= y-yakka
Experiment	= carbon.jxp
Sample_Id	= 41KWG200116-2
Solvent	= METHANOL-D4
Creation_Time	= 16-JAN-2020 13:20:40
Revision_Time	= 16-JAN-2020 14:03:55
Current_Time	= 16-JAN-2020 14:04:08
Comment	= single pulse decoupled gat
Data_Format	= 1D COMPLEX
Dim_Size	= 26214
Dim_Title	= Carbon13
Dim_Units	= [ppm]
Dimensions	= X
Site	= JNM-ECS400
Spectrometer	= DELTA2_NMR
Field_Strength	= 9.389766[T] (400[MHz])
X_Acq_Duration	= 0[s]
X_Domain	= 13C
X_Freq	= 100.52530333[MHz]
X_Offset	= 100[ppm]
X_Points	= 32768
X_Prescans	= 4
X_Resolution	= 0.95846665[Hz]
X_Sweep	= 31.40703518[kHz]
X_Sweep_Clipped	= 25.12562814[kHz]
Irr_Domain	= Proton
Irr_Freq	= 399.78219838[MHz]
Irr_Offset	= 5[ppm]
Clipped	= TRUE
Incomplete_Copy	= TRUE
Scans	= 771
Total_Scans	= 771
Relaxation_Delay	= 2[s]
Recvr_Gain	= 60
Temp_Get	= 23.5[dC]
X_90_Width	= 9.7[us]
X_Acq_Time	= 1.04333312[s]
X_Angle	= 30[deg]
X_Atn	= 3[dB]
X_Pulse	= 3.23333333[us]
Irr_Atn_Dec	= 25.6[dB]
Irr_Atn_Noe	= 25.6[dB]
Irr_Noise	= WALTZ
Irr_Pwidth	= 0.115[ms]
Decoupling	= TRUE
Initial_Wait	= 1[s]
Noe	= TRUE
Noe_Time	= 2[s]
Repetition_Time	= 3.04333312[s]

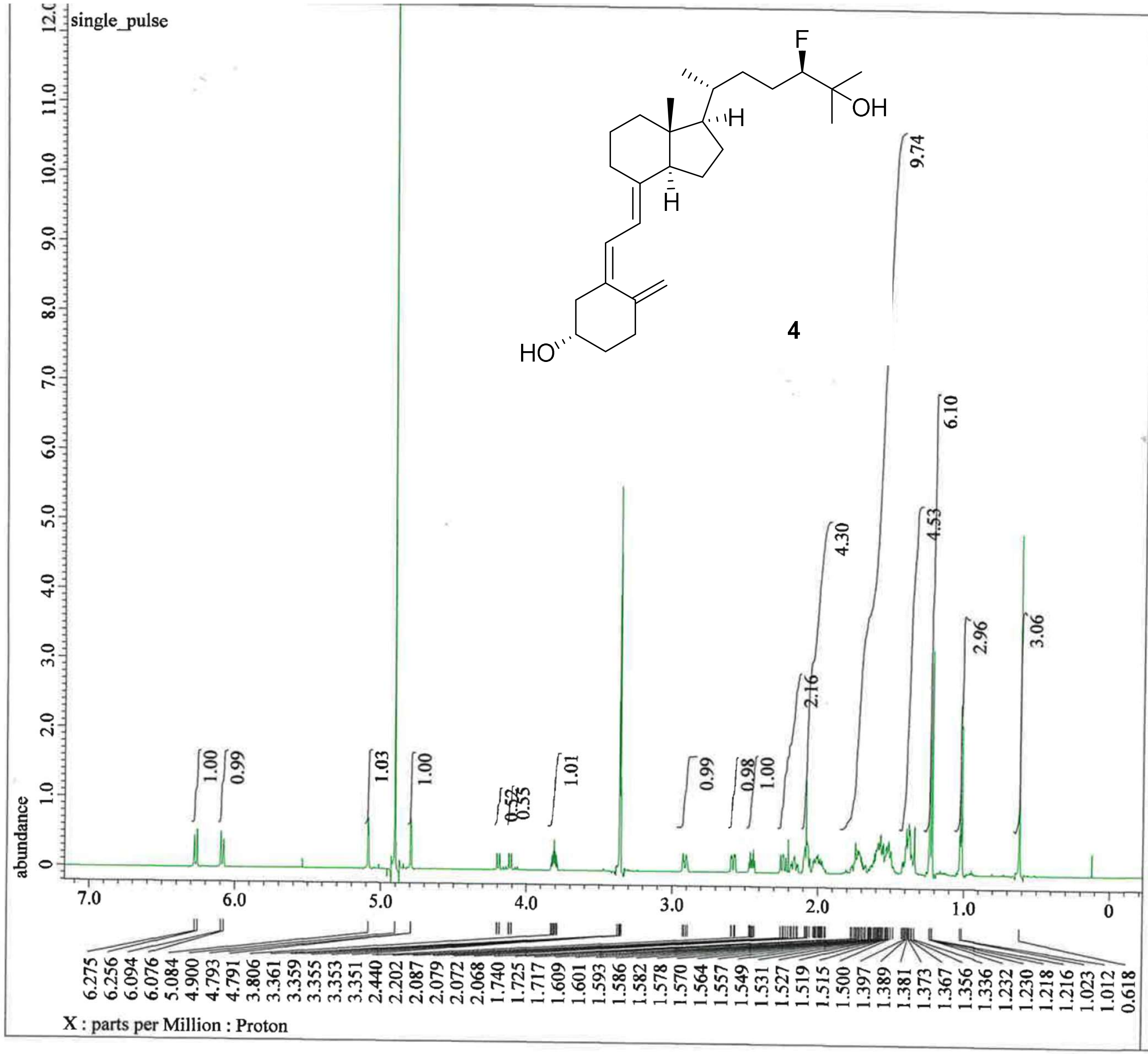

Filename	= 41KWG192014-1_Proton-1-5.j
Author	= y-yakka
Experiment	= proton.jxp
Sample_Id	= 41KWG192014-1
Solvent	= METHANOL-D4
Creation_Time	= 14-JAN-2020 10:31:46
Revision_Time	= 14-JAN-2020 10:45:17
Current_Time	= 14-JAN-2020 10:45:42
Comment	= single_pulse
Data_Format	= 1D COMPLEX
Dim_Size	= 13107
Dim_Title	= Proton
Dim_Units	= [ppm]
Dimensions	= X
Site	= JNM-ECA600
Spectrometer	= DELTA2_NMR
Field_Strength	= 14.09636928[T] (600[MHz])
X_Acq_Duration	= 1.4548992[s]
X_Domain	= 1H
X_Freq	= 600.1723046[MHz]
X_Offset	= 5[ppm]
X_Points	= 16384
X_Prescans	= 1
X_Resolution	= 0.68733284[Hz]
X_Sweep	= 11.26126126[kHz]
X_Sweep_Clipped	= 9.00900901[kHz]
Irr_Domain	= Proton
Irr_Freq	= 600.1723046[MHz]
Irr_Offset	= 5[ppm]
Tri_Domain	= Proton
Tri_Freq	= 600.1723046[MHz]
Tri_Offset	= 5[ppm]
Clipped	= FALSE
Scans	= 8
Total_Scans	= 8
Relaxation_Delay	= 5[s]
Recvr_Gain	= 42
Temp_Get	= 23.6[dC]
X_90_Width	= 8.1[us]
X_Acq_Time	= 1.4548992[s]
X_Angle	= 45[deg]
X_Atn	= 2.5[dB]
X_Pulse	= 4.05[us]
Irr_Mode	= Off
Tri_Mode	= Off
Dante_Presat	= FALSE
Initial_Wait	= 1[s]
Repetition_Time	= 6.4548992[s]

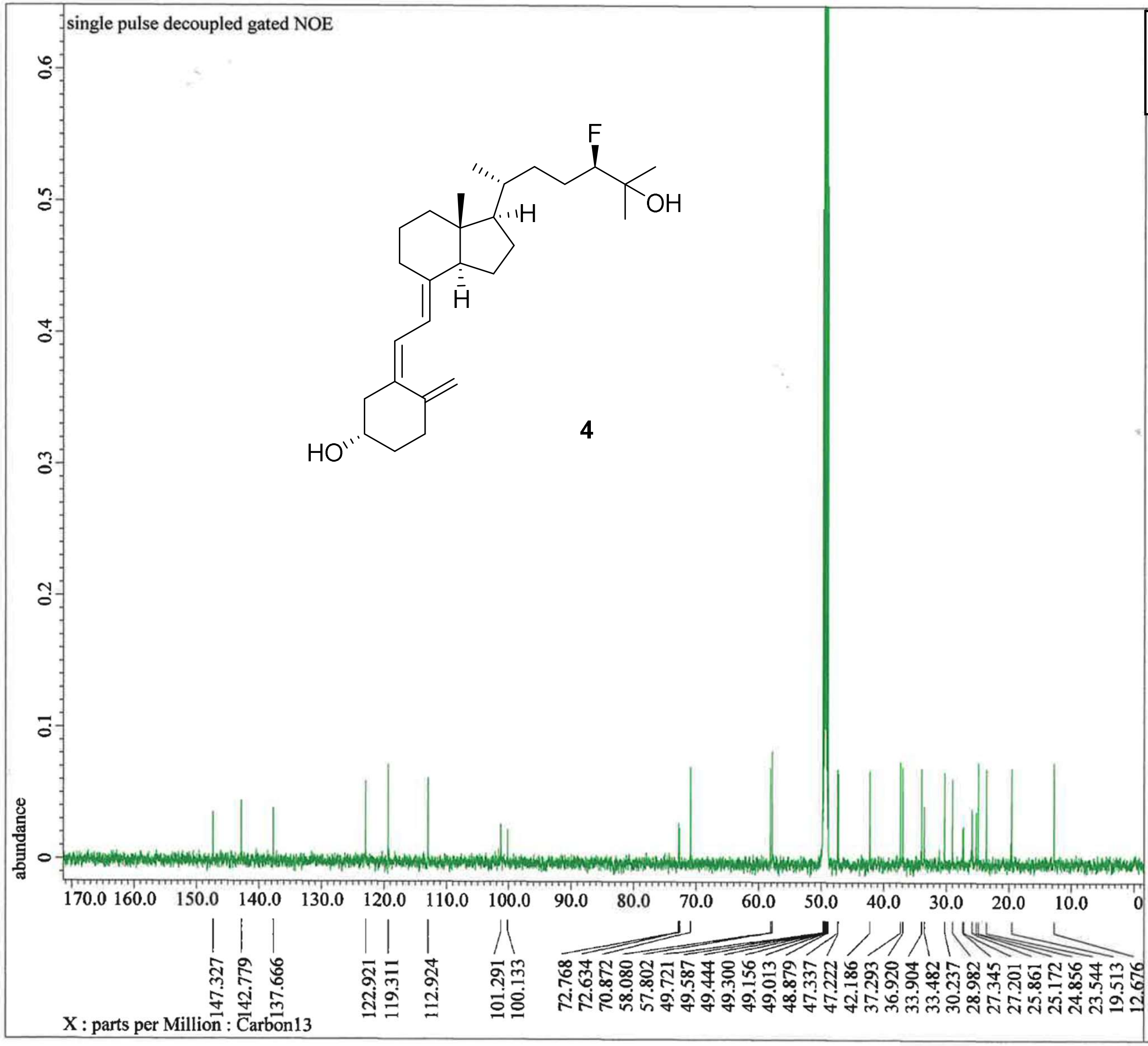

Filename	= 41KWG192014-1_Carbon-1-3.j
Author	= y-yakka
Experiment	= carbon.jxp
Sample_Id	= 41KWG192014-1
Solvent	= METHANOL-D4
Creation_Time	= 14-JAN-2020 10:35:38
Revision_Time	= 14-JAN-2020 11:28:29
Current_Time	= 14-JAN-2020 11:29:47
Comment	= single pulse decoupled gat
Data_Format	= 1D COMPLEX
Dim_Size	= 26214
Dim_Title	= Carbon13
Dim_Units	= [ppm]
Dimensions	= X
Site	= JNM-ECA600
Spectrometer	= DELTA2_NMR
Field_Strength	= 14.09636928[T] (600[MHz])
X_Acq_Duration	= 0.69206016[s]
X_Domain	= 13C
X_Freq	= 150.91343039[MHz]
X_Offset	= 100[ppm]
X_Points	= 32768
X_Prescans	= 4
X_Resolution	= 1.44496109[Hz]
X_Sweep	= 47.34848485[kHz]
X_Sweep_Clipped	= 37.87878788[kHz]
Irr_Domain	= Proton
Irr_Freq	= 600.1723046[MHz]
Irr_Offset	= 5[ppm]
Clipped	= FALSE
Scans	= 1024
Total_Scans	= 1024
Relaxation_Delay	= 2[s]
Recvr_Gain	= 58
Temp_Get	= 22.8[dC]
X_90_Width	= 11.5[us]
X_Acq_Time	= 0.69206016[s]
X_Angle	= 30[deg]
X_Atn	= 12[dB]
X_Pulse	= 3.83333333[us]
Irr_Atn_Dec	= 21.5[dB]
Irr_Atn_Noe	= 21.5[dB]
Irr_Noise	= WALTZ
Irr_Pwidth	= 76[us]
Decoupling	= TRUE
Initial_Wait	= 1[s]
Noe	= TRUE
Noe_Time	= 2[s]
Repetition_Time	= 2.69206016[s]

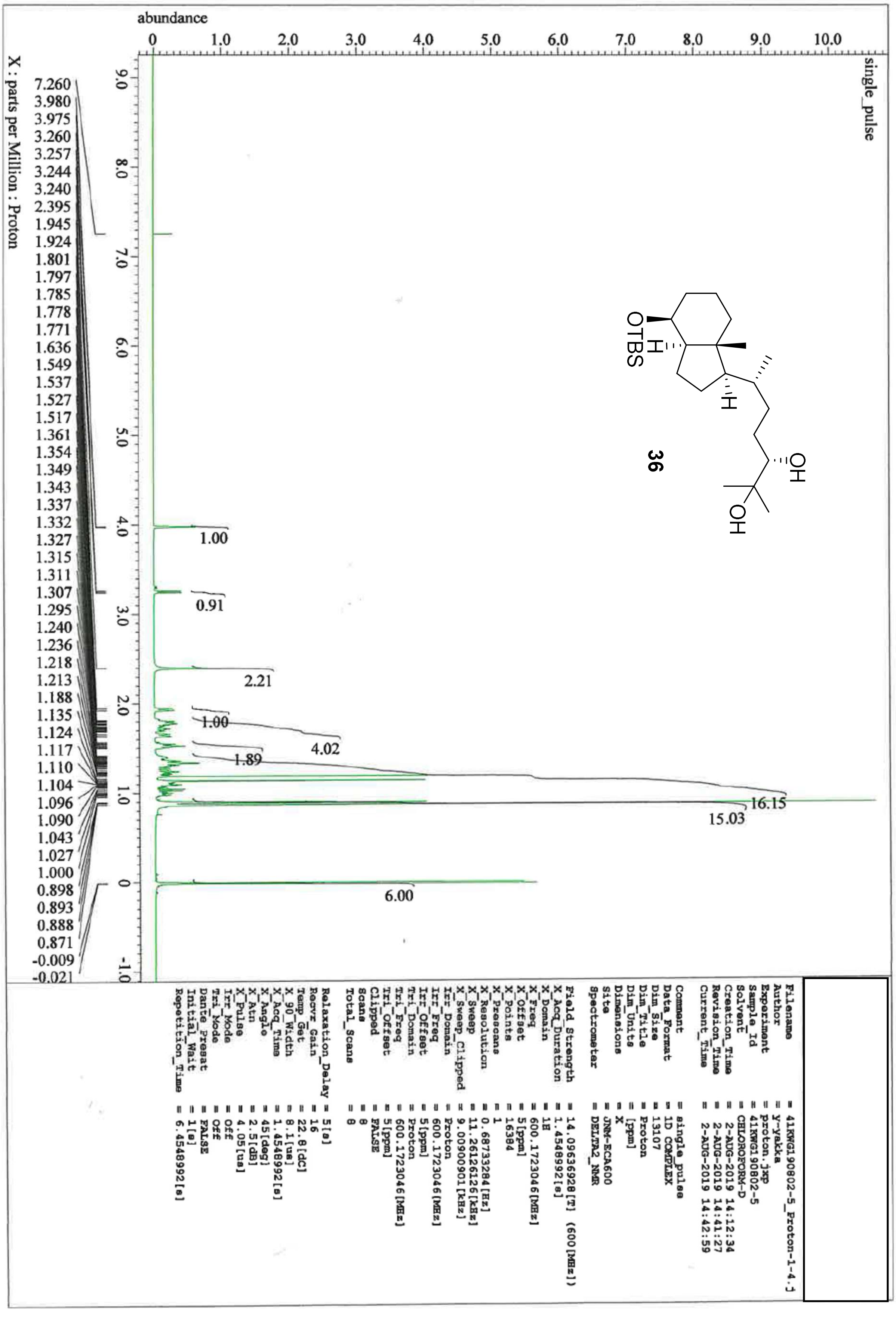
abundance
0 1.0 2.0 3.0 4.0 5.0 6.0 7.0 8.0 9.0 10.0
single_pulse
X : parts per Million : Proton
9.0 8.0 7.0 6.0 5.0 4.0 3.0 2.0 1.0 0 -1.0
7.260
3.980
3.975
3.260
3.257
3.244
3.240
2.395
1.945
1.924
1.801
1.797
1.785
1.778
1.771
1.636
1.549
1.537
1.527
1.517
1.361
1.354
1.349
1.343
1.337
1.332
1.327
1.315
1.311
1.307
1.295
1.240
1.236
1.218
1.213
1.188
1.135
1.124
1.117
1.110
1.104
1.096
1.090
1.043
1.027
1.000
0.898
0.893
0.888
0.871
-0.009
-0.021
1.00
0.91
2.21
1.00
4.02
1.89
16.15
15.03
6.00
OTBS
H
H
OH
OH
36
Filename = 41KWG190802-5_Proton-1-4.j
Author = y-yakka
Experiment = proton.jxp
Sample_Id = 41KWG190802-5
Solvent = CHLOROFORM-D
Creation_Time = 2-AUG-2019 14:12:34
Revision_Time = 2-AUG-2019 14:41:27
Current_Time = 2-AUG-2019 14:42:59
Comment = single_pulse
Data_Format = 1D COMPLEX
Dim_Size = 13107
Dim_Title = Proton
Dim_Units = [ppm]
Dimensions = X
Site = JNM-ECA600
Spectrometer = DELTA2_NMR
Field_Strength = 14.09636928[T] (600[MHz])
X_Acq_Duration = 1.45489992[s]
X_Domain = 1H
X_Freq = 600.1723046[MHz]
X_Offset = 5[ppm]
X_Points = 16384
X_Prescans = 1
X_Resolution = 0.68733284[Hz]
X_Sweep = 11.26126126[kHz]
X_Sweep_Clipped = 9.00900901[kHz]
Irr_Domain = Proton
Irr_Freq = 600.1723046[MHz]
Irr_Offset = 5[ppm]
Tri_Domain = Proton
Tri_Freq = 600.1723046[MHz]
Tri_Offset = 5[ppm]
Clipped = FALSE
Scans = 8
Total_Scans = 8
Relaxation_Delay = 5[s]
Recvr_Gain = 16
Temp_Get = 22.8[dC]
X_90_Width = 8.1[us]
X_Acq_Time = 1.45489992[s]
X_Angle = 45[deg]
X_Atn = 2.5[dB]
X_Pulse = 4.05[us]
Irr_Mode = Off
Tri_Mode = Off
Dante_Presat = FALSE
Initial_Wait = 1[s]
Repetition_Time = 6.45489992[s]

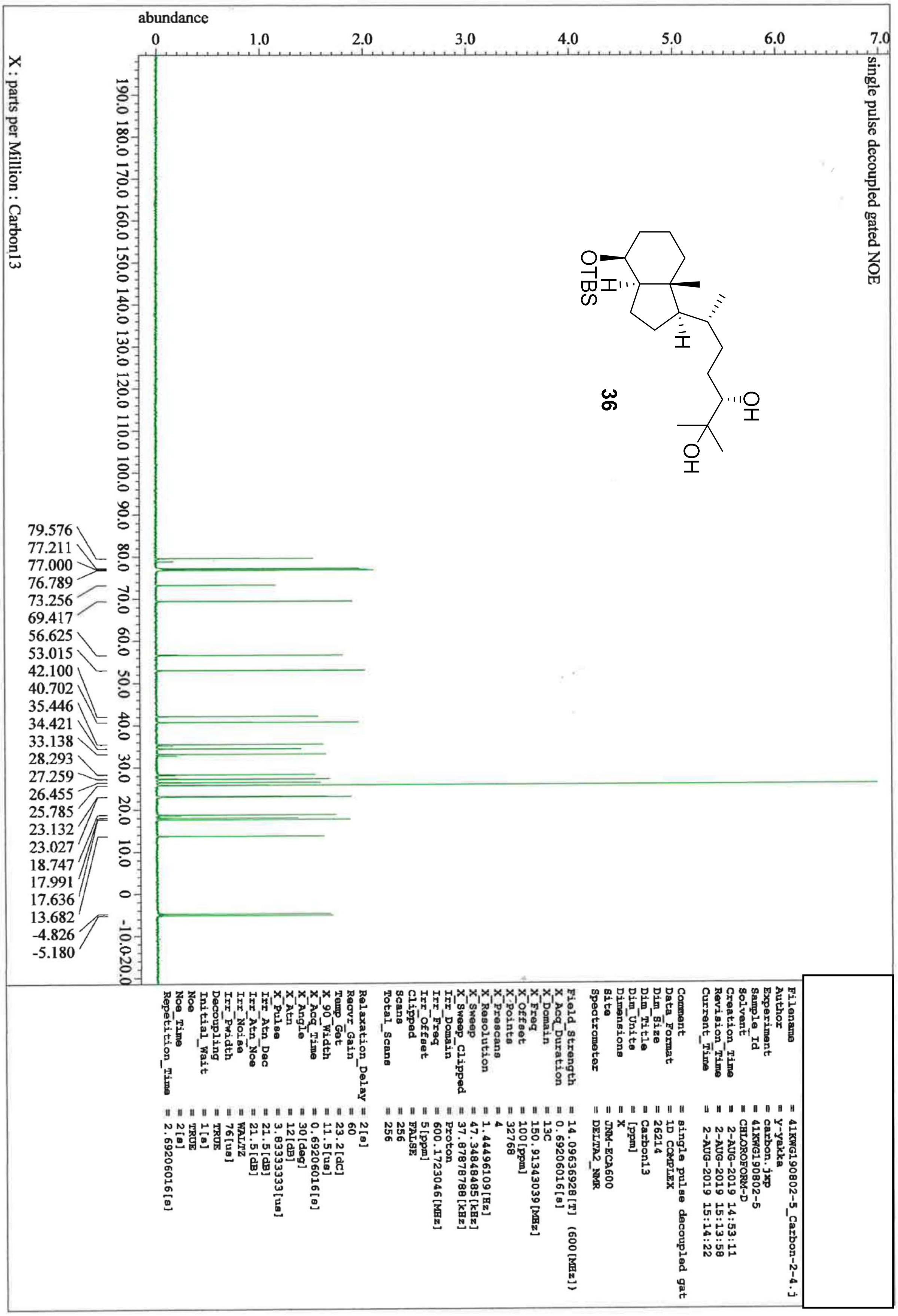
single pulse decoupled gated NOE
abundance
0
1.0
2.0
3.0
4.0
5.0
6.0
7.0
36
OTBS
H
H
OH
OH
X : parts per Million : Carbon13
190.0 180.0 170.0 160.0 150.0 140.0 130.0 120.0 110.0 100.0 90.0 80.0 70.0 60.0 50.0 40.0 30.0 20.0 10.0 0 -10.0 20.0
79.576
77.211
77.000
76.789
73.256
69.417
56.625
53.015
42.100
40.702
35.446
34.421
33.138
28.293
27.259
26.455
25.785
23.132
23.027
18.747
17.991
17.636
13.682
-4.826
-5.180
Filename = 41KWG190802-5_Carbon-2-4.j
Author = y-yakka
Experiment = carbon.jxp
Sample_Id = 41KWG190802-5
Solvent = CHLOROFORM-D
Creation_Time = 2-AUG-2019 14:53:11
Revision_Time = 2-AUG-2019 15:13:58
Current_Time = 2-AUG-2019 15:14:22
Comment = single pulse decoupled gat
Data_Format = 1D COMPLEX
Dim_Size = 26214
Dim_Title = Carbon13
Dim_Units = [ppm]
Dimensions = X
Site = JNM-ECA600
Spectrometer = DELTA2_NMR
Field_Strength = 14.09636928[T] (600[MHz])
X_Acq_Duration = 0.69206016[s]
X_Domain = 13C
X_Freq = 150.91343039[MHz]
X_Offset = 100[ppm]
X_Points = 32768
X_Prescans = 4
X_Resolution = 1.44496109[Hz]
X_Sweep = 47.34848485[kHz]
X_Sweep_Clipped = 37.87878788[kHz]
Irr_Domain = Proton
Irr_Freq = 600.17230046[MHz]
Irr_Offset = 5[ppm]
Clipped = FALSE
Scans = 256
Total_Scans = 256
Relaxation_Delay = 2[s]
Recvr_Gain = 60
Temp_Get = 23.2[dC]
X_90_Width = 11.5[us]
X_Acq_Time = 0.69206016[s]
X_Angle = 30[deg]
X_Atn = 12[dB]
X_Pulse = 3.83333333[us]
Irr_Atn_Dec = 21.5[dB]
Irr_Atn_Noe = 21.5[dB]
Irr_Noise = WALTZ
Irr_Pwidth = 76[us]
Decoupling = TRUE
Initial_Wait = 1[s]
Noe = TRUE
Noe_Time = 2[s]
Repetition_Time = 2.69206016[s]

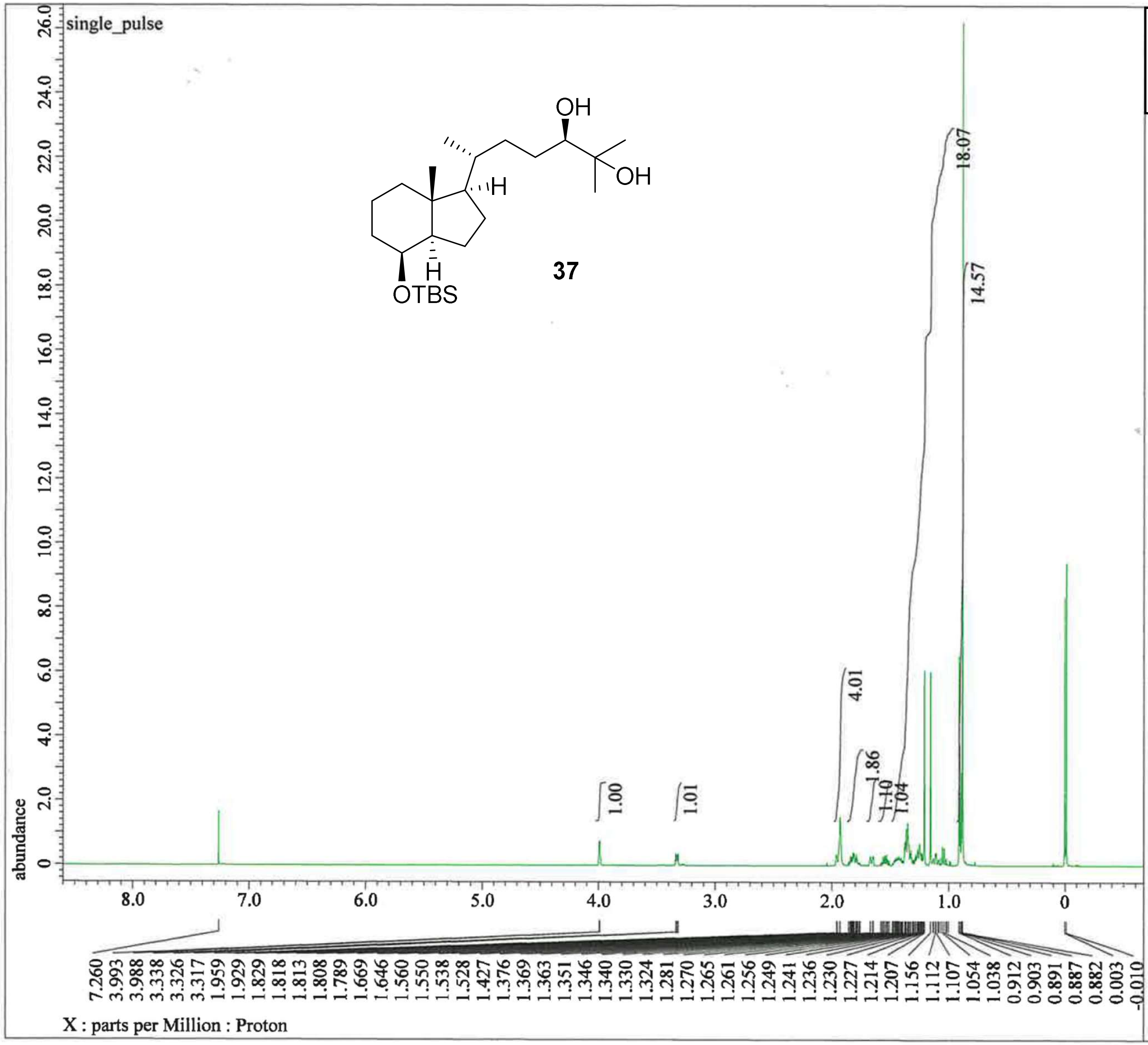


```
Filename          = 41KWG190705-1_Proton-1-10.
Author            = y-yakka
Experiment        = proton.jxp
Sample_Id         = 41KWG190705-1
Solvent           = CHLOROFORM-D
Creation_Time     =  5-JUL-2019 14:19:51
Revision_Time     =  5-JUL-2019 14:57:20
Current_Time      =  5-JUL-2019 14:57:31

Comment           = single_pulse
Data_Format       = 1D COMPLEX
Dim_Size          = 13107
Dim_Title         = Proton
Dim_Units         = [ppm]
Dimensions        = X
Site              = JNM-ECA600
Spectrometer      = DELTA2_NMR

Field_Strength    = 14.09636928[T] (600[MHz])
X_Acq_Duration    = 1.4548992[s]
X_Domain          = 1H
X_Freq            = 600.1723046[MHz]
X_Offset          = 5[ppm]
X_Points          = 16384
X_Prescans        = 1
X_Resolution      = 0.68733284[Hz]
X_Sweep           = 11.26126126[kHz]
X_Sweep_Clipped   = 9.00900901[kHz]
Irr_Domain        = Proton
Irr_Freq          = 600.1723046[MHz]
Irr_Offset        = 5[ppm]
Tri_Domain        = Proton
Tri_Freq          = 600.1723046[MHz]
Tri_Offset        = 5[ppm]
Clipped           = FALSE
Scans             = 8
Total_Scans       = 8

Relaxation_Delay  = 5[s]
Recvr_Gain        = 30
Temp_Get          = 23.1[dC]
X_90_Width        = 8.1[us]
X_Acq_Time        = 1.4548992[s]
X_Angle           = 45[deg]
X_Atn             = 2.5[dB]
X_Pulse           = 4.05[us]
Irr_Mode          = Off
Tri_Mode          = Off
Dante_Presat      = FALSE
Initial_Wait      = 1[s]
Repetition_Time   = 6.4548992[s]
```

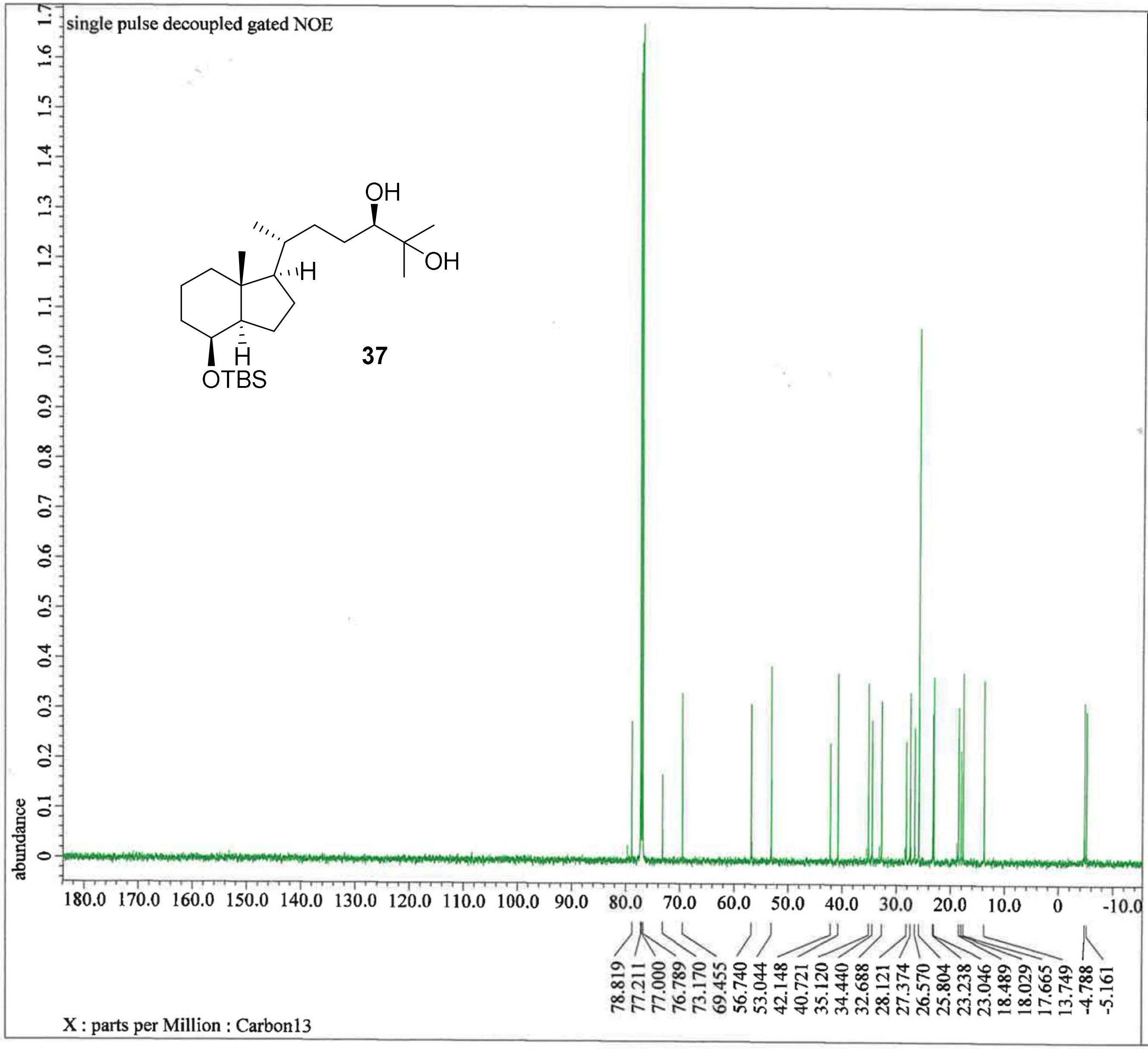


```
Filename           = 41KWG190705-1_Carbon-1-4.j
Author             = y-yakka
Experiment         = carbon.jxp
Sample_Id          = 41KWG190705-1
Solvent            = CHLOROFORM-D
Creation_Time      =  5-JUL-2019 14:24:18
Revision_Time      =  5-JUL-2019 14:49:27
Current_Time       =  5-JUL-2019 14:51:52

Comment            = single pulse decoupled gat
Data_Format        = 1D COMPLEX
Dim_Size           = 26214
Dim_Title          = Carbon13
Dim_Units          = [ppm]
Dimensions         = X
Site               = JNM-ECA600
Spectrometer       = DELTA2_NMR

Field_Strength     = 14.09636928[T] (600[MHz])
X_Acq_Duration     = 0.69206016[s]
X_Domain           = 13C
X_Freq             = 150.91343039[MHz]
X_Offset           = 100[ppm]
X_Points           = 32768
X_Prescans         = 4
X_Resolution       = 1.44496109[Hz]
X_Sweep            = 47.34848485[kHz]
X_Sweep_Clipped    = 37.87878788[kHz]
Irr_Domain         = Proton
Irr_Freq           = 600.1723046[MHz]
Irr_Offset         = 5[ppm]
Clipped            = FALSE
Scans              = 512
Total_Scans        = 512

Relaxation_Delay   = 2[s]
Recvr_Gain         = 58
Temp_Get           = 23.3[dC]
X_90_Width         = 11.5[us]
X_Acq_Time         = 0.69206016[s]
X_Angle            = 30[deg]
X_Atn              = 12[dB]
X_Pulse            = 3.83333333[us]
Irr_Atn_Dec        = 21.5[dB]
Irr_Atn_Noe        = 21.5[dB]
Irr_Noise          = WALTZ
Irr_Pwidth         = 76[us]
Decoupling         = TRUE
Initial_Wait       = 1[s]
Noe                = TRUE
Noe_Time           = 2[s]
Repetition_Time    = 2.69206016[s]
```
